# Structural Analysis of Hypothetical Proteins from *Helicobacter pylori*: An Approach to Estimate Functions of Unknown or Hypothetical Proteins

**DOI:** 10.3390/ijms13067109

**Published:** 2012-06-08

**Authors:** Sung Jean Park, Woo Sung Son, Bong-Jin Lee

**Affiliations:** 1 College of Pharmacy, Gachon University, 534-2 Yeonsu 3-dong, Yeonsu-gu, Incheon 406-799, Korea; E-Mail: psjnmr@gachon.ac.kr; 2 College of Pharmacy, CHA University, 120 Haeryong-ro, Pocheon-si, Gyeonggi-do 487-010, Korea; E-Mail: wson@cha.ac.kr; 3 Research Institute of Pharmaceutical Sciences, College of Pharmacy, Seoul National University, San 56-1, Shillim-Dong, Kwanak-Gu, Seoul 151-742, Korea

**Keywords:** *Helicobacter pylori*, structural genomics, NMR, X-ray, unknown protein, hypothetical protein, structural homology

## Abstract

*Helicobacter pylori* (*H. pylori*) have a unique ability to survive in extreme acidic environments and to colonize the gastric mucosa. It can cause diverse gastric diseases such as peptic ulcers, chronic gastritis, mucosa-associated lymphoid tissue (MALT) lymphoma, gastric cancer, *etc*. Based on genomic research of *H. pylori*, over 1600 genes have been functionally identified so far. However, *H. pylori* possess some genes that are uncharacterized since: (i) the gene sequences are quite new; (ii) the function of genes have not been characterized in any other bacterial systems; and (iii) sometimes, the protein that is classified into a known protein based on the sequence homology shows some functional ambiguity, which raises questions about the function of the protein produced in *H. pylori*. Thus, there are still a lot of genes to be biologically or biochemically characterized to understand the whole picture of gene functions in the bacteria. In this regard, knowledge on the 3D structure of a protein, especially unknown or hypothetical protein, is frequently useful to elucidate the structure-function relationship of the uncharacterized gene product. That is, a structural comparison with known proteins provides valuable information to help predict the cellular functions of hypothetical proteins. Here, we show the 3D structures of some hypothetical proteins determined by NMR spectroscopy and X-ray crystallography as a part of the structural genomics of *H. pylori*. In addition, we show some successful approaches of elucidating the function of unknown proteins based on their structural information.

## 1. *H. pylori* as a Pathogen

*Helicobacter pylorus is* one of the pathogens involved in various gastric diseases such as peptic ulcers, chronic gastritis, mucosa-associated lymphoid tissue lymphoma, and gastric cancer [1–3]. Infection with *H. pylori* is associated with an increased risk of gastric adenocarcinoma and has attracted attention as a cofactor in the pathogenesis of this malignant condition [4]. Moreover, the risk of developing cancer is related to the physiologic and histologic changes induced by a *H. pylori* infection in the stomach [5]. Despite a general decline in the incidence of gastric cancer, it remains the fourth most common cancer and second leading cause of cancer-related deaths worldwide [6]. However, most *H. pylori* infections do not cause cancer. The sporadic distribution of the disease caused by *H. pylori* looks to be dependent on host-related factors: the host (human individual) genetics controlling the inflammatory response, the age when the *H. pylori* infection was acquired, poor nutrition, storage of food, and the pattern of food consumption can be considered as host-related factors [7–9].

In addition, bacterial factors associated with the risk of gastric cancer are also emphasized, and molecular and cell biology approaches aimed at understanding the interaction between *H. pylori* and transforming epithelial cells have been carried out. Since *H. pylori* is a highly heterogeneous bacterial species, both genotypically and phenotypically, and is highly adapted for survival in the gastric niche, it is not easy to figure out the major bacterial factors that are directly associated with etiopathogenesis [10,11]. Based on the current knowledge, several virulence factors such as genes within the cag (cytotoxin-associated antigen) pathogenicity island, including the gene encoding the CagA protein, as well as polymorphic variation in the VacA vacuolating exotoxin and the blood group antigen binding adhesions, BabA and SabA, are regarded as possible bacterial factors [6,10,12]. A duodenal ulcer-promoting gene (*dupA*), located in the “plasticity region” of the *H. pylori* genome, was reported as a potential virulence marker [10,13]. Other bacterial factors such as peptidoglycan, lipopolysaccharide(LPS), γ-glutamyl trans-peptidase(GGT), and protease HtrA may be linked to pathogenicity [14].

Although a huge amount of biological data on *H. pylori* has been accumulated, enzymes or proteins of unknown function still make up more than a third of the open reading frames (ORF) of *H. pylori*. An unknown protein could be defined as a protein whose function has not yet been characterized, and a hypothetical protein could be defined as a protein that is supposed to exist in an organism although its existence has not been shown experimentally. Therefore, in a broad sense, hypothetical proteins could be included in unknown proteins. To completely understand the pathogenic mechanism of *H. pylori*, it is very important to elucidate the functions of these unknown proteins. To fill in the “missing parts list” is accordingly one of the greatest challenges for post-genomic biology, and a tremendous opportunity to discover new biological and pathogenic machinery in *H. pylori*.

## 2. *H. pylori* Genomic Sequence

The sequencing of the *H. pylori* genome started in 1997 with the *H. pylori* strain 26695 [15]. It was isolated from an English patient with chronic gastritis. The chromosome of strain 26695 is circular and composed of 1.67 mega base pairs (Table 1). The average G-C content is approximately 38.9% and the genome has 1590 open reading frames (ORF) that are possibly protein-coding loci [1], together with the RNA coding genes (2 copies of 16S rRNA and 23S rRNA genes, 36 tRNA genes). From the following analysis of the same genome, it was suggested that a smaller number of ORFs is in the sequence of strain 26695 [16].

Ongoing studies have found genes that were missing in previous analyses, as in the case of SecE. A general secretion machinery is widely present in bacteria, which functions in the secretion of outer membrane proteins to extracellular environments [18]. From the first annotation results, it was thought that strain 26695 had only a partial general secretion machinery because it lacked SecE [15]. A new small open reading frame between *nusG* and *rmpG* (HP1203–HP1204) in the genome sequences was found using an *ab initio* server, GeneMark, Glimmer, and BlastX [19]. It has a high homology and structural similarity to the SecE protein in related bacteria implying that strain 26695 has a complete general secretion machinery. In addition, small RNA genes are universally present in bacteria [20]. The tmRNA gene (*ssrA*) has been found in *H. pylori*, encoding a functional RNA molecule and a small peptide involved in the quality control of translation [21]. In addition, the *H. pylori* strain contains a sRNA gene encoding the RNA component of RnaseP and the 4.5S RNA gene which is involved in secretion [22,23].

In 2008, the adaptations of *H. pylori* to a rarely captured event in the evolution of its impact on a host biology were characterized by defining the impact of these adaptations on an intriguing but poorly characterized interaction between this bacterium and gastric epithelial stem cells [24]. *H. pylori* HPKX_438_AG0C1 and HPXK_438_CA4C1 were isolated from a single patient who progressed from ChAG (chronic atrophic gastritis) to adenocarcinoma using a population-based endoscopy study. ChAG-associated Kx1 and Cancer-associated Kx2 genomes were analyzed to examine the adaptation of *H. pylori*, respectively. Micro-arrays gave a comprehensive view of the genome diversity of the *H. pylori* pathogen. This was performed with information on the origin of the *hspA* together with *glmM* alleles revealing that *H. pylori* infection may be acquired by more diverse routes than previously expected [25]. According to cluster analysis, isolates from family D belonged to three different strains, those from family L consisted of two strains, and those from family A were grouped into at least 5 strains. Strains from family D and family L differed by the presence/absence of 24 to 42 CDSs (coding sequences). In family A, one strain was difficult to define due to the small differences in gene profiles between neighboring branches.

In 2009, the complete genome sequence of *H. pylori* G27 was reported [26]. The G27 strain was originally isolated from an endoscopy patient from Italy [27]. The genome consists of a single circular chromosome with about 1.65 mega base pairs (Table 1) that is AT rich (61.6%), contains 1515 ORFs, and is similar in size and composition to the other published *H. pylori* genomes of strains 26695, J99, and HPAG [15,16,28]. The G27 strain contains 58 genes that are not found in 26695, J99, or HPAG, as defined by a blastp hit. The majority of these G27-specific genes are predicted to encode hypothetical proteins [26].

In the same year, the genome sequences of two *H. pylori* strains were analyzed [29]. *H. pylori* strain 98-10 was isolated from a patient with gastric cancer and strain B128 was isolated from a patient with gastric ulcer disease. Strain 98-10 was most closely related to *H. pylori* strains of East Asian origin and strain B128 was most closely related to strains of European origin. Strain 98-10 contained multiple features characteristic of East Asian strains, including a type s1c *vacA* allele and a *cagA* allele encoding an EPIYA-D tyrosine phosphorylation motif.

Very recently, several genome sequences of different strains were reported accelerating *H. pylori* genomic and proteomic research [30–38]. Strain 908 is a close relative strain of J99 [39] and was isolated from an African patient living in France, who suffered from duodenal ulcer disease [40]. The B8 strain consists of about 1.67 mega base pairs and a small plasmid of about 6000 base pairs carrying nine putative genes. Interestingly, the B8 strain contains coding sequences, 293 of which are strain-specific, coding mainly for hypothetical proteins with unknown functions [31]. Similarly, the P12 strain contains plasticity zones, encoding for the type IV secretion system and having the typical properties of genomic islands [32]. Another sequenced genome, the Shi470 strain known as the Shiimaa village strain was more Asian- than European-like genome-wide, indicating Amerind ancestry. This strain contains two unique *cagA* virulence genes and a novel allele of gene hp0519 encoding host tissue interaction protein [33]. There are several *H. pylori* populations such as *hpAfrical*, *hpEurope*, *hspEAsia*, and *hspAmerind* because this bacterium has colonized the stomach since early in human evolution and diverged with ancient human migrations [41–43]. One of these populations, the *hspAmerind* strain V225d, was cultured from a Venezuelan Piaroa Amerindian subject and identified. The V225d strain is cag-positive encoding a multifunctional effector protein injected into host cells by the cag type IV secretion system [34]. Two strains, 2017 and 2018, are the chronological subclones of strain 908 and cultured from the antrum and corpus, respectively. Using comparative genomic analysis [35,37], these two strains are almost identical and descended from the genome of strain 908 [30,36]. The B45 strain was sequenced from a gastric mucosa-associated lymphoid tissue (MALT) lymphoma patient and induced an integrated prophage in this strain by UV irradiation [38].

**Figure 1:**
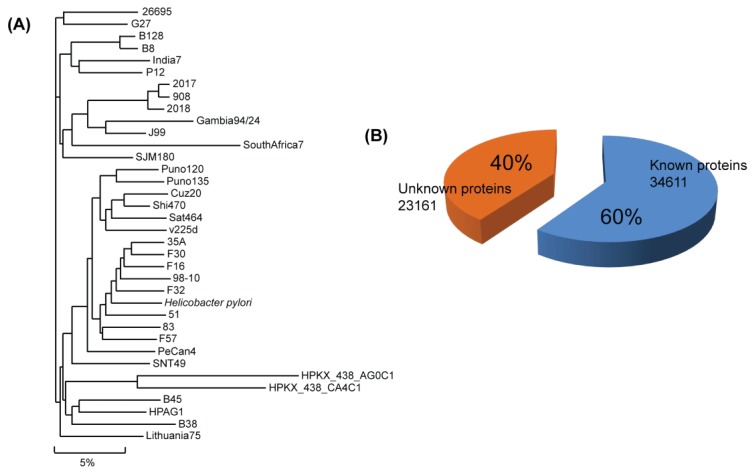
Genome sequence and proteins of *H. pylori*. In the phylogenetic tree, a total of 36 sub-species are branched with a total of about 60,000 genes (**A**); and among the translated proteins, the biological functions of 40% of the proteins are unidentified (**B**).

The Comprehensive Microbial Resource (CMR) is a free tool that allows researchers to access all of the publicly available bacterial genome sequences completed to date [44] (Figure 1). Currently, it provides genomic sequences of three strains of *Helicobacter pylori* (26695, HPAG1, J99).

## 3. Structural Reports on *H. pylori* Proteins

As in the case of other genomic research, Structural Genomics Initiatives are mainly responsible for determination of *H. pylori* protein structures. These initiatives, together with the structure determination of known proteins, have made enormous strides in the elucidation of unknown protein structure of *H. pylori* [15,16,24–26,28–38,45–47]. The available structural data have already led to the identification of potentially new drug targets [48] and has been helpful in assigning functions to proteins of which the functions were previously unknown [49,50].

The increase in structure determination for *H. pylori* has been triggered by the sequencing of the *H. pylori* 52 and 26695 genomes [15,25,45,47]. The genome sequences and their protein structures yielded many clues to help understand the pathogenesis of *H. pylori*. Approximately 14% of Lyase structures have been determined and represent the largest proportion of any functional class of which the structures have already been solved (Table S1).

**Figure 2:**
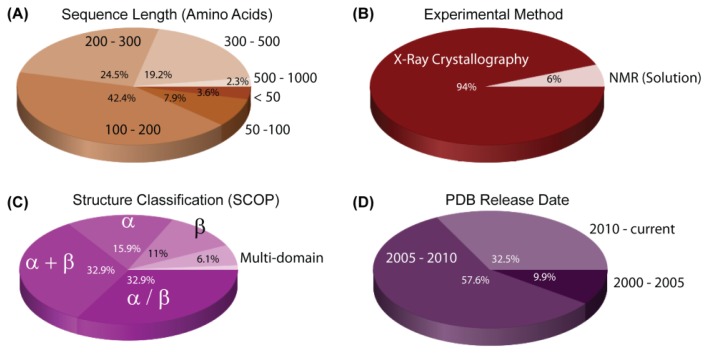
Statistics of protein structures from *H. pylori*. All data were collected and processed from PDB on 14 February 2012 [52]. The dominant properties of the presented data are 100–300 kDa in size, X-ray diffraction as the experimental method, alpha and beta structural motifs, and release date from 2005–2010.

The sequencing of the genome led to a dramatic increase in the number of known structures for *H. pylori* proteins deposited in the Protein Data Bank (PDB) (Figure 2). The first *H. pylori* protein structure was determined in 2001 (PDB ID: 1G6O) [51]. In the following four years, 32 more structures were reported (Figure 2). After several sub-species genome sequences of *H. pylori* became publicly available, the number of structures determined after 2005 increased sharply and at an increasing rate.

Usually, protein solubility is one of the main bottlenecks in structure determination [53]. In the case of *H. pylori*, methods have already been developed that remedied this problem, such as the development of customized expression strategies for *H. pylori* proteins in *Escherichia coli* [54]. The increase in determined structures is also due to the development of improved methods for high-throughput X-ray crystallography. However, the major driving force for this increase was the availability of genome-wide sequence data in the early 2000s.

There are currently 79,356 structures in the PDB as of 14 February 2012, of which 0.35%, a total of 279, are structures of *H. pylori* proteins. Of these proteins, 28 are unknown in function, which represents 10.03% of the determined *H. pylori* structures (Table 2).

**Figure 3:**
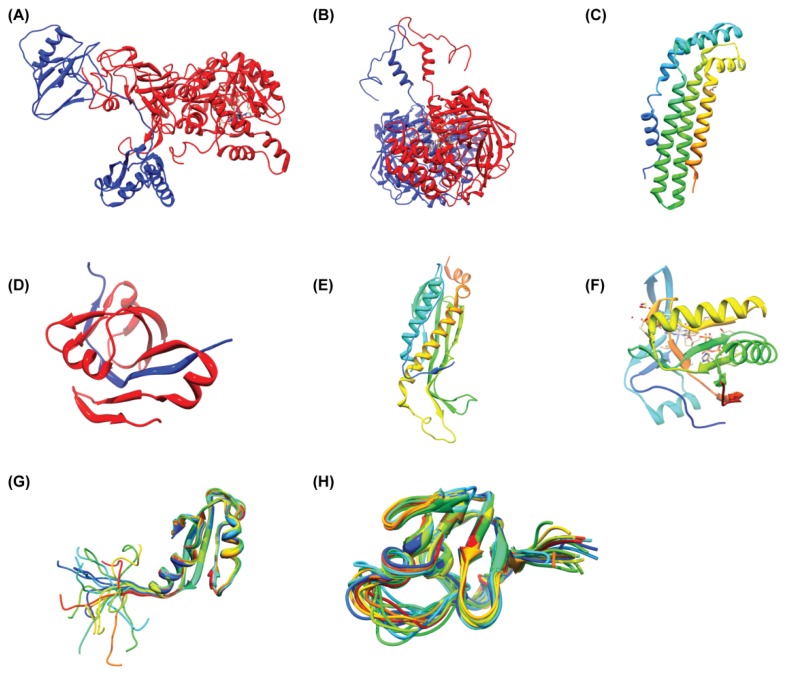
Several 3D structures from *H. pylori*. Urease subunit α and β (**A**, pdb code: 1E9Y), Kat catalase (**B**, pdb code: 1WQL) are multiple domain structures with multiple chains. Aspartate 1-decarboxylase adopts a dominant β structure (**D**, pdb code: 1UHD). The structures of unknown proteins are shown with different variations of their structural domains (**C**, pdb code 1S2X, all α; **E**, pdb code: 2I9I, α/β; **F**, pdb code: 2ATZ, α + β; **G**, pdb code: 2H9Z, α + β; **H**, pdb code: 2K6P, RNA binding motif). Structures of **G** and **H** are solved by NMR. All structures were displayed using UCSF Chimera with ribbon presentation method [55].

A complete list of *H. pylori* protein structures deposited in the PDB is given in the Supporting Information Table S1. The predominant method used to determine these structures was X-ray crystallography, which accounts for 261 of the total number of *H. pylori* structures currently determined (Figure 2). A further 18 were elucidated by solution-state NMR spectroscopy. Most structures are of individual proteins, although many are bound by small molecule ligands such as substrate analogues and only 11 protein-DNA complexes have been determined (Figure 3, Table S1).

## 4. Unknown Proteins in *H. pylori* and Estimation of Their Function

The most typical approach of predicting the function of an unknown protein is to use sequence similarity by finding a similar protein of known function [56]. Based on sequence-similarity, a predictor assigns the known function to the inferred protein. Actually, the functions of enzymes tend to be conserved if they share more than a 40%–50% sequence identity. The sequence-based approach is reasonable, however, approximately 50% of the unknown proteins from a newly sequenced genome could not be assigned to their function using only sequence-similarity approaches [57] (Figure 1). The low efficiency of the sequence-similarity search may be partly caused by gene sequences that are quite new and genes that have not yet been characterized in other bacterial systems. To overcome the weakness of sequence-similarity searches, several trials were employed using so called “similarity free” methods [57]. The methods use physicochemical properties and secondary structure of proteins. Bioinformatics developed the methods and there have been successful cases for characterizing function or structure [58–60]. However, the methods need to be improved since similarity-free methods still depend to a certain extent on similarity.

Another approach to identify function is to use 3D structures. This approach often succeeds in cases where sequence-based methods fail. This may be due to the idea that in many cases evolution retains the folding pattern long after the sequence similarity becomes undetectable. Structural similarity searches use the global fold of the protein [61–64] or detect the functionally important regions of the protein [65–69]. Since structures diverge more slowly than sequences, a sequence comparison may be less sensitive than a structure comparison [70]. However, the structural comparison still has the limitation of false positives being reported and needs to be improved to overcome overestimation of statistical significance like sequence-similarity searches [70]. This means that experimental confirmation is still required for exact assignment of function to an unknown protein.

Some examples of functional elucidation of unknown proteins from *H. pylori* are provided below. For estimation, we generally conducted four steps: (i) structure determination; (ii) sequence homology search using PSI-BLAST [71]; (iii) structural homology search using the web server DALI [62]; and (iv) experimental confirmation of the function.

### 4.1. HP0894–HP0895: Toxin-Antitoxin System in *H. pylori*

**Figure 4:**
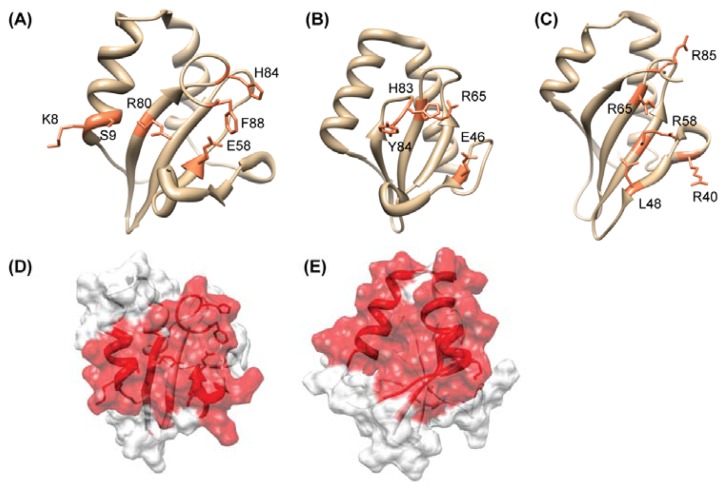
Comparison of the structural and catalytic residues of HP0894 with those of its structural homologues. **A**–**C**, ribbon displays of the representative conformer of HP0894; (**A**) *E. coli* YoeB (PDB ID: 2A6R); (**B**) *P. horikoshii* RelE (PDB ID: 1WMI); (**C**) labeled functional or predicted key residues are colored *coral*. The RelE monomer structure was extracted from the aRelE-aRelB complex structure; (**D**) Chemical shift perturbation mapping of the *C*-terminal peptide of HP0895-binding region on HP0894 (1:1 molar ratio). Ribbon and surface displays of HP0894 structure colored according to chemical shift perturbations. The changes of the residues in obvious slow or fast exchange modes are colored in *red*. (**E**) Chemical shift perturbation mapping of the ssDNA-U [d(ACACUAAGAA)]-binding region on HP0894 (1:4 molar ratio). Residues showing significant chemical shift changes are colored in *red*.

The high-quality NMR structure of HP0894 was reported [72]. The HP0894 structure (PDB ID: 1Z8M) has two α-helices, two 3_10_-helices, and four β-strands (α-α-3_10_-β-3_10_-β-β-β). The β-Strands form a four-stranded anti-parallel β-sheet (Figure 4). BLAST conserved domain search [73] showed that HP0894 contains the conserved domain DUF332 (Domain of Unknown Function), which is equivalent to COG 3041 in the National Center for Biotechnology Information Database of Clusters of Orthologous Groups. However, in the Pfam database [74], HP0894 belongs to the plasmid stabilization system protein family (PF05016). From the sequence homology search, we were able to get a hint of the function. However, a search for structural homologs with a Z score higher than 3.0 using the programs DALI showed that HP0894 is structurally similar to *Pyrococcus horikoshii* Archaeal RelE (PDB code: 1WMI, Z score = 7.8, pairwise RMSD = 2.8 Å), *E. coli* YoeB (PDB code: 2A6Q, Z score = 8.8, RMSD = 2.9 Å), and Guanyloribonuclease (PDB code: 1RGE, Z score = 3.3, pairwise RMSD = 3.4 Å). These proteins are both ribonucleases, have a similar number of residues as HP0894 (around 90), share a similar β-sheet topology with HP0894, and have a comparable location for two of their helices (Figure 4). As expected, they have no detectable sequence homology with HP0894 in PSI-BLAST searches and Blast2 (pairwise comparison) analyses. The structural homology search revealed HP0894 may have potential ribonuclease activity and represents the toxin-antitoxin (TA) system like RelE [75]. Generally, in a TA system, toxin expression induces arrest of cell growth, whereas the antitoxin neutralizes the toxin by a direct protein-protein interaction [76]. Both proteins of the toxin-antitoxin system are encoded within a single operon, with the toxin gene usually located directly downstream of the antitoxin gene [77]. Thus, we hypothesized: (i) HP0894 is a toxin molecule in *H. pylori*; (ii) there should be an antitoxin molecule that interacts with HP0894; and (iii) it should be near the gene location for *hp0894* on the chromosome, if an antitoxin molecule exists. Actually, we found that HP0895 (hypothetical protein) is an antitoxin molecule [78] locating upstream of the *hp0894* gene.

Our experimental data [78] showed that HP0894 and HP0895 forms a stable complex as a large multimer (hexamer, ((HP0895)^6^, (HP0894–HP0895)^6^), and the inhibitory effect of HP0894 on *E. coli* cell growth was neutralized by HP0895. In bacteria, toxins function, or are supposed to function, by inhibiting translation through mRNA cleavage [79]. With a RNA retardation experiment, the *in vitro* RNase activity of HP0894 was confirmed and HP0895 inhibited this RNase activity [78]. A primer extension experiment showed that HP0894-mediated mRNA cleavage occurred predominantly before adenine (A) or guanine (G) residues and we suggested -U:A- and -C:A- sequences are the most preferred cleavage sites [78]. The binding mode between HP0894 and HP0895 was more deeply studied using NMR and CD spectroscopy and we showed the binding interface of HP0894 [78]. Interestingly, HP0316 (hypothetical protein) that has an 85% sequence identity with HP0895 except for 30 residues at the *C*-terminal tail did not bind to HP0894, suggesting the *C*-terminal non-conserved tail of HP0895 may be responsible for binding of HP0894 [78]. Actually, with the synthesized *C*-terminal peptide of HP0895, the residue-specific interaction sites of HP0894 were cleared (Figure 4). These results indicate that the HP0894–HP0895 TA system, especially through negative regulation of the HP0894 toxin by the HP0895 antitoxin, may be related to the status of infections of *H. pylori* in the human gastric mucosa and to its survival in that locus.

**Figure 5:**
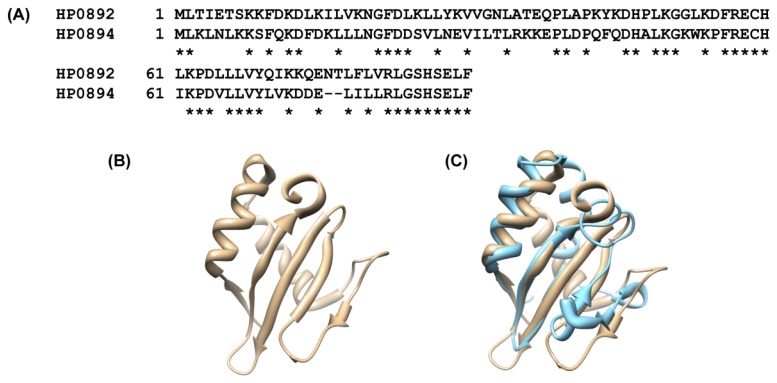
Comparison between HP0892 and HP0894. (**A**) Sequence homology between HP0892 and HP0894. Stars represent identical residues (53.3% identity in 90 residues); (**B**) Ribbon drawing of the representative conformer of HP0892 (PDB ID:2OTR); (**C**) Superposition of HP0892 (tan) and HP0894 (sky blue). The pairwise RMSD between two proteins was 0.712 Å. The topology of the two molecules is slightly different, especially in the loop regions.

Notably, HP0892 (hypothetical protein) and HP0894 share high sequence similarity (identity 53%). It is expected that HP0892 may be a paralog of HP0894. As a result, the structure of HP0892 is very similar to that of HP0894 [80] (Figure 5), and HP0892 is structurally similar to *Archaeal* RelE (aRelE) (Z score = 8.1, RMSD = 2.7 Å) and the YoeB toxin of *E. coli* (Z score = 9.6, RMSD = 2.9 Å) like HP0894. Based on the above study, HP0892 was speculated to be another toxin molecule. However, there is no comparable protein to the HP0895 antitoxin near the upstream or downstream of *hp0892* gene. Thus, the function of HP0892 is still questionable, which implies that most structural homologues do not reveal the function of unknown proteins. According to gene comparison studies using DNA microarrays [81], the *hp0892* gene is one of several *H. pylori* genes absent from a set of five cag pathogenicity island (PAI)-negative strains, while the *hp0894* gene is not. This may represent a marker for the identification of virulent strains or may represent novel virulence factors. Therefore, it is probable that the biological role of HP0892 is different from that of HP0894, aRelE, and YoeB, despite the sequence and/or structural similarities among them.

### 4.2. HP0315: Virulence-Associated Factor, Endoribonuclease

Virulence-associated protein, a product of the *vap* gene in various organisms, may be insufficient in itself, but is a requisite for virulence. The *vap* genes are known as factors or enzyme-producing factors that regulate the expression of true virulence genes or activate virulence factors by translational modification, processing of secretions or that are required for the activity of true virulence factors. Several *vap* genes (*vapA*, *B*, *C*, *D*, *H* and *I*) are known to exist in various organisms [82–84] but how the products of the *vap* genes are related to virulence remains unclear. *H. pylori* strain 26695 has only one type of virulence-associated protein, VapD. Two genes in this strain (HP0315 and HP0967) belong to *vapD* [85]. The exact biological role of the VapD protein has not yet been established, but several suggestions such as toxin, acid tolerance, plasmid stability, *etc*. have been made [86–88]. Here, we summarized the elucidation of the probable function of HP0315 with structural and biochemical studies.

**Figure 6:**
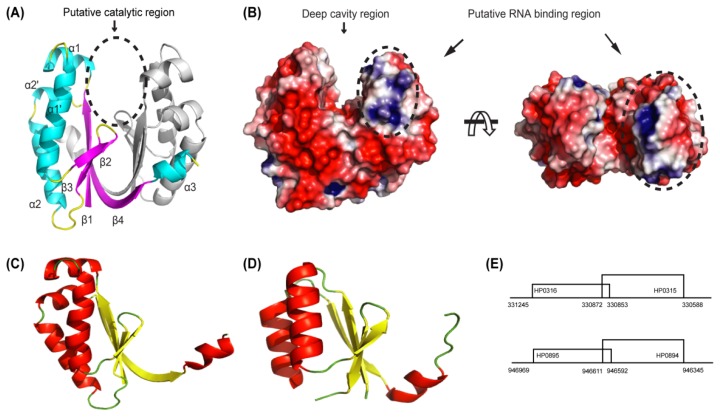
Structure of HP0315 from *H. pylori*. (**A**) Cartoon representation of the dimer of HP0315 (α-helices, β-strands and loops are cyan, magenta and yellow, respectively). Dotted circle represents the putative catalytic region located at the deep cavity region. (**B**) Surface representation of HP0315 showing positive and negative electrostatic potential in blue and red, respectively. The dotted circle represents the putative RNA-binding region. This region would be related to initial binding with RNA, and then a second catalytic reaction would occur around the deep cavity region. (**C**,**D**) Structural comparison between HP0315 (**C**); and the homologue, SSO1404 (PDB code:2ivy) (**D**). β-Strands are colored in “yellow” and α-helices in “red”. Both of the structures possess a ferredoxin-like fold. (**E**) Diagrams of the *hp0315* (*hp0894*) and *hp0316* (*hp0895*) encoding region from the chromosome of *H. pylori*.

The structure of HP0315 consists of 10 secondary structure elements: β1 (residues 1–8), α1 (residues 10–17), α1′ (residues 21–35), β2 (residues 38–41), β3 (residues 44–47), α2 (residues 53–66), α2′ (residues 68–73), β4 (residues 75–87) and α3 (residues 88–93). The monomer has a ferredoxin-like fold. It has the β1-(α1-α1′)-β2-β3-(α2-α2′)-β4-α3 instead of the β-α-β-β-α-β structure of the ferredoxin fold. The dimer of HP0315 is butterfly-shaped (PDB code: 3UI3, Figure 6). The β4 strand and the α3 helix associate with the adjacent monomer, forming a dimerization interface [89]. This structure is the first structure of a VapD family to our knowledge. A sequence homology search revealed that HP0315 is related to the CRISPR-associated protein Cas2, a novel family of endoribonucleases, suggesting the potential ribonuclease activity of HP0315. The structure-based alignment also yielded a high score from DALI for one of the Cas2 proteins, SSO1404 (PDB code: 2IVY) although the top-scoring proteins were mainly hypothetical unknown proteins. In addition, the interrelationships between VapD and Cas2 proteins were supported by a genomic analysis [90].

The sequence analysis yielded another interesting result: the two genes *HP0315* and *HP0316* exist as an operon, which is a functional unit of genomic DNA containing partially overlapping genes under the control of a single regulatory signal or promoter (gene coordinates: *HP0315* 330872–330588, *HP0316* 331245–330853, Figure 6). As described above, HP0316 has a sequence similarity of 88.9% with HP0895 [78], which might suggest the HP0315–HP0316 system is identical with the HP0894–HP0895 system. In other words, HP0315 might act as a toxin molecule like HP0894 although no sequence and structural similarity exists between them. However, HP0315 did not bind HP0316 and did not affect the cell viability in *in vivo* toxicity experiments [89]. From the sequence/structure analysis and biochemical experiments, HP0315 was speculated to be a ribonuclease but not a toxin even though the gene arrangement is similar to that of a TA system [89]. The RNase activity of HP0315 was confirmed by primer extension and gel retardation experiments, revealing purine-specific endoribonuclease activity [89].

Conclusively, HP0315, a member of the VapD family, has a structural similarity with the Cas2 family and has a gene arrangement similar to the TA system; however, it does not belong to any of them, like an evolutionary intermediate. The exact function of HP0315 has not been determined yet. However, considering the relationship with Cas2 and a TA system, as well as the endoribonuclease activity, HP0315 may be related to either cell maintenance or a defense mechanism against invasion, or possibly both such as Cas2 and/or a TA system.

### 4.3. Others: HP0062, HP0495, HP0827, HP1242, HP1423

**Figure 7:**
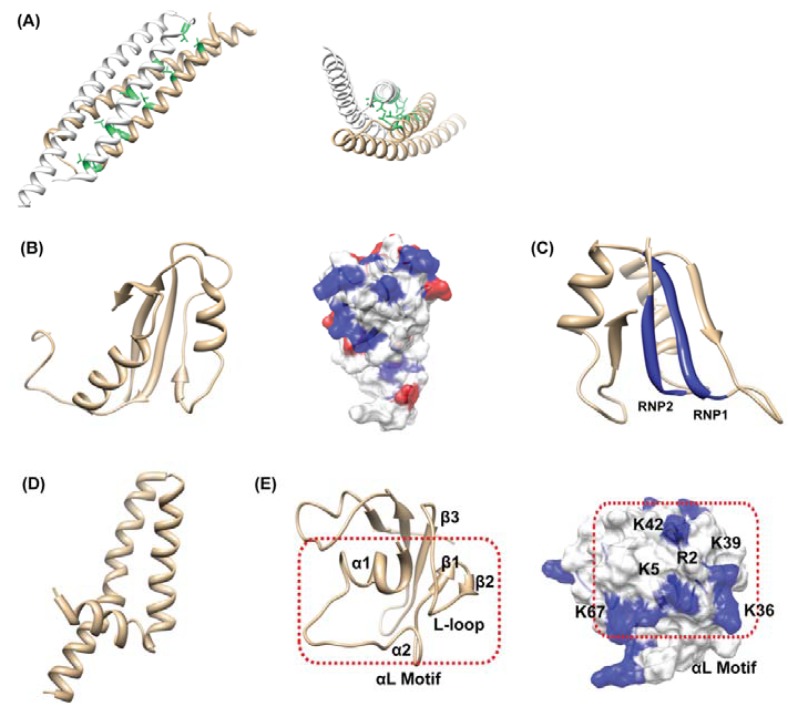
(**A**) Ribbon diagram of the HP0062 dimer is shown. Side and top views of the HP0062, showing the leucine zipper (green); (**B**) Ribbon drawing of the representative conformer of HP0495. Distribution of the surface charges on two distinct faces of HP0495 is shown. Positively-charged residues are blue, negatively-charged residues are red; (**C**) Ribbon drawing of the representative conformer of HP0827. Blue colors represent conserved RNP motifs lying side by side; (**D**) Ribbon drawing of the representative conformer of HP1242; (**E**) Ribbon drawing of the representative conformer of HP1423. The αL motif consists of two α-helices and the loop between β2 and β3. Electrostatic potential surface diagrams of HP1423 shows a strong concentration of positive charge in the proposed RNA-binding αL motif facing outwards.

The 3D structure of hypothetical protein HP0062 (PDB code: 3FX7) at 1.65 Å resolution was solved [91]. HP0062 is a small protein composed of 86 amino acids but it exists as dimer. The HP0062 monomer folds into a hairpin structure, in which two α-helices (the *N*- and *C*-helix) are connected by a short loop (Figure 7A) and the *N*-helix displays a modified leucine zipper. The protomers dimerize in an antiparallel arrangement, in which the *N* and *C* helices of one protomer pack against the *N* and *C* helices of the second protomer, forming a four-helix bundle. The two protomers in an asymmetric unit of the orthorhombic crystal are similar, and the topologically equivalent Ca carbons superimpose with a RMSD of 0.79 Å. Actually, the structure of HP0062 was also solved by another group but they reported the protein is monomeric (unpublished, PDB code: 2GTS). Since our gel filtration chromatography revealed the dimeric state of HP0062, it is believed that the biologically relevant form is a dimer [91]. The structural comparison indicated HP0062 has similarity with the coiled-coil segments of over 100 functionally unrelated proteins that are involved in various protein-protein interactions. Thus, the function of HP0062 is hard to directly estimate from the structural information. Interestingly, HP0062 shows extensively similar characteristics to those of the ESAT-6 family of Gram-positive bacteria; small dimer, helix-hairpin-helix structure, no signal peptide but with WXG motif in the hairpin bend (WRD in HP0062), and gene clusters with a protein with FtsK/SpoIIIE domain [92]. On the other hand, HP0062 also has similar characteristics to those of the TTS (Type Three Secretion) chaperones of Gram-negative bacteria; small dimer, an acidic pI, an overall α-helical character and a carboxy-terminal amphipathic α-helix [93]. These results might give a hint for the function of HP0062 as a transport chaperone and/or adaptor protein to facilitate interactions with host receptor proteins.

HP0495 is an 86-residue hypothetical protein with a molecular weight of 10,192.7 Da. The atomic coordinates of the final structure have been deposited in PDB (2H9Z). HP0495 has two α-helices and four β-strand, forming a ferredoxin-like fold, β1-α1-β2-β3-α2-β4 (Figure 7B). HP0495 is a completely unknown protein since HP0495 has a restricted sequence homology with unknown proteins from several bacteria [94,95]. The ubiquitous ones like HP0495 merit the highest priority for functional characterization because they have the greatest potential payoff in new biological knowledge. In this case, the structure of HP0495 and structural homology data may be more important and provide a clue for the function. Unfortunately, a structural homology search using DALI indicated that HP0495 has structural homology with a variety of proteins [94]. This should be because the ferredoxin-like fold of HP0495 is abundant in other structures. Twenty proteins had a higher Z-score of 5.0 from DALI analysis including the NikR protein from *Pyrococcus horikoshii* (nickel responsive repressor; PDB code: 2BJ9, RMSD = 2.9 Å), LrpA from *Thermus thermophilus* (transcriptional regulator; PDB code: 1RIS, RMSD = 2.9 Å), S6 protein from *Archaeoglobus fulgidus* (ribosomal protein; PDB code: 1Y7P, RMSD = 2.9 Å), and a hypothetical YbeD protein from *E. coli* (unknown; PDB code: 1RWU, RMSD = 3.6 Å). The structural comparison did not show a clear result. However, the function of HP0495 seems to be related to nucleic acid interaction since its homologues are mainly nucleic acid binding proteins and HP0495 possesses positive surface charges (Figure 7B).

HP0827 is classified as a putative single-stranded (ss)-DNA binding protein 12RNP2 precursor protein. The solution structure of HP0827 (PDB code: 2KI2) has a ferredoxin-like fold, β1-α1-β2-β3-α2-β4 [96]. The four β-strands are arranged in a right-handed twist and form an antiparallel β-sheet that packs against the two α-helices (Figure 7C). This protein contains one RRM (RNA Recognition Motif) comprised of two ribonucleo-protein motifs (RNP1, Lys/Arg-Gly-Phe/Tyr-Gly/Ala-Phe/Tyr-Val/Ile/ Leu-X-Phe/Tyr and RNP2, Ile/Val/Leu-Phe/Tyr–Ile/Val/Leu-X-Asn-Leu). Since the RRM motif is an abundant component in protein structures, only the RRM motif could not tell the exact function of HP0062. Actually, a total of 6,056 RRM motifs can be found in 3541 different proteins in the Pfam database [97]. We could not elucidate the biological function of HP0827 from a structural basis, though the structure may provide information on the putative RNA binding site. Further biological studies may be required for this case.

The HP1242 gene encodes a 76-residue conserved hypothetical protein with a molecular weight of 9111 Da. HP1242 adopts a full helical structure, which is composed of three α-helices [98]. These correspond to residues 6–14 (αI), 18–38 (αII), and 43–75 (αIII). The overall structure of HP1242 represents a coiled-coil-like conformation (Figure 7D). Based on the sequence homology, HP1242 is classified as the DUF (Domain of Unknown Function) 465 family, which has an unknown function. These family members are found in several bacterial proteins, and also in the heavy chain of eukaryotic myosin and kinesin, which are predicted to form coiled coil structures. HP1242 has a structural homology with a variety of proteins including the rop protein (transcription regulation), arfaptin 2 fragment (signaling protein), sensory rhodopsin II fragment (membrane protein complex) and so on [99]. This result indicates that the function of HP1242 could not be evaluated by only a structural comparison.

We also determined the solution structure of HP1423, which has 84 amino acid residues. HP1423 is a hypothetical protein as well. According to the Pfam database, HP1423 belongs to S4 (PF01479) superfamily. The S4 domain is a small domain consisting of 60–65 amino acid residues that probably mediates binding to RNA [100]. The structure of HP1423 is composed of five β-strands and three α-helices [101]. The topology can be described as α1-α2-β2-β1-β3-β4-α3-β5 (Figure 7E). Notably, the region, extending from α1 through β3, forms an obvious structural motif, the so called αL motif, because of the two α-helices and the loop between β2 and β3 which forms an L-shaped meander (Figure 7E). This structural motif shows a high degree of conservation between different families within the S4 (PF01479) superfamily and may be important for interaction with RNA [100]. The surface region of the αL motif of HP1423 has a strong concentration of positive charge and the loop between β4 and α3 exposes another positively charged side chain of K67, which may raise the possibility that HP1423 is a RNA binding protein (Figure 7E). The DALI result also showed that HP1423 is structurally similar to proteins that belong to S4 superfamily. The S4 superfamily includes the Hsp15 protein (PDB code: 1DM9-B), ribosomal small subunit pseudouridine synthase A (PDB code: 1VIO-A), 30S ribosomal protein S4 (PDB code: 1FJG-D), and so on. All these homologues contain the αL motif. However, the distribution of positively-charged residues on the protein surfaces was somewhat different between homologous proteins [101], suggesting that HP1423 may bind to RNA through the αL motif in a similar but not exactly same manner as the S4 RNA binding proteins.

## 5. Different Characteristic with Known Function

Bioinformatics tools have been remarkably developed, providing biologists valuable information for functional elucidation. Nevertheless, prediction of protein function from sequence and structure is a difficult problem, because homologous proteins often have different functions. In addition, the protein that is classified into a known protein, based on the sequence homology, often shows some functional ambiguity since the composition of the operon is quite different from that of the known system. In addition, some of the proteins, which are considered to be well characterized, may have additional functions beyond their listed function [102]. In this regard, it is still worth investigating known proteins from a newly sequenced genome for their cell and biological functions. Here, we present two examples of well-defined proteins that have different characteristics compared to the homologues.

**Figure 8:**
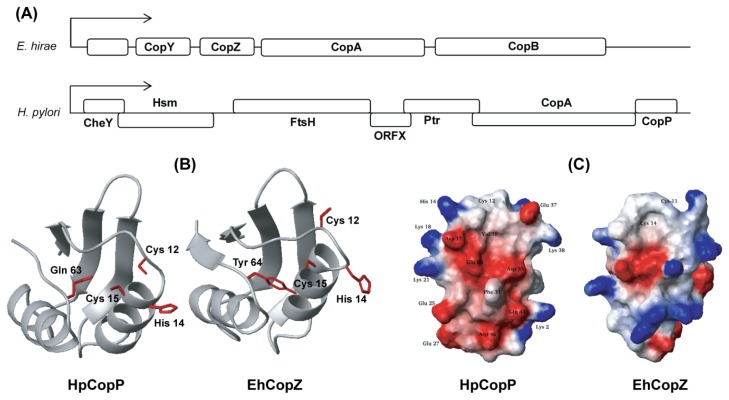
Structural comparison between apo-HpCopP and apo-CopZ. (**A**) The composition of cop ORFs of *H. pylori* and *E. hirae*; (**B**) The orientation of the two cysteines and one histidine in the CXXC motif of HpCopP is compared with that of EhCopZ. The hydrophobic protection by Tyr 64 in loop V stabilizes the Cu(I)-coordination in EhCopZ. This residue is highly conserved in bacterial proteins, but is replaced with Gln 63 in HpCopP. The side-chain of Gln 63 is not fully exposed to the solvent and points toward the metal binding site in apo-HpCopP. The structures of EhCopZ (PDB ID: 1CPZ) were obtained from the PDB; (**C**) The electrostatic potential surfaces of HpCopP and EhCopZ are compared to each other. The positively and negatively charged residues are represented in blue and red, respectively.

Copper metabolism by copper chaperones has been studied extensively in both eukaryotes and bacteria. In the gram-positive bacterium, *Enterococcus hirae*, the cop operon is composed of four proteins: two integral membrane P-type ATPases, CopA, and CopB which transport Cu(I) into cells under Cu(I) limiting conditions and eliminate Cu(I) under conditions of high Cu(I) levels, respectively [103,104]. The imported copper ions are transferred from CopA to the CopZ chaperone [105–107] and CopY, a gene repressor, is released from the cop operon promoter when Cu(I) is delivered to CopY by the copper chaperone, CopZ (Figure 8A). In the case of the gram-negative bacterium, *H. pylori*, copper homeostasis seems to be maintained by only two proteins CopA and CopP (HP1073). The *H. pylori* cop operon (Figure 8A) is included in a novel stress-responsive operon (sro), which encodes the flagellar motor switch protein CheY, the putative methyltransferase Hsm, the cell division protein FtsH, the putative phosphatidyltransferase Ptr, the heavy metal-binding proteins CopA and CopP, and an open reading frame of unknown function [108]. CopA is a member of the bacterial copper ion ATPase family, and CopP, which is homologous to *E. hirae* CopZ, is a putative copper binding regulatory protein of 66 amino acids [104,108]. CopA of *H. pylori* was identified as a Cu(II) export ATPase [109], which shows that its biological role is more similar to that of *E. hirae* CopB, rather than CopA [110]. Moreover, the CopP gene resides immediately downstream of the CopA gene, while the *E. hirae* CopZ gene resides upstream of the CopA gene. Therefore, the cop operon organization seems to be evolutionarily modified in each bacterium.

Generally, CopZ proteins share a conserved structure, βαββαβ with a similar metal binding region. Interestingly, HpCopP adopts the βαββα fold with a missing *C*-terminal β strand [111]. The overall topologies of the secondary structural components are very similar between the CopZs and HpCopP, while some variations in the loop regions appear (Figure 8). The relationship between the unusual fold and the copper specificity was evaluated [111]. We showed that HpCopP was not adequate for Cu(II) binding since the fold stability decreased in the presence of Cu(II) ion, suggesting that the structure of HpCopP is optimized for the transfer of toxic Cu(I). The absence of the *C*-terminal β-strand may lead to decreased conformational stability of loop I including the CXXC motif (Cu binding motif), which probably contributes to the disulfide bond formation between the two cysteine residues in the presence of Cu(II) ion. These findings should be helpful in evaluating the copper metabolism related with HpCopA and HpCopP in *H. pylori.*

Acyl carrier protein (ACP) found in bacteria is a monofunctional protein, that is, a type II enzyme in fatty acid biosynthesis. All the ACPs are decorated by acyl carrier protein synthase (ACPS) with fatty acids, which are covalently attached as thioesters to the 4′-phosphopantetheine prosthetic group at highly conserved Ser 36 [112]. Fatty acid binding has little influence on ACP conformation under physiological conditions [113], but it stabilizes ACP against denaturation at alkaline pH [114].

**Figure 9:**
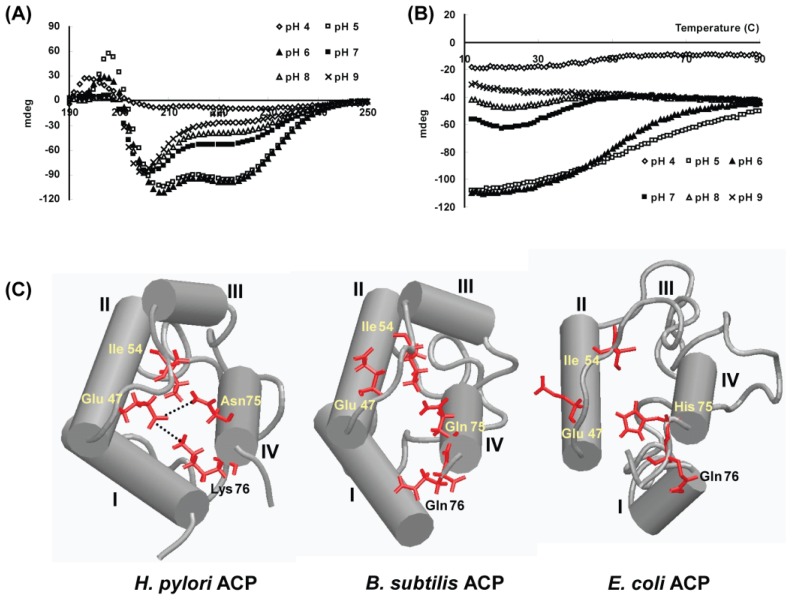
Comparison of the *H. pylori* ACP structure with the *B. subtilis* ACP and *E. coli* ACP structures. (**A**) CD spectra of HpACP recorded at various pHs. At neutral and alkaline pH, the conformational transition of HpACP occurred; (**B**) Tm curves of HpACP. At acidic pH 6, the temperature curves of the HpACP showed a distinct melting temperature around 50 °C. The unfolding process above neutral pH proceeded through multi-phasic changes, showing at least three stages exist; (**C**) Schematic representation showing the buried hydrophilic residues Glu 47, Asn 75 and Lys 76 in the energy minimized average structure of HpACP. Putative hydrogen-bonding interactions are indicated by dotted lines. The corresponding residues are compared to those of *B. subtilis* ACP and *E. coli* ACP.

*H. pylori* ACP (HP0559) is composed of 78 amino acids with a pI value of 3.9, and its primary structure is similar with those of homologous ACPs. Like other ACPs, HpACP forms a helical bundle structure through hydrophobic contacts between the helices (Figure 9). However, we found an unusual behavior of HpACP at neutral pH [115]. HpACP exists as a partially unfolded state at neutral pH, which is a unique characteristic of HpACP (Figure 9). In contrast, the overall helical structure of *E. coli* ACP was maintained at pH 7 [116] and *Vibrio harveyi* ACP exhibited a random coil-like conformation at pH 7 [117].

The pH dependent-conformational change of a protein from *H. pylori* is a very interesting feature, considering that the environment of the stomach has a low pH. A few studies showed the relationship between the mutation of various residues and the pH-dependent structural stability. The mutation of Val 43 to Ile in *E. coli* ACP increases the stability to pH-induced expansion in electrophoretic systems, concomitantly inducing more compact folding [118]. The mutants F50 A and I54 A of *V. harveyi* are incapable of adopting a native conformation with increased hydrodynamic radius at neutral pH [117]. In addition, a few basic residues scattered near the *N*- and *C*-termini, for example, His 75 of *E. coli* ACP, are necessary for ACP to maintain a native conformation at neutral pH [119]. Through our structural analysis, we found that several hydrophilic residues (Glu 47, Asn 75, and Lys 76) play an important role in structural stability. Therefore, we could suggest that, unlike other ACPs, the helical bundle of *H. pylori* ACP is maintained by, not only hydrophobic interactions, but also by hydrophilic interactions and these interactions may be weakened by elevation of the pH because the exchange rate of protons attached to the side chain amide of Asn and Lys may increase [115].

## 6. Concluding Remarks

Mass genomic sequencing has been yielding many protein sequences that cannot be annotated, and structural genomics projects are yielding many protein structures that have unknown functions. Unknown proteins represent up to about half of the proteins in prokaryotic genomes, and much more than this in higher plants and animals [120]. In bacteria such as *H. pylori*, 30–40% of the proteins encoded by typical bacterial genomes have no clear known function [121]. Thus, a major issue of genomic studies may be to narrow the gap between the richness of sequences (and/or structures) and functional characterization as subsequent experimental investigation is costly and time-consuming [122]. Actually, only 54% of *E. coli* gene products have been experimentally investigated so far [123]. Therefore, more robust bioinformatic methods or approaches may be necessary to overcome this situation. Here, we showed several examples of successful cases for elucidating the function of *H. pylori* unknown proteins based on their structural information, which supports the potential of structural comparison for functional identification. It is hoped that the structural comparison can at least act as a guide to the possible function, even though all structures cannot elucidate the actual function.

## Supplementary Information



## Figures and Tables

**Table 1:** Genomes of *H. pylori*. Currently, 36 sub-species have been identified and the genome sizes are from 1.55 mega base pairs to 1.82 mega base pairs. All data were collected and processed from the NCBI genome database [17].

Organism	Gene	Size (Mb)	GC%	Protein (unknown)	Type	Project
*Helicobacter pylori*	1480	1.57	38.9	1405 (476)	chr a	Gyeongsang National University College of Medicine and 21c Frontier Human Genome Functional Research Project *Helicobacter pylori* 52 genome sequencing project

*Helicobacter pylori* 2017	1647	1.55	39.3	1593 (525)	chr	Pathogen Biology Laboratory, University of Hyderabad *Helicobacter pylori* 2017 genome sequencing project

*Helicobacter pylori* 2018	1655	1.56	39.3	1603 (459)	chr	Pathogen Biology Laboratory, University of Hyderabad *Helicobacter pylori* 2018 genome sequencing project

*Helicobacter pylori* 26695	1627	1.67	38.9	1573 (1301)	chr	TIGR (The Institute for Genome Research) Causes gastric inflammation and peptic ulcer disease

*Helicobacter pylori* 35A	1560	1.57	38.9	1470 (362)	chr	Baylor College of Medicine Reference genome for the Human Microbiome Project

*Helicobacter pylori* 51	1495	1.59	38.8	1415 (386)	chr	Gyeongsang National University College of Medicine and 21c Frontier Human Functional Genome Research Project Bacterium isolated from duodenal ulcer patient

*Helicobacter pylori* 83	1656	1.62	38.7	1609 (445)	chr	Baylor College of Medicine Reference genome for the Human Microbiome Project

*Helicobacter pylori* 908	1646	1.55	39.3	1595 (444)	chr	University of Hyderabad, India *Helicobacter pylori* 908 genome sequencing project

*Helicobacter pylori* B38	1571	1.58	39.2	1382 (643)	chr	Institut Pasteur Causes peptic ulcers

*Helicobacter pylori* B8	1744	1.67	38.8	1702 (736)	chr	CeBitec, Bielefeld University *Helicobacter pylori* B8 genome sequencing project
	5	0.01	35.9	5 (3)	plsm b	

*Helicobacter pylori* Cuz20	1606	1.64	38.9	1564 (538)	chr	Dept. of Molec. Microbiology, Washington University Medical School, Saint Louis *Helicobacter pylori* Cuz20 genome sequencing project

*Helicobacter pylori* F16	1543	1.58	38.9	1500 (494)	chr	The University of Tokyo *Helicobacter pylori* F16 genome sequencing project

*Helicobacter pylori* F30	1522	1.57	38.8	1479 (470)	chr	The University of Tokyo *Helicobacter pylori* F30 genome sequencing project.
	5	0.01	34.1	5 (1)	plsm	

*Helicobacter pylori* F32	1533	1.58	38.9	1490 (485)	chr	The University of Tokyo *Helicobacter pylori* F32 genome sequencing project.
	1	0	36.7	1 (0)	plsm	

*Helicobacter pylori* F57	1563	1.61	38.7	1520 (498)	chr	The University of Tokyo *Helicobacter pylori* F57 genome sequencing project.

*Helicobacter pylori* G27	1570	1.65	38.9	1493 (470)	chr	University of Oregon Strain used extensively in *H. pylori* research
	11	0.01	34.9	11 (5)	plsm	

*Helicobacter pylori* Gambia94/24	1646	1.71	39.1	1604 (611)	chr	Berg lab, Washington University Medical School *Helicobacter pylori* Gambia94/24 genome sequencing project
	1	0	37.4	1 (1)	plsm	

*Helicobacter pylori* HPAG1	1573	1.60	39.1	1531 (515)	chr	Washington University (WashU) Isolated from a Swedish patient with chronic atrophic gastritis
	8	0.01	36.4	8 (5)	plsm	

*Helicobacter pylori* India7	1638	1.68	38.9	1600 (561)	chr	Berg lab, Washington University Medical School *Helicobacter pylori* Ind7 genome sequencing project

*Helicobacter pylori* J99	1534	1.64	39.2	1488 (560)	chr	Astrazeneca-Boston Causes gastric inflammation and peptic ulcer disease

*Helicobacter pylori* Lithuania75	1588	1.62	38.8	1546 (522)	chr	Berg lab, Washington University Medical School *Helicobacter pylori* Lit75 genome sequencing project
	19	0.02	33.7	19 (12)	plsm	

*Helicobacter pylori* P12	1624	1.67	38.8	1568 (450)	chr	Max von Pettenkofer-Institut für Hygiene und Medizinische Mikrobiologie, Ludwig-Maximilians-Universität München Clinical isolate
	10	0.01	35.1	10 (2)	plsm	

*Helicobacter pylori* PeCan4	1597	1.63	38.9	1555 (529)	chr	Dept. of Molec. Microbiology, Washington University Medical School, Saint Louis *Helicobacter pylori* PeCan4 genome sequencing project
	8	0.01	32.9	8 (0)	plsm	

*Helicobacter pylori* Puno120	1567	1.62	38.9	1525 (518)	chr	Washington University Medical School *Helicobacter pylori* Puno120 genome sequencing
	15	0.01	35.8	15 (13)	plsm	

*Helicobacter pylori* Puno135	1615	1.65	38.8	1573 (532)	chr	Washington University Medical School Genome sequence of *Helicobacter pylori* strain Puno135

*Helicobacter pylori* SJM180	1623	1.66	38.9	1581 (558)	chr	Dept. of Molec. Microbiology, Washington University Medical School, Saint Louis *Helicobacter pylori* SJM180 genome sequencing project

*Helicobacter pylori* SNT49	1557	1.61	39	1515 (495)	phage	Washington University Medical School Genome sequence of *Helicobacter pylori* SNT49
	4	0	37.4	4 (3)	plsm	

*Helicobacter pylori* Sat464	1544	1.56	39.1	1502 (504)	chr	Dept. Molec. Microbiology, Washintgton University Medical School in Saint Louis *Helicobacter pylori* Sat464 genome sequencing project.
	6	0.01	33.5	6 (4)	plsm	

*Helicobacter pylori* Shi470	1647	1.61	38.9	1568 (593)	chr	Washington University Medical School Clinical isolate from the Amazon River region

*Helicobacter pylori* SouthAfrica7	1585	1.65	38.4	1543 (555)	chr	Berg lab, Washington University Medical Shool *Helicobacter pylori* SouthAfrica7 genome sequencing project
	29	0.03	33.7	29 (19)	plsm	

*Helicobacter pylori* v225d	1625	1.59	39	1541 (555)	chr	The Pathosystems Resource Integration Center (PATRIC) *Helicobacter pylori* v225 genome sequencing
	9	0.01	32.9	9 (7)	plsm	

*Helicobacter pylori* B45	27	0.02	37.3	27 (26)	chr S/C c	Karolinska Institute *Helicobacter pylori* B45 genome sequencing project

*Helicobacter pylori* 98-10	1566	1.57	38.8	1527 (1527)	S/C	Vanderbilt University School of Medicine Gastric cancer strain

*Helicobacter pylori* B128	1770	1.65	38.8	1731 (1731)	S/C	Vanderbilt University School of Medicine Gastric ulcer strain

*Helicobacter pylori* HPKX_438_AG0C 1	2939	1.82	39.5	2898 (1564)	S/C	Washington University Medical School Clinical isolate

*Helicobacter pylori* HPKX_438_CA4C1	3962	1.57	39.2	3925 (1548)	S/C	Washington University Medical School Isolate from a patient with gastric carcinoma

Total	**59,776**	**-**	**-**	**57,872 (23,261)**	-	-

a Chromosome;

b Plasmid;

c S/C: Scaffolds or Contigs.

**Table 2:** Unknown protein structures from *H. pylori.* A total of 28 unknown protein structures were elucidated using X-ray diffraction and NMR method. All data were collected and processed from PDB database [52].

PDB ID	Chain		Structure	Macromolecule Name	Classification	Scop Fold	Exp. Method
	ID	AA	MW				
1MW7	A	240	27161.20	Hypothetical protein HP0162	SG a, unknown function	YebC-like	X-ray
1S2X	A	206	23998.70	Cag-Z	Unknown function	STAT-like	X-ray
1Z8M	A	88	10394.30	Conserved hypothetical protein HP0894	SG, unknown function	RelE-like	NMR
1ZHC	A	76	9130.38	hypothetical protein HP1242	Unknown function		NMR
1ZKE	A, B, C, D, E, F	83	56798.00	Hypothetical protein HP1531	SG, unknown function	ROP-like	X-ray
2ATZ	A	180	22049.45	Predicted coding region HP0184	SG, unknown function	Prim-pol domain	X-ray
2BO3	A	94	11101.70	Hypothetical protein HP0242	SG, unknown function	HP0242-like	X-ray
2EVV	A, B, C, D	207	95692.83	hypothetical protein HP0218	SG, unknown function		X-ray
2F6S	A, B	201	47249.90	cell filamentation protein, putative	SG, unknown function	Fic-like	X-ray
2G3V	A, B, C, D	208	104975.36	CAG pathogenicity island protein 13	Unknown function		X-ray
2GTS	A	86	10626.50	hypothetical protein HP0062	SG, unknown function	Ferritin-like	X-ray
2H9Z	A	86	10205.80	Hypothetical protein HP0495	SG, unknown function	Ferredoxin-like	NMR
2I9I	A	254	29526.70	Hypothetical protein	SG, unknown function	Anticodon-binding domain-like	X-ray
2JOQ	A	91	10673.20	Hypothetical protein HP0495	SG, unknown function	Ferredoxin-like	NMR
2K0Z	A	110	12948.60	Uncharacterized protein HP1203	SG, unknown function		NMR
2K6P	A	92	10472.30	Uncharacterized protein HP1423	Unknown function		NMR
2OTR	A	98	11502.60	Hypothetical protein HP0892	SG, unknown function		NMR
2OUF	A	94	11148.60	Hypothetical protein	SG, unknown function		X-ray
2UVP	A, B, C, D	186	87079.82	HOBA, HP1230	Unknown function		X-ray
2XRH	A	100	11635.31	HP0721	Unknown function		X-ray
3BGH	A, B	236	55233.49	Putative neuraminyllactose-binding hemagglutinin homolog	SG, unknown function		X-ray
3CWX	A, B, C	176	62332.80	protein CagD	Unknown function		X-ray
3CWY	A	176	20841.15	protein CagD	Unknown function		X-ray
3F42	A, B	99	22671.87	protein HP0035	SG, unknown function		X-ray
3FX7	A, B	94	23207.80	Uncharacterized protein, HP0062	Unknown function		X-ray
3KWL	A	514	60116.00	Uncharacterized protein	Unknown function		X-ray
3MLG	A, B	189	43924.40	Uncharacterized protein	Unknown function		X-ray
3MLI	A, B, C, D	100	47758.96	Uncharacterized protein HP0242	Unknown function		X-ray

a Structural genomics.
