# Supplementary material for: Structural Analysis of Hypothetical Proteins from Helicobacter pylori: An Approach to Estimate Functions of Unknown or Hypothetical Proteins
Source: Int J Mol Sci. 2012 Jun 8;13(6):7109–37. doi: 10.3390/ijms13067109 (PMC3397514; doi:10.3390/ijms13067109)
Supplement: Supplementary file 1 [file ijms-13-07109-s001.pdf]

Structural Analysis of Hypothetical Proteins from *Helicobacter pylori*: An Approach to Estimate Functions of Unknown or Hypothetical Proteins

Supplementary Information

Table S1. The protein structures from *H. pylori* determined up to date.

PDB ID	Chain ID	AA	Structure MW	Chain MW	Ligand MW	Macromolecule Name	Classification	Ligand Name	Ligand ID	Macromol. Type	Exp. Method	Rel. Date	Center Name
**1E9Y**	A	238	88526.37	26571.90		Urease subunit alpha	Hydrolase			Protein	X-ray	2001-11-01	
	B	569	88526.37	61762.00	75.07	Urease subunit beta	Hydrolase	Acetohydroxamic acid	HAE	Protein	X-ray	2001-11-01	
	B	569	88526.37	61762.00	190.20	Urease subunit beta	Hydrolase	Lysine nz-carboxylic acid	KCX	Protein	X-ray	2001-11-01	
	B	569	88526.37	61762.00	58.70	Urease subunit beta	Hydrolase	Nickel (ii) ion	NI	Protein	X-ray	2001-11-01	
**1E9Z**	A	238	88435.30	26571.90		Urease subunit alpha	Hydrolase			Protein	X-ray	2001-11-01	
	B	569	88435.30	61746.00	190.20	Urease subunit beta	Hydrolase	Lysine nz-carboxylic acid	KCX	Protein	X-ray	2001-11-01	
	B	569	88435.30	61746.00	58.70	Urease subunit beta	Hydrolase	Nickel (ii) ion	NI	Protein	X-ray	2001-11-01	
**1FUE**	A	163	17761.55	17305.20	456.35	Flavodoxin	Electron transport	Flavin mononucleotide	FMN	Protein	X-ray	2002-02-06	
**1G6O**	A	330	77214.21	37914.60	427.20	Cag-alpha	Hydrolase	Adenosine-5'-diphosphate	ADP	Protein	X-ray	2001-01-24	MCSG [a]
	A	330	77214.21	37914.60	196.11	Cag-alpha	Hydrolase	Selenomethionine	MSE	Protein	X-ray	2001-01-24	MCSG
	B	330	77214.21	37914.60	427.20	Cag-alpha	Hydrolase	Adenosine-5'-diphosphate	ADP	Protein	X-ray	2001-01-24	MCSG
	B	330	77214.21	37914.60	196.11	Cag-alpha	Hydrolase	Selenomethionine	MSE	Protein	X-ray	2001-01-24	MCSG
	B	330	77214.21	37914.60	106.12	Cag-alpha	Hydrolase	Di(hydroxyethyl)ether	PEG	Protein	X-ray	2001-01-24	MCSG
**1J2Y**	A	167	18689.55	18499.40	190.15	3-dehydroquinate dehydratase	Lyase	1,3,4-trihydroxy-5-oxo-cyclohexanecarboxylic acid	DQA	Protein	X-ray	2003-06-24	
**1J2Z**	A	270	30547.34	29896.70	96.06	Acyl-[acyl-carrier-protein]--UDP-N-acetylglucosamine O-acyltransferase	Transferase	Sulfate ion	SO4	Protein	X-ray	2004-01-27	
	A	270	30547.34	29896.70	308.43	Acyl-[acyl-carrier-protein]--UDP-N-acetylglucosamine O-acyltransferase	Transferase	2-hydroxymethyl-6-octylsulfanyl-tetrahydro-pyran-3,4,5-triol	SOG	Protein	X-ray	2004-01-27	
	A	270	30547.34	29896.70	150.09	Acyl-[acyl-carrier-protein]--UDP-N-acetylglucosamine O-acyltransferase	Transferase	L(+)-tartaric acid	TLA	Protein	X-ray	2004-01-27	
**1J6X**	A	160	37837.97	18704.40	196.11	Autoinducer-2protein luxs	Signaling protein	Selenomethionine	MSE	Protein	X-ray	2001-06-08	
	A	160	37837.97	18704.40	65.38	Autoinducer-2protein luxs	Signaling protein	Zinc ion	ZN	Protein	X-ray	2001-06-08	

	B	160	37837.97	18704.40	196.11	Autoinducer-2protein luxs	Signaling protein	Selenomethionine	MSE	Protein	X-ray	2001-06-08
	B	160	37837.97	18704.40	65.38	Autoinducer-2protein luxs	Signaling protein	Zinc ion	ZN	Protein	X-ray	2001-06-08
**1JI4**	A	144	205615.45	16960.60	55.85	Neutrophil-activating protein a	Metal transport	Fe (iii) ion	FE	Protein	X-ray	2002-10-09
	A	144	205615.45	16960.60	118.18	Neutrophil-activating protein a	Metal transport	(4s)-2-methyl-2,4-pentanediol	MPD	Protein	X-ray	2002-10-09
	A	144	205615.45	16960.60		Neutrophil-activating protein a	Metal transport	Unknown atom or ion	UNX	Protein	X-ray	2002-10-09
	B	144	205615.45	16960.60	55.85	Neutrophil-activating protein a	Metal transport	Fe (iii) ion	FE	Protein	X-ray	2002-10-09
	B	144	205615.45	16960.60	118.18	Neutrophil-activating protein a	Metal transport	(4s)-2-methyl-2,4-pentanediol	MPD	Protein	X-ray	2002-10-09
	B	144	205615.45	16960.60		Neutrophil-activating protein a	Metal transport	Unknown atom or ion	UNX	Protein	X-ray	2002-10-09
	C	144	205615.45	16960.60	55.85	Neutrophil-activating protein a	Metal transport	Fe (iii) ion	FE	Protein	X-ray	2002-10-09
	C	144	205615.45	16960.60	118.18	Neutrophil-activating protein a	Metal transport	(4s)-2-methyl-2,4-pentanediol	MPD	Protein	X-ray	2002-10-09
	C	144	205615.45	16960.60		Neutrophil-activating protein a	Metal transport	Unknown atom or ion	UNX	Protein	X-ray	2002-10-09
	D	144	205615.45	16960.60	55.85	Neutrophil-activating protein a	Metal transport	Fe (iii) ion	FE	Protein	X-ray	2002-10-09
	D	144	205615.45	16960.60	118.18	Neutrophil-activating protein a	Metal transport	(4s)-2-methyl-2,4-pentanediol	MPD	Protein	X-ray	2002-10-09
	D	144	205615.45	16960.60		Neutrophil-activating protein a	Metal transport	Unknown atom or ion	UNX	Protein	X-ray	2002-10-09
	E	144	205615.45	16960.60	55.85	Neutrophil-activating protein a	Metal transport	Fe (iii) ion	FE	Protein	X-ray	2002-10-09
	E	144	205615.45	16960.60	118.18	Neutrophil-activating protein a	Metal transport	(4s)-2-methyl-2,4-pentanediol	MPD	Protein	X-ray	2002-10-09
	E	144	205615.45	16960.60		Neutrophil-activating protein a	Metal transport	Unknown atom or ion	UNX	Protein	X-ray	2002-10-09
	F	144	205615.45	16960.60	55.85	Neutrophil-activating protein a	Metal transport	Fe (iii) ion	FE	Protein	X-ray	2002-10-09
	F	144	205615.45	16960.60	118.18	Neutrophil-activating protein a	Metal transport	(4s)-2-methyl-2,4-pentanediol	MPD	Protein	X-ray	2002-10-09
	F	144	205615.45	16960.60		Neutrophil-activating protein a	Metal transport	Unknown atom or ion	UNX	Protein	X-ray	2002-10-09
	G	144	205615.45	16960.60	55.85	Neutrophil-activating protein a	Metal transport	Fe (iii) ion	FE	Protein	X-ray	2002-10-09
	G	144	205615.45	16960.60	118.18	Neutrophil-activating protein a	Metal transport	(4s)-2-methyl-2,4-pentanediol	MPD	Protein	X-ray	2002-10-09
	G	144	205615.45	16960.60		Neutrophil-activating protein a	Metal transport	Unknown atom or ion	UNX	Protein	X-ray	2002-10-09
	H	144	205615.45	16960.60	55.85	Neutrophil-activating protein a	Metal transport	Fe (iii) ion	FE	Protein	X-ray	2002-10-09
	H	144	205615.45	16960.60	118.18	Neutrophil-activating protein a	Metal transport	(4s)-2-methyl-2,4-pentanediol	MPD	Protein	X-ray	2002-10-09
	H	144	205615.45	16960.60		Neutrophil-activating protein a	Metal transport	Unknown atom or ion	UNX	Protein	X-ray	2002-10-09
	I	144	205615.45	16960.60	55.85	Neutrophil-activating protein a	Metal transport	Fe (iii) ion	FE	Protein	X-ray	2002-10-09
	I	144	205615.45	16960.60	118.18	Neutrophil-activating protein a	Metal transport	(4s)-2-methyl-2,4-pentanediol	MPD	Protein	X-ray	2002-10-09
	I	144	205615.45	16960.60		Neutrophil-activating protein a	Metal transport	Unknown atom or ion	UNX	Protein	X-ray	2002-10-09
	J	144	205615.45	16960.60	55.85	Neutrophil-activating protein a	Metal transport	Fe (iii) ion	FE	Protein	X-ray	2002-10-09
	J	144	205615.45	16960.60	118.18	Neutrophil-activating protein a	Metal transport	(4s)-2-methyl-2,4-pentanediol	MPD	Protein	X-ray	2002-10-09

	J	144	205615.45	16960.60		Neutrophil-activating protein a	Metal transport	Unknown atom or ion	UNX	Protein	X-ray	2002-10-09	
	K	144	205615.45	16960.60	55.85	Neutrophil-activating protein a	Metal transport	Fe (iii) ion	FE	Protein	X-ray	2002-10-09	
	K	144	205615.45	16960.60	118.18	Neutrophil-activating protein a	Metal transport	(4s)-2-methyl-2,4-pentanediol	MPD	Protein	X-ray	2002-10-09	
	K	144	205615.45	16960.60		Neutrophil-activating protein a	Metal transport	Unknown atom or ion	UNX	Protein	X-ray	2002-10-09	
	L	144	205615.45	16960.60	55.85	Neutrophil-activating protein a	Metal transport	Fe (iii) ion	FE	Protein	X-ray	2002-10-09	
	L	144	205615.45	16960.60	118.18	Neutrophil-activating protein a	Metal transport	(4s)-2-methyl-2,4-pentanediol	MPD	Protein	X-ray	2002-10-09	
	L	144	205615.45	16960.60		Neutrophil-activating protein a	Metal transport	Unknown atom or ion	UNX	Protein	X-ray	2002-10-09	
**1KLX**	A	138	15367.80	15367.80		Cysteineprotein B	Hydrolase			Protein	X-ray	2002-09-11	
**1MW7**	A	240	27161.20	27161.20		Hypothetical protein hp0162	SG[b], unknown function			Protein	X-ray	2003-01-28	NSGC [c]
**1NLY**	A	330	77410.55	37914.60	523.24	Virb11 homolog	Hydrolase	Phosphothiophosphoric acid-adenylate ester	ATG	Protein	X-ray	2003-05-06	
	A	330	77410.55	37914.60	24.31	Virb11 homolog	Hydrolase	Magnesium ion	MG	Protein	X-ray	2003-05-06	
	A	330	77410.55	37914.60	196.11	Virb11 homolog	Hydrolase	Selenomethionine	MSE	Protein	X-ray	2003-05-06	
	B	330	77410.55	37914.60	414.49	Virb11 homolog	Hydrolase	Nonaethylene glycol	2PE	Protein	X-ray	2003-05-06	
	B	330	77410.55	37914.60	523.24	Virb11 homolog	Hydrolase	Phosphothiophosphoric acid-adenylate ester	ATG	Protein	X-ray	2003-05-06	
	B	330	77410.55	37914.60	196.11	Virb11 homolog	Hydrolase	Selenomethionine	MSE	Protein	X-ray	2003-05-06	
	B	330	77410.55	37914.60	96.06	Virb11 homolog	Hydrolase	Sulfate ion	SO4	Protein	X-ray	2003-05-06	
**1NLZ**	A	330	227487.61	37914.60	196.11	Virb11 homolog	Hydrolase	Selenomethionine	MSE	Protein	X-ray	2003-05-06	
	B	330	227487.61	37914.60	196.11	Virb11 homolog	Hydrolase	Selenomethionine	MSE	Protein	X-ray	2003-05-06	
	C	330	227487.61	37914.60	196.11	Virb11 homolog	Hydrolase	Selenomethionine	MSE	Protein	X-ray	2003-05-06	
	D	330	227487.61	37914.60	196.11	Virb11 homolog	Hydrolase	Selenomethionine	MSE	Protein	X-ray	2003-05-06	
	E	330	227487.61	37914.60	196.11	Virb11 homolog	Hydrolase	Selenomethionine	MSE	Protein	X-ray	2003-05-06	
	F	330	227487.61	37914.60	196.11	Virb11 homolog	Hydrolase	Selenomethionine	MSE	Protein	X-ray	2003-05-06	
**1OPX**	A	330	76435.81	37914.60	196.11	Virb11 homolog	Hydrolase	Selenomethionine	MSE	Protein	X-ray	2003-05-06	
**1OPX**	A	330	76435.81	37914.60	96.06	Virb11 homolog	Hydrolase	Sulfate ion	SO4	Protein	X-ray	2003-05-06	
	B	330	76435.81	37914.60	414.49	Virb11 homolog	Hydrolase	Nonaethylene glycol	2PE	Protein	X-ray	2003-05-06	
	B	330	76435.81	37914.60	196.11	Virb11 homolog	Hydrolase	Selenomethionine	MSE	Protein	X-ray	2003-05-06	
	B	330	76435.81	37914.60	96.06	Virb11 homolog	Hydrolase	Sulfate ion	SO4	Protein	X-ray	2003-05-06	
**1OT0**	A	20	2396.99	2396.99	16.02	50sprotein L1	Antibiotic	Amino group	NH2	Protein	NMR	2004-09-07	
**1OUV**	A	273	29972.10	29972.10		Conservedprotein	Hydrolase			Protein	X-ray	2004-03-30	

**1PU6**	A	218	50786.53	25218.30	78.13	3-methyladenine dna glycosylase	Hydrolase	Beta-mercaptoethanol	BME	Protein	X-ray	2003-10-07
	A	218	50786.53	25218.30	35.45	3-methyladenine dna glycosylase	Hydrolase	Chloride ion	CL	Protein	X-ray	2003-10-07
	A	218	50786.53	25218.30	190.20	3-methyladenine dna glycosylase	Hydrolase	Lysine nz-carboxylic acid	KCX	Protein	X-ray	2003-10-07
	B	218	50786.53	25218.30	190.20	3-methyladenine dna glycosylase	Hydrolase	Lysine nz-carboxylic acid	KCX	Protein	X-ray	2003-10-07
	B	218	50786.53	25218.30	118.18	3-methyladenine dna glycosylase	Hydrolase	(4s)-2-methyl-2,4-pentanediol	MPD	Protein	X-ray	2003-10-07
**1PU7**	A	218	50921.24	25218.30	164.19	3-methyladenine dna glycosylase	Hydrolase	6-amino-3,9-dimethyl-9h-purin-3-ium	39A	Protein	X-ray	2003-10-07
	A	218	50921.24	25218.30	78.13	3-methyladenine dna glycosylase	Hydrolase	Beta-mercaptoethanol	BME	Protein	X-ray	2003-10-07
	A	218	50921.24	25218.30	190.20	3-methyladenine dna glycosylase	Hydrolase	Lysine nz-carboxylic acid	KCX	Protein	X-ray	2003-10-07
	B	218	50921.24	25218.30	164.19	3-methyladenine dna glycosylase	Hydrolase	6-amino-3,9-dimethyl-9h-purin-3-ium	39A	Protein	X-ray	2003-10-07
	B	218	50921.24	25218.30	78.13	3-methyladenine dna glycosylase	Hydrolase	Beta-mercaptoethanol	BME	Protein	X-ray	2003-10-07
	B	218	50921.24	25218.30	190.20	3-methyladenine dna glycosylase	Hydrolase	Lysine nz-carboxylic acid	KCX	Protein	X-ray	2003-10-07
**1PU8**	A	218	50911.16	25218.30	78.13	3-methyladenine dna glycosylase	Hydrolase	Beta-mercaptoethanol	BME	Protein	X-ray	2003-10-07
	A	218	50911.16	25218.30	159.15	3-methyladenine dna glycosylase	Hydrolase	3h-imidazo[2,1-i]purine	EA1	Protein	X-ray	2003-10-07
	A	218	50911.16	25218.30	190.20	3-methyladenine dna glycosylase	Hydrolase	Lysine nz-carboxylic acid	KCX	Protein	X-ray	2003-10-07
	B	218	50911.16	25218.30	78.13	3-methyladenine dna glycosylase	Hydrolase	Beta-mercaptoethanol	BME	Protein	X-ray	2003-10-07
	B	218	50911.16	25218.30	159.15	3-methyladenine dna glycosylase	Hydrolase	3h-imidazo[2,1-i]purine	EA1	Protein	X-ray	2003-10-07
	B	218	50911.16	25218.30	190.20	3-methyladenine dna glycosylase	Hydrolase	Lysine nz-carboxylic acid	KCX	Protein	X-ray	2003-10-07
**1QWL**	A	505	118768.41	58730.70	616.50	Kata catalase	Oxidoreductase	Protoporphyrin ix containing fe	HEM	Protein	X-ray	2004-03-30
	A	505	118768.41	58730.70	32.00	Kata catalase	Oxidoreductase	Oxygen molecule	OXY	Protein	X-ray	2004-03-30
	B	505	118768.41	58730.70	42.02	Kata catalase	Oxidoreductase	Azide ion	AZI	Protein	X-ray	2004-03-30
	B	505	118768.41	58730.70	616.50	Kata catalase	Oxidoreductase	Protoporphyrin ix containing fe	HEM	Protein	X-ray	2004-03-30
**1QWM**	A	505	120393.35	58730.70	42.02	Kata catalase	Oxidoreductase	Azide ion	AZI	Protein	X-ray	2004-03-30
	A	505	120393.35	58730.70	46.03	Kata catalase	Oxidoreductase	Formic acid	FMT	Protein	X-ray	2004-03-30
	A	505	120393.35	58730.70	616.50	Kata catalase	Oxidoreductase	Protoporphyrin ix containing fe	HEM	Protein	X-ray	2004-03-30
	B	505	120393.35	58730.70	46.03	Kata catalase	Oxidoreductase	Formic acid	FMT	Protein	X-ray	2004-03-30
	B	505	120393.35	58730.70	616.50	Kata catalase	Oxidoreductase	Protoporphyrin ix containing fe	HEM	Protein	X-ray	2004-03-30
**1S2X**	A	206	23998.70	23938.60	60.10	Cag-Z	Unknown function	Isopropyl alcohol	IPA	Protein	X-ray	2004-07-27
	A	206	23998.70	23938.60	196.11	Cag-Z	Unknown function	Selenomethionine	MSE	Protein	X-ray	2004-07-27
**1UHD**	A	96	13339.46	10533.20	90.08	Aspartate 1-decarboxylase alpha chain	Lyase	Lactic acid	LAC	Protein	X-ray	2004-07-13
	B	24	13339.46	2806.26		Aspartate 1-decarboxylase beta chain	Lyase			Protein	X-ray	2004-07-13
**1UHE**	A	97	13744.94	10737.50	201.18	Aspartate 1-decarboxylase alpha chain	Lyase	N~2~-(2-amino-1-methyl-2-	NSN	Protein	X-ray	2004-07-13

								oxoethylidene)asparaginate					
	B	24	13744.94	2806.26		Aspartate 1-decarboxylase beta chain	Lyase			Protein	X-ray	2004-07-13	
**1UM0**	A	365	162446.59	40155.30	456.35	Chorismate synthase	Lyase	Flavin mononucleotide	FMN	Protein	X-ray	2004-06-01	
	B	365	162446.59	40155.30	456.35	Chorismate synthase	Lyase	Flavin mononucleotide	FMN	Protein	X-ray	2004-06-01	
	C	365	162446.59	40155.30	456.35	Chorismate synthase	Lyase	Flavin mononucleotide	FMN	Protein	X-ray	2004-06-01	
	D	365	162446.59	40155.30	456.35	Chorismate synthase	Lyase	Flavin mononucleotide	FMN	Protein	X-ray	2004-06-01	
**1UM8**	A	376	42519.20	42092.00	427.20	ATP-dependent Clp protease ATP-binding subunit clpx	Chaperone	Adenosine-5'-diphosphate	ADP	Protein	X-ray	2003-12-23	
**1UMF**	A	365	160621.20	40155.30		Chorismate synthase	Lyase			Protein	X-ray	2004-06-01	
	B	365	160621.20	40155.30		Chorismate synthase	Lyase			Protein	X-ray	2004-06-01	
	C	365	160621.20	40155.30		Chorismate synthase	Lyase			Protein	X-ray	2004-06-01	
	D	365	160621.20	40155.30		Chorismate synthase	Lyase			Protein	X-ray	2004-06-01	
**1X93**	A	55	13142.92	6571.46		Hypothetical protein HP0222	Transcription			Protein	NMR	2005-03-22	
	B	55	13142.92	6571.46		Hypothetical protein HP0222	Transcription			Protein	NMR	2005-03-22	
**1XNG**	A	268	63146.42	30376.30	507.18	NH(3)-dependent NAD(+) synthetase	Ligase	Adenosine-5'-triphosphate	ATP	Protein	X-ray	2005-04-05	
	A	268	63146.42	30376.30	665.42	NH(3)-dependent NAD(+) synthetase	Ligase	Nicotinic acid adenine dinucleotide	DND	Protein	X-ray	2005-04-05	
	A	268	63146.42	30376.30	24.31	NH(3)-dependent NAD(+) synthetase	Ligase	Magnesium ion	MG	Protein	X-ray	2005-04-05	
	B	268	63146.42	30376.30	507.18	NH(3)-dependent NAD(+) synthetase	Ligase	Adenosine-5'-triphosphate	ATP	Protein	X-ray	2005-04-05	
	B	268	63146.42	30376.30	665.42	NH(3)-dependent NAD(+) synthetase	Ligase	Nicotinic acid adenine dinucleotide	DND	Protein	X-ray	2005-04-05	
	B	268	63146.42	30376.30	24.31	NH(3)-dependent NAD(+) synthetase	Ligase	Magnesium ion	MG	Protein	X-ray	2005-04-05	
**1XNH**	A	268	30376.30	30376.30		NH(3)-dependent NAD(+) synthetase	Ligase			Protein	X-ray	2005-04-05	
**1YG0**	A	66	7203.31	7203.31		Copprotein	Metal transport			Protein	NMR	2006-01-24	
**1YGZ**	A	173	116914.20	19485.70	196.11	Inorganic pyrophosphatase	Hydrolase	Selenomethionine	MSE	Protein	X-ray	2005-11-01	
	B	173	116914.20	19485.70	196.11	Inorganic pyrophosphatase	Hydrolase	Selenomethionine	MSE	Protein	X-ray	2005-11-01	
	C	173	116914.20	19485.70	196.11	Inorganic pyrophosphatase	Hydrolase	Selenomethionine	MSE	Protein	X-ray	2005-11-01	
	D	173	116914.20	19485.70	196.11	Inorganic pyrophosphatase	Hydrolase	Selenomethionine	MSE	Protein	X-ray	2005-11-01	
	E	173	116914.20	19485.70	196.11	Inorganic pyrophosphatase	Hydrolase	Selenomethionine	MSE	Protein	X-ray	2005-11-01	
	F	173	116914.20	19485.70	196.11	Inorganic pyrophosphatase	Hydrolase	Selenomethionine	MSE	Protein	X-ray	2005-11-01	
**1Z8M**	A	88	10394.30	10394.30		Conservedprotein HP0894	SG, Unknown function			Protein	NMR	2005-11-01	
**1ZHC**	A	76	9130.38	9130.38		Hypothetical protein HP1242	Unknown function			Protein	NMR	2005-12-06	
**1ZKE**	A	83	56798.00	9450.13		Hypothetical protein HP1531	SG, unknown function			Protein	X-ray	2005-06-28	MCSG

	B	83	56798.00	9450.13	24.31	Hypothetical protein HP1531	SG, unknown function	Magnesium ion	MG	Protein	X-ray	2005-06-28	MCSG
	C	83	56798.00	9450.13	24.31	Hypothetical protein HP1531	SG, unknown function	Magnesium ion	MG	Protein	X-ray	2005-06-28	MCSG
	D	83	56798.00	9450.13	24.31	Hypothetical protein HP1531	SG, unknown function	Magnesium ion	MG	Protein	X-ray	2005-06-28	MCSG
	E	83	56798.00	9450.13	24.31	Hypothetical protein HP1531	SG, unknown function	Magnesium ion	MG	Protein	X-ray	2005-06-28	MCSG
	F	83	56798.00	9450.13		Hypothetical protein HP1531	SG, unknown function			Protein	X-ray	2005-06-28	MCSG
**1ZOF**	A	198	222768.00	22276.80		Alkyl hydroperoxide-reductase	Oxidoreductase			Protein	X-ray	2005-11-29	
	B	198	222768.00	22276.80		Alkyl hydroperoxide-reductase	Oxidoreductase			Protein	X-ray	2005-11-29	
	C	198	222768.00	22276.80		Alkyl hydroperoxide-reductase	Oxidoreductase			Protein	X-ray	2005-11-29	
	D	198	222768.00	22276.80		Alkyl hydroperoxide-reductase	Oxidoreductase			Protein	X-ray	2005-11-29	
	E	198	222768.00	22276.80		Alkyl hydroperoxide-reductase	Oxidoreductase			Protein	X-ray	2005-11-29	
	F	198	222768.00	22276.80		Alkyl hydroperoxide-reductase	Oxidoreductase			Protein	X-ray	2005-11-29	
	G	198	222768.00	22276.80		Alkyl hydroperoxide-reductase	Oxidoreductase			Protein	X-ray	2005-11-29	
	H	198	222768.00	22276.80		Alkyl hydroperoxide-reductase	Oxidoreductase			Protein	X-ray	2005-11-29	
	I	198	222768.00	22276.80		Alkyl hydroperoxide-reductase	Oxidoreductase			Protein	X-ray	2005-11-29	
	J	198	222768.00	22276.80		Alkyl hydroperoxide-reductase	Oxidoreductase			Protein	X-ray	2005-11-29	
**1ZUH**	A	168	19265.50	19265.50		Shikimate kinase	Transferase			Protein	X-ray	2006-05-31	
**1ZUI**	A	168	19534.62	19265.50	94.97	Shikimate kinase	Transferase	Phosphate ion	PO4	Protein	X-ray	2006-05-31	
	A	168	19534.62	19265.50	174.15	Shikimate kinase	Transferase	(3r,4s,5r)-3,4,5-trihydroxycyclohex-1-ene-1-carboxylic acid	SKM	Protein	X-ray	2006-05-31	
**2A6M**	A	155	36212.60	18106.30		Ishp608 transposase	Transcription/dna			Protein	X-ray	2005-10-25	
	B	155	36212.60	18106.30		Ishp608 transposase	Transcription/dna			Protein	X-ray	2005-10-25	
**2A6O**	A	155	49699.42	18106.30		Ishp608 Transposase	Transcription/dna			Protein DNA	X-ray	2005-10-25	
	B	155	49699.42	18106.30		Ishp608 Transposase	Transcription/dna			Protein DNA	X-ray	2005-10-25	
	C	22	49699.42	6743.41		5'-d(*cp*cp*cp*cp*tp*ap*gp*cp*tp*tp*tp*ap*gp*cp*tp*ap*tp*gp*gp*gp*gp*a)-3'	Transcription/dna			Protein DNA	X-ray	2005-10-25	
	D	22	49699.42	6743.41		5'-d(*cp*cp*cp*cp*tp*ap*gp*cp*tp*tp*t	Transcription/dna			Protein DNA	X-ray	2005-10-25	

						p*ap*gp*cp*tp*ap*tp*gp*gp*gp*gp*a)-3'							
**2A9E**	A	505	118913.44	58794.70	59.04	Kata Catalase	Oxidoreductase	Acetate ion	ACT	Protein	X-ray	2006-06-20	
	A	505	118913.44	58794.70	616.50	Kata Catalase	Oxidoreductase	Protoporphyrin ix containing fe	HEM	Protein	X-ray	2006-06-20	
	A	505	118913.44	58794.70	165.21	Kata Catalase	Oxidoreductase	S-oxymethionine	MHO	Protein	X-ray	2006-06-20	
	A	505	118913.44	58794.70	16.00	Kata Catalase	Oxidoreductase	Oxygen atom	O	Protein	X-ray	2006-06-20	
	B	505	118913.44	58794.70	616.50	Kata Catalase	Oxidoreductase	Protoporphyrin ix containing fe	HEM	Protein	X-ray	2006-06-20	
	B	505	118913.44	58794.70	165.21	Kata Catalase	Oxidoreductase	S-oxymethionine	MHO	Protein	X-ray	2006-06-20	
	B	505	118913.44	58794.70	16.00	Kata Catalase	Oxidoreductase	Oxygen atom	O	Protein	X-ray	2006-06-20	
**2ATZ**	A	180	22049.45	21480.20	507.18	H. Pylori predicted coding region hp0184	SG, unknown function	2'-deoxyguanosine-5'-triphosphate	DGT	Protein	X-ray	2005-10-11	MCSG
	A	180	22049.45	21480.20	62.07	H. Pylori predicted coding region hp0184	SG, unknown function	1,2-ethanediol	EDO	Protein	X-ray	2005-10-11	MCSG
	A	180	22049.45	21480.20	196.11	H. Pylori predicted coding region hp0184	SG, unknown function	Selenomethionine	MSE	Protein	X-ray	2005-10-11	MCSG
**2B7N**	A	273	93342.24	30850.90	167.12	Probable nicotinate-nucleotide pyrophosphorylase	Transferase	Quinolinic acid	NTM	Protein	X-ray	2006-02-14	
	A	273	93342.24	30850.90	96.06	Probable nicotinate-nucleotide pyrophosphorylase	Transferase	Sulfate ion	SO4	Protein	X-ray	2006-02-14	
	B	273	93342.24	30850.90	167.12	Probable nicotinate-nucleotide pyrophosphorylase	Transferase	Quinolinic acid	NTM	Protein	X-ray	2006-02-14	
	B	273	93342.24	30850.90	96.06	Probable nicotinate-nucleotide pyrophosphorylase	Transferase	Sulfate ion	SO4	Protein	X-ray	2006-02-14	
	C	273	93342.24	30850.90	167.12	Probable nicotinate-nucleotide pyrophosphorylase	Transferase	Quinolinic acid	NTM	Protein	X-ray	2006-02-14	
	C	273	93342.24	30850.90	96.06	Probable nicotinate-nucleotide pyrophosphorylase	Transferase	Sulfate ion	SO4	Protein	X-ray	2006-02-14	
**2B7P**	A	273	93339.28	30850.90	166.13	Probable nicotinate-nucleotide pyrophosphorylase	Transferase	Phthalic acid	PHT	Protein	X-ray	2006-02-14	
	A	273	93339.28	30850.90	96.06	Probable nicotinate-nucleotide pyrophosphorylase	Transferase	Sulfate ion	SO4	Protein	X-ray	2006-02-14	

	B	273	93339.28	30850.90	166.13	Probable nicotinate-nucleotide pyrophosphorylase	Transferase	Phthalic acid	PHT	Protein	X-ray	2006-02-14
	B	273	93339.28	30850.90	96.06	Probable nicotinate-nucleotide pyrophosphorylase	Transferase	Sulfate ion	SO4	Protein	X-ray	2006-02-14
	C	273	93339.28	30850.90	166.13	Probable nicotinate-nucleotide pyrophosphorylase	Transferase	Phthalic acid	PHT	Protein	X-ray	2006-02-14
	C	273	93339.28	30850.90	96.06	Probable nicotinate-nucleotide pyrophosphorylase	Transferase	Sulfate ion	SO4	Protein	X-ray	2006-02-14
**2B7Q**	A	273	93558.32	30850.90	335.21	Probable nicotinate-nucleotide pyrophosphorylase	Transferase	Nicotinate mononucleotide	NCN	Protein	X-ray	2006-02-21
	B	273	93558.32	30850.90	335.21	Probable nicotinate-nucleotide pyrophosphorylase	Transferase	Nicotinate mononucleotide	NCN	Protein	X-ray	2006-02-21
	C	273	93558.32	30850.90	335.21	Probable nicotinate-nucleotide pyrophosphorylase	Transferase	Nicotinate mononucleotide	NCN	Protein	X-ray	2006-02-21
**2BHV**	A	246	163102.81	27183.80		Comb10	Bacterial protein			Protein	X-ray	2005-03-16
	B	246	163102.81	27183.80		Comb10	Bacterial protein			Protein	X-ray	2005-03-16
	C	246	163102.81	27183.80		Comb10	Bacterial protein			Protein	X-ray	2005-03-16
	D	246	163102.81	27183.80		Comb10	Bacterial protein			Protein	X-ray	2005-03-16
	E	246	163102.81	27183.80		Comb10	Bacterial protein			Protein	X-ray	2005-03-16
	F	246	163102.81	27183.80		Comb10	Bacterial protein			Protein	X-ray	2005-03-16
**2BMV**	A	164	17668.11	17512.50	120.15	Flavodoxin	Electron transport	Benzamidine	BEN	Protein	X-ray	2006-06-22
	A	164	17668.11	17512.50	35.45	Flavodoxin	Electron transport	Chloride ion	CL	Protein	X-ray	2006-06-22
**2BO3**	A	94	11101.70	11101.70		Hypothetical protein hp0242	SG,unknown function			Protein	X-ray	2006-06-22
**2BQX**	A	173	19298.10	19298.10		Inorganic pyrophosphatase	Hydrolase			Protein	X-ray	2006-10-18
**2BQY**	A	173	19474.06	19298.10	175.96	Inorganic pyrophosphatase	Hydrolase	Pyrophosphate 2-	POP	Protein	X-ray	2006-10-18
**2C4V**	A	167	18691.53	18499.40	192.12	3-dehydroquinate dehydratase	Lyase	Citric acid	CIT	Protein	X-ray	2006-02-22
**2C4W**	A	176	20056.08	19390.40	343.32	3-dehydroquinate dehydratase	Lyase	N-tetrazol-5-yl 9-oxo-9h-xanthene-2 sulphonamide	GAJ	Protein	X-ray	2006-02-22
	A	176	20056.08	19390.40	92.09	3-dehydroquinate dehydratase	Lyase	Glycerol	GOL	Protein	X-ray	2006-02-22
	A	176	20056.08	19390.40	69.09	3-dehydroquinate dehydratase	Lyase	Imidazole	IMD	Protein	X-ray	2006-02-22
**2C57**	A	180	241596.66	19958.90	174.15	3-dehydroquinate dehydratase	Lyase	2,3 -anhydro-quinic acid	FA1	Protein	X-ray	2006-02-22

	B	180	241596.66	19958.90	174.15	3-dehydroquinate dehydratase	Lyase	2,3 -anhydro-quinic acid	FA1	Protein	X-ray	2006-02-22
	C	180	241596.66	19958.90	174.15	3-dehydroquinate dehydratase	Lyase	2,3 -anhydro-quinic acid	FA1	Protein	X-ray	2006-02-22
	D	180	241596.66	19958.90	174.15	3-dehydroquinate dehydratase	Lyase	2,3 -anhydro-quinic acid	FA1	Protein	X-ray	2006-02-22
	E	180	241596.66	19958.90	174.15	3-dehydroquinate dehydratase	Lyase	2,3 -anhydro-quinic acid	FA1	Protein	X-ray	2006-02-22
	F	180	241596.66	19958.90	174.15	3-dehydroquinate dehydratase	Lyase	2,3 -anhydro-quinic acid	FA1	Protein	X-ray	2006-02-22
	G	180	241596.66	19958.90	174.15	3-dehydroquinate dehydratase	Lyase	2,3 -anhydro-quinic acid	FA1	Protein	X-ray	2006-02-22
	H	180	241596.66	19958.90	174.15	3-dehydroquinate dehydratase	Lyase	2,3 -anhydro-quinic acid	FA1	Protein	X-ray	2006-02-22
	I	180	241596.66	19958.90	174.15	3-dehydroquinate dehydratase	Lyase	2,3 -anhydro-quinic acid	FA1	Protein	X-ray	2006-02-22
	J	180	241596.66	19958.90	174.15	3-dehydroquinate dehydratase	Lyase	2,3 -anhydro-quinic acid	FA1	Protein	X-ray	2006-02-22
	K	180	241596.66	19958.90	174.15	3-dehydroquinate dehydratase	Lyase	2,3 -anhydro-quinic acid	FA1	Protein	X-ray	2006-02-22
	L	180	241596.66	19958.90	174.15	3-dehydroquinate dehydratase	Lyase	2,3 -anhydro-quinic acid	FA1	Protein	X-ray	2006-02-22
**2CA9**	A	148	34939.26	17170.40	46.03	Putative nickel-responsive regulator	Transcriptional regulation	Formic acid	FMT	Protein	X-ray	2006-07-17
	A	148	34939.26	17170.40	92.09	Putative nickel-responsive regulator	Transcriptional regulation	Glycerol	GOL	Protein	X-ray	2006-07-17
	B	148	34939.26	17170.40	46.03	Putative nickel-responsive regulator	Transcriptional regulation	Formic acid	FMT	Protein	X-ray	2006-07-17
	B	148	34939.26	17170.40	92.09	Putative nickel-responsive regulator	Transcriptional regulation	Glycerol	GOL	Protein	X-ray	2006-07-17
**2CAD**	A	148	35169.41	17170.40	46.03	Putative nickel-responsive regulator	Transcriptional regulation	Formic acid	FMT	Protein	X-ray	2006-07-17
	A	148	35169.41	17170.40	92.09	Putative nickel-responsive regulator	Transcriptional regulation	Glycerol	GOL	Protein	X-ray	2006-07-17
	A	148	35169.41	17170.40	58.70	Putative nickel-responsive regulator	Transcriptional regulation	Nickel (ii) ion	NI	Protein	X-ray	2006-07-17
	B	148	35169.41	17170.40	192.12	Putative nickel-responsive regulator	Transcriptional regulation	Citric acid	CIT	Protein	X-ray	2006-07-17
	B	148	35169.41	17170.40	92.09	Putative nickel-responsive regulator	Transcriptional regulation	Glycerol	GOL	Protein	X-ray	2006-07-17
	B	148	35169.41	17170.40	58.70	Putative nickel-responsive regulator	Transcriptional regulation	Nickel (ii) ion	NI	Protein	X-ray	2006-07-17

**2CAJ**	A	148	35046.22	17170.40	92.09	Putative nickel-responsive regulator	Transcriptional regulation	Glycerol	GOL	Protein	X-ray	2006-07-17
	B	148	35046.22	17170.40	35.45	Putative nickel-responsive regulator	Transcriptional regulation	Chloride ion	CL	Protein	X-ray	2006-07-17
	B	148	35046.22	17170.40	92.09	Putative nickel-responsive regulator	Transcriptional regulation	Glycerol	GOL	Protein	X-ray	2006-07-17
	B	148	35046.22	17170.40	58.70	Putative nickel-responsive regulator	Transcriptional regulation	Nickel (ii) ion	NI	Protein	X-ray	2006-07-17
**2CMG**	A	262	61171.80	30585.90		Spermidine synthase	Transferase			Protein	X-ray	2007-05-08
	B	262	61171.80	30585.90		Spermidine synthase	Transferase			Protein	X-ray	2007-05-08
**2CMH**	A	262	91757.70	30585.90		Spermidine synthase	Transferase			Protein	X-ray	2007-05-08
	B	262	91757.70	30585.90		Spermidine synthase	Transferase			Protein	X-ray	2007-05-08
	C	262	91757.70	30585.90		Spermidine synthase	Transferase			Protein	X-ray	2007-05-08
**2CMU**	A	342	39349.10	39349.10	196.11	Putative peptidyl-arginine deiminase	Hydrolase	Selenomethionine	MSE	Protein	X-ray	2006-05-24
**2D2R**	A	245	57152.20	28576.10		Undecaprenyl pyrophosphate synthase	Transferase			Protein	X-ray	2006-09-26
	B	245	57152.20	28576.10		Undecaprenyl pyrophosphate synthase	Transferase			Protein	X-ray	2006-09-26
**2DTN**	A	245	57326.14	28576.10		Undecaprenyl pyrophosphate synthase	Transferase			Protein	X-ray	2007-06-26
	B	245	57326.14	28576.10	173.94	Undecaprenyl pyrophosphate synthase	Transferase	Diphosphate	DPO	Protein	X-ray	2007-06-26
**2DYU**	A	334	74661.40	37330.70		Formamidase	Hydrolase			Protein	X-ray	2007-02-13
	B	334	74661.40	37330.70		Formamidase	Hydrolase			Protein	X-ray	2007-02-13
**2DYV**	A	334	74661.40	37330.70		Formamidase	Hydrolase			Protein	X-ray	2007-02-13
	B	334	74661.40	37330.70		Formamidase	Hydrolase			Protein	X-ray	2007-02-13
**2E2K**	A	334	223888.19	37314.70		Formamidase	Hydrolase			Protein	X-ray	2007-02-13
	B	334	223888.19	37314.70		Formamidase	Hydrolase			Protein	X-ray	2007-02-13
	C	334	223888.19	37314.70		Formamidase	Hydrolase			Protein	X-ray	2007-02-13
	D	334	223888.19	37314.70		Formamidase	Hydrolase			Protein	X-ray	2007-02-13
	E	334	223888.19	37314.70		Formamidase	Hydrolase			Protein	X-ray	2007-02-13
	F	334	223888.19	37314.70		Formamidase	Hydrolase			Protein	X-ray	2007-02-13
**2E2L**	A	334	224023.31	37314.70	45.04	Formamidase	Hydrolase	Formamide	ARF	Protein	X-ray	2007-02-13
	B	334	224023.31	37314.70		Formamidase	Hydrolase			Protein	X-ray	2007-02-13
	C	334	224023.31	37314.70	45.04	Formamidase	Hydrolase	Formamide	ARF	Protein	X-ray	2007-02-13

	D	334	224023.31	37314.70		Formamidase	Hydrolase			Protein	X-ray	2007-02-13	
	E	334	224023.31	37314.70		Formamidase	Hydrolase			Protein	X-ray	2007-02-13	
	F	334	224023.31	37314.70	45.04	Formamidase	Hydrolase	Formamide	ARF	Protein	X-ray	2007-02-13	
**2EVV**	A	207	95692.83	23807.10	92.09	Hypothetical protein HP0218	SG, unknown function	Glycerol	GOL	Protein	X-ray	2005-12-13	MCSG
	A	207	95692.83	23807.10	196.11	Hypothetical protein HP0218	SG, unknown function	Selenomethionine	MSE	Protein	X-ray	2005-12-13	MCSG
	B	207	95692.83	23807.10	92.09	Hypothetical protein HP0218	SG, unknown function	Glycerol	GOL	Protein	X-ray	2005-12-13	MCSG
	B	207	95692.83	23807.10	196.11	Hypothetical protein HP0218	SG, unknown function	Selenomethionine	MSE	Protein	X-ray	2005-12-13	MCSG
	B	207	95692.83	23807.10	96.06	Hypothetical protein HP0218	SG, unknown function	Sulfate ion	SO4	Protein	X-ray	2005-12-13	MCSG
	C	207	95692.83	23807.10	92.09	Hypothetical protein HP0218	SG, unknown function	Glycerol	GOL	Protein	X-ray	2005-12-13	MCSG
	C	207	95692.83	23807.10	196.11	Hypothetical protein HP0218	SG, unknown function	Selenomethionine	MSE	Protein	X-ray	2005-12-13	MCSG
	D	207	95692.83	23807.10	92.09	Hypothetical protein HP0218	SG, unknown function	Glycerol	GOL	Protein	X-ray	2005-12-13	MCSG
	D	207	95692.83	23807.10	196.11	Hypothetical protein HP0218	SG, unknown function	Selenomethionine	MSE	Protein	X-ray	2005-12-13	MCSG
**2EW5**	A	181	21434.93	21008.60	58.93	Peptide deformylase	Hydrolase	Cobalt (ii) ion	CO	Protein	X-ray	2006-10-24	
	A	181	21434.93	21008.60	367.40	Peptide deformylase	Hydrolase	4-((1e)-3-oxo-3-[(2-phenylethyl)amino]prop-1-en-1-yl)-1,2-phenylene diacetate	Y12	Protein	X-ray	2006-10-24	
**2EW6**	A	181	21366.86	21008.60	58.93	Peptide deformylase	Hydrolase	Cobalt (ii) ion	CO	Protein	X-ray	2006-10-24	
	A	181	21366.86	21008.60	299.33	Peptide deformylase	Hydrolase	(2e)-3-(3,4-dihydroxyphenyl)-n-[2-(4-hydroxyphenyl)ethyl]acrylamide	Y13	Protein	X-ray	2006-10-24	
**2EW7**	A	181	21067.53	21008.60	58.93	Peptide deformylase	Hydrolase	Cobalt (ii) ion	CO	Protein	X-ray	2006-10-24	
**2F6S**	A	201	47249.90	23464.60	196.11	Cellprotein, putative	SG, unknown function	Selenomethionine	MSE	Protein	X-ray	2006-01-10	MCSG
	A	201	47249.90	23464.60	94.97	Cellprotein, putative	SG, unknown function	Phosphate ion	PO4	Protein	X-ray	2006-01-10	MCSG
	A	201	47249.90	23464.60	65.38	Cellprotein, putative	SG, unknown function	Zinc ion	ZN	Protein	X-ray	2006-01-10	MCSG
	B	201	47249.90	23464.60	196.11	Cellprotein, putative	SG, unknown function	Selenomethionine	MSE	Protein	X-ray	2006-01-10	MCSG
	B	201	47249.90	23464.60	94.97	Cellprotein, putative	SG, unknown function	Phosphate ion	PO4	Protein	X-ray	2006-01-10	MCSG
	B	201	47249.90	23464.60	65.38	Cellprotein, putative	SG, unknown function	Zinc ion	ZN	Protein	X-ray	2006-01-10	MCSG
**2FN6**	A	375	85108.34	42459.20	94.97	Aminotransferase	Transferase	Phosphate ion	PO4	Protein	X-ray	2006-01-24	MKBSGI[d]
	B	375	85108.34	42459.20	94.97	Aminotransferase	Transferase	Phosphate ion	PO4	Protein	X-ray	2006-01-24	MKBSGI
**2FNI**	A	375	85412.69	42459.20	247.14	Aminotransferase	Transferase	Pyridoxal-5'-phosphate	PLP	Protein	X-ray	2006-01-24	MKBSGI
	B	375	85412.69	42459.20	247.14	Aminotransferase	Transferase	Pyridoxal-5'-phosphate	PLP	Protein	X-ray	2006-01-24	MKBSGI
**2FNU**	A	375	86629.46	42459.20	248.18	Aminotransferase	Transferase	4'-deoxy-4'-aminopyridoxal-5'-	PMP	Protein	X-ray	2006-01-24	MKBSGI

								phosphate						
	A	375	86629.46	42459.20	607.36	Aminotransferase	Transferase	Uridine-diphosphate-n-acetylglucosamine	UD1	Protein	X-ray	2006-01-24	MKBSGI
	B	375	86629.46	42459.20	248.18	Aminotransferase	Transferase	4'-deoxy-4'-aminopyridoxal-5'-phosphate	PMP	Protein	X-ray	2006-01-24	MKBSGI
	B	375	86629.46	42459.20	607.36	Aminotransferase	Transferase	Uridine-diphosphate-n-acetylglucosamine	UD1	Protein	X-ray	2006-01-24	MKBSGI
**2G3V**	A	208	104975.36	25537.10	196.11	Cagprotein 13	Unknown function	Selenomethionine	MSE	Protein	X-ray	2007-03-06	
	B	208	104975.36	25537.10	196.11	Cagprotein 13	Unknown function	Selenomethionine	MSE	Protein	X-ray	2007-03-06	
	C	208	104975.36	25537.10	196.11	Cagprotein 13	Unknown function	Selenomethionine	MSE	Protein	X-ray	2007-03-06	
	D	208	104975.36	25537.10	196.11	Cagprotein 13	Unknown function	Selenomethionine	MSE	Protein	X-ray	2007-03-06	
	E	7	104975.36	706.74	196.11	(Unk)(unk)(unk)(unk)(unk)(mse)(unk)	Unknown function	Selenomethionine	MSE	Protein	X-ray	2007-03-06	
	E	7	104975.36	706.74	103.12	(Unk)(unk)(unk)(unk)(unk)(mse)(unk)	Unknown function	Unknown	UNK	Protein	X-ray	2007-03-06	
	F	7	104975.36	706.74	196.11	(Unk)(unk)(unk)(unk)(unk)(mse)(unk)	Unknown function	Selenomethionine	MSE	Protein	X-ray	2007-03-06	
	F	7	104975.36	706.74	103.12	(Unk)(unk)(unk)(unk)(unk)(mse)(unk)	Unknown function	Unknown	UNK	Protein	X-ray	2007-03-06	
	G	7	104975.36	706.74	196.11	(Unk)(unk)(unk)(unk)(unk)(mse)(unk)	Unknown function	Selenomethionine	MSE	Protein	X-ray	2007-03-06	
	G	7	104975.36	706.74	103.12	(Unk)(unk)(unk)(unk)(unk)(mse)(unk)	Unknown function	Unknown	UNK	Protein	X-ray	2007-03-06	
	H	7	104975.36	706.74	196.11	(Unk)(unk)(unk)(unk)(unk)(mse)(unk)	Unknown function	Selenomethionine	MSE	Protein	X-ray	2007-03-06	
	H	7	104975.36	706.74	103.12	(Unk)(unk)(unk)(unk)(unk)(mse)(unk)	Unknown function	Unknown	UNK	Protein	X-ray	2007-03-06	
**2GLL**	A	171	118844.75	19611.80	120.15	(3R)-hydroxymyristoyl-acylprotein dehydratase	Lyase	Benzamidine	BEN	Protein	X-ray	2007-03-13	
	A	171	118844.75	19611.80	35.45	(3R)-hydroxymyristoyl-acylprotein dehydratase	Lyase	Chloride ion	CL	Protein	X-ray	2007-03-13	
	B	171	118844.75	19611.80	120.15	(3R)-hydroxymyristoyl-acylprotein dehydratase	Lyase	Benzamidine	BEN	Protein	X-ray	2007-03-13	
	B	171	118844.75	19611.80	35.45	(3R)-hydroxymyristoyl-acylprotein dehydratase	Lyase	Chloride ion	CL	Protein	X-ray	2007-03-13	
	C	171	118844.75	19611.80	120.15	(3R)-hydroxymyristoyl-acylprotein dehydratase	Lyase	Benzamidine	BEN	Protein	X-ray	2007-03-13	
	C	171	118844.75	19611.80	35.45	(3R)-hydroxymyristoyl-acylprotein	Lyase	Chloride ion	CL	Protein	X-ray	2007-03-13	

						dehydratase						
	D	171	118844.75	19611.80	35.45	(3R)-hydroxymyristoyl-acylprotein dehydratase	Lyase	Chloride ion	CL	Protein	X-ray	2007-03-13
	E	171	118844.75	19611.80	120.15	(3R)-hydroxymyristoyl-acylprotein dehydratase	Lyase	Benzamidine	BEN	Protein	X-ray	2007-03-13
	E	171	118844.75	19611.80	35.45	(3R)-hydroxymyristoyl-acylprotein dehydratase	Lyase	Chloride ion	CL	Protein	X-ray	2007-03-13
	F	171	118844.75	19611.80	120.15	(3R)-hydroxymyristoyl-acylprotein dehydratase	Lyase	Benzamidine	BEN	Protein	X-ray	2007-03-13
	F	171	118844.75	19611.80	35.45	(3R)-hydroxymyristoyl-acylprotein dehydratase	Lyase	Chloride ion	CL	Protein	X-ray	2007-03-13
**2GLM**	A	171	119207.54	19611.80	120.15	(3R)-hydroxymyristoyl-acylprotein dehydratase	Lyase	Benzamidine	BEN	Protein	X-ray	2007-03-13
	A	171	119207.54	19611.80	35.45	(3R)-hydroxymyristoyl-acylprotein dehydratase	Lyase	Chloride ion	CL	Protein	X-ray	2007-03-13
	A	171	119207.54	19611.80	482.94	(3R)-hydroxymyristoyl-acylprotein dehydratase	Lyase	2-chloro-5-(5-((e)-[(2z)-3-(2-methoxyethyl)-4-oxo-2-(phenylimino)-1,3-thiazolidin-5-ylidene]methyl)-2-furyl)benzoic acid	SCB	Protein	X-ray	2007-03-13
	B	171	119207.54	19611.80	120.15	(3R)-hydroxymyristoyl-acylprotein dehydratase	Lyase	Benzamidine	BEN	Protein	X-ray	2007-03-13
	B	171	119207.54	19611.80	35.45	(3R)-hydroxymyristoyl-acylprotein dehydratase	Lyase	Chloride ion	CL	Protein	X-ray	2007-03-13
	C	171	119207.54	19611.80	120.15	(3R)-hydroxymyristoyl-acylprotein dehydratase	Lyase	Benzamidine	BEN	Protein	X-ray	2007-03-13
	C	171	119207.54	19611.80	35.45	(3R)-hydroxymyristoyl-acylprotein dehydratase	Lyase	Chloride ion	CL	Protein	X-ray	2007-03-13
	D	171	119207.54	19611.80	120.15	(3R)-hydroxymyristoyl-acylprotein dehydratase	Lyase	Benzamidine	BEN	Protein	X-ray	2007-03-13
	D	171	119207.54	19611.80	35.45	(3R)-hydroxymyristoyl-acylprotein dehydratase	Lyase	Chloride ion	CL	Protein	X-ray	2007-03-13

	E	171	119207.54	19611.80	120.15	(3R)-hydroxymyristoyl-acylprotein dehydratase	Lyase	Benzamidine	BEN	Protein	X-ray	2007-03-13
	F	171	119207.54	19611.80	35.45	(3R)-hydroxymyristoyl-acylprotein dehydratase	Lyase	Chloride ion	CL	Protein	X-ray	2007-03-13
**2GLP**	A	171	119915.14	19611.80	415.04	(3R)-hydroxymyristoyl-acylprotein dehydratase	Lyase	N'-[(1e)-(3,5-dibromo-2,4-dihydroxyphenyl)methylene]nicotinohydrazide	BDE	Protein	X-ray	2007-03-13
	A	171	119915.14	19611.80	120.15	(3R)-hydroxymyristoyl-acylprotein dehydratase	Lyase	Benzamidine	BEN	Protein	X-ray	2007-03-13
	A	171	119915.14	19611.80	35.45	(3R)-hydroxymyristoyl-acylprotein dehydratase	Lyase	Chloride ion	CL	Protein	X-ray	2007-03-13
	B	171	119915.14	19611.80	120.15	(3R)-hydroxymyristoyl-acylprotein dehydratase	Lyase	Benzamidine	BEN	Protein	X-ray	2007-03-13
	B	171	119915.14	19611.80	35.45	(3R)-hydroxymyristoyl-acylprotein dehydratase	Lyase	Chloride ion	CL	Protein	X-ray	2007-03-13
	C	171	119915.14	19611.80	415.04	(3R)-hydroxymyristoyl-acylprotein dehydratase	Lyase	N'-[(1e)-(3,5-dibromo-2,4-dihydroxyphenyl)methylene]nicotinohydrazide	BDE	Protein	X-ray	2007-03-13
	C	171	119915.14	19611.80	120.15	(3R)-hydroxymyristoyl-acylprotein dehydratase	Lyase	Benzamidine	BEN	Protein	X-ray	2007-03-13
	C	171	119915.14	19611.80	35.45	(3R)-hydroxymyristoyl-acylprotein dehydratase	Lyase	Chloride ion	CL	Protein	X-ray	2007-03-13
	D	171	119915.14	19611.80	35.45	(3R)-hydroxymyristoyl-acylprotein dehydratase	Lyase	Chloride ion	CL	Protein	X-ray	2007-03-13
	E	171	119915.14	19611.80	35.45	(3R)-hydroxymyristoyl-acylprotein dehydratase	Lyase	Chloride ion	CL	Protein	X-ray	2007-03-13
	F	171	119915.14	19611.80	120.15	(3R)-hydroxymyristoyl-acylprotein dehydratase	Lyase	Benzamidine	BEN	Protein	X-ray	2007-03-13
	F	171	119915.14	19611.80	35.45	(3R)-hydroxymyristoyl-acylprotein dehydratase	Lyase	Chloride ion	CL	Protein	X-ray	2007-03-13
**2GLV**	A	171	234661.83	19519.70	35.45	(3R)-hydroxymyristoyl-acylprotein	Lyase	Chloride ion	CL	Protein	X-ray	2007-03-13

						dehydratase						
	B	171	234661.83	19519.70	35.45	(3R)-hydroxymyristoyl-acylprotein dehydratase	Lyase	Chloride ion	CL	Protein	X-ray	2007-03-13
	C	171	234661.83	19519.70	35.45	(3R)-hydroxymyristoyl-acylprotein dehydratase	Lyase	Chloride ion	CL	Protein	X-ray	2007-03-13
	D	171	234661.83	19519.70	35.45	(3R)-hydroxymyristoyl-acylprotein dehydratase	Lyase	Chloride ion	CL	Protein	X-ray	2007-03-13
	E	171	234661.83	19519.70	35.45	(3R)-hydroxymyristoyl-acylprotein dehydratase	Lyase	Chloride ion	CL	Protein	X-ray	2007-03-13
	F	171	234661.83	19519.70	35.45	(3R)-hydroxymyristoyl-acylprotein dehydratase	Lyase	Chloride ion	CL	Protein	X-ray	2007-03-13
	G	171	234661.83	19519.70	35.45	(3R)-hydroxymyristoyl-acylprotein dehydratase	Lyase	Chloride ion	CL	Protein	X-ray	2007-03-13
	H	171	234661.83	19519.70		(3R)-hydroxymyristoyl-acylprotein dehydratase	Lyase			Protein	X-ray	2007-03-13
	I	171	234661.83	19519.70	35.45	(3R)-hydroxymyristoyl-acylprotein dehydratase	Lyase	Chloride ion	CL	Protein	X-ray	2007-03-13
	J	171	234661.83	19519.70		(3R)-hydroxymyristoyl-acylprotein dehydratase	Lyase			Protein	X-ray	2007-03-13
	K	171	234661.83	19519.70	35.45	(3R)-hydroxymyristoyl-acylprotein dehydratase	Lyase	Chloride ion	CL	Protein	X-ray	2007-03-13
	L	171	234661.83	19519.70		(3R)-hydroxymyristoyl-acylprotein dehydratase	Lyase			Protein	X-ray	2007-03-13
**2GN4**	A	344	80546.24	38725.10	745.43	UDP-glcnac C6 dehydratase	Lyase	Nadph dihydro-nicotinamide-adenine-dinucleotide phosphate	NDP	Protein	X-ray	2006-05-09
	A	344	80546.24	38725.10	607.36	UDP-glcnac C6 dehydratase	Lyase	Uridine-diphosphate-n-acetylglucosamine	UD1	Protein	X-ray	2006-05-09
	B	344	80546.24	38725.10	195.23	UDP-glcnac C6 dehydratase	Lyase	2-(n-morpholino)-ethanesulfonic acid	MES	Protein	X-ray	2006-05-09
	B	344	80546.24	38725.10	745.43	UDP-glcnac C6 dehydratase	Lyase	Nadph dihydro-nicotinamide-adenine-dinucleotide phosphate	NDP	Protein	X-ray	2006-05-09
	B	344	80546.24	38725.10	607.36	UDP-glcnac C6 dehydratase	Lyase	Uridine-diphosphate-n-	UD1	Protein	X-ray	2006-05-09

								acetylglucosamine				
**2GN6**	A	344	80346.97	38725.10	743.41	UDP-glcnac C6 dehydratase	Lyase	Nadp nicotinamide-adenine-dinucleotide phosphate	NAP	Protein	X-ray	2006-05-09
	A	344	80346.97	38725.10	607.36	UDP-glcnac C6 dehydratase	Lyase	Uridine-diphosphate-n-acetylglucosamine	UD1	Protein	X-ray	2006-05-09
	B	344	80346.97	38725.10	195.23	UDP-glcnac C6 dehydratase	Lyase	2-(n-morpholino)-ethanesulfonic acid	MES	Protein	X-ray	2006-05-09
	B	344	80346.97	38725.10	743.41	UDP-glcnac C6 dehydratase	Lyase	Nadp nicotinamide-adenine-dinucleotide phosphate	NAP	Protein	X-ray	2006-05-09
	B	344	80346.97	38725.10	607.36	UDP-glcnac C6 dehydratase	Lyase	Uridine-diphosphate-n-acetylglucosamine	UD1	Protein	X-ray	2006-05-09
**2GN8**	A	344	79940.58	38725.10	195.23	UDP-glcnac C6 dehydratase	Lyase	2-(n-morpholino)-ethanesulfonic acid	MES	Protein	X-ray	2006-05-09
	A	344	79940.58	38725.10	743.41	UDP-glcnac C6 dehydratase	Lyase	Nadp nicotinamide-adenine-dinucleotide phosphate	NAP	Protein	X-ray	2006-05-09
	A	344	79940.58	38725.10	404.16	UDP-glcnac C6 dehydratase	Lyase	Uridine-5'-diphosphate	UDP	Protein	X-ray	2006-05-09
	B	344	79940.58	38725.10	743.41	UDP-glcnac C6 dehydratase	Lyase	Nadp nicotinamide-adenine-dinucleotide phosphate	NAP	Protein	X-ray	2006-05-09
	B	344	79940.58	38725.10	404.16	UDP-glcnac C6 dehydratase	Lyase	Uridine-5'-diphosphate	UDP	Protein	X-ray	2006-05-09
**2GN9**	A	344	80264.87	38725.10	743.41	UDP-glcnac C6 dehydratase	Lyase	Nadp nicotinamide-adenine-dinucleotide phosphate	NAP	Protein	X-ray	2006-05-09
	A	344	80264.87	38725.10	566.30	UDP-glcnac C6 dehydratase	Lyase	Uridine-5'-diphosphate-glucose	UPG	Protein	X-ray	2006-05-09
	B	344	80264.87	38725.10	195.23	UDP-glcnac C6 dehydratase	Lyase	2-(n-morpholino)-ethanesulfonic acid	MES	Protein	X-ray	2006-05-09
	B	344	80264.87	38725.10	743.41	UDP-glcnac C6 dehydratase	Lyase	Nadp nicotinamide-adenine-dinucleotide phosphate	NAP	Protein	X-ray	2006-05-09
	B	344	80264.87	38725.10	566.30	UDP-glcnac C6 dehydratase	Lyase	Uridine-5'-diphosphate-glucose	UPG	Protein	X-ray	2006-05-09
**2GNA**	A	344	80264.87	38725.10	566.30	UDP-glcnac C6 dehydratase	Lyase	Galactose-uridine-5'-diphosphate	GDU	Protein	X-ray	2006-05-09
	A	344	80264.87	38725.10	743.41	UDP-glcnac C6 dehydratase	Lyase	Nadp nicotinamide-adenine-dinucleotide phosphate	NAP	Protein	X-ray	2006-05-09
	B	344	80264.87	38725.10	566.30	UDP-glcnac C6 dehydratase	Lyase	Galactose-uridine-5'-diphosphate	GDU	Protein	X-ray	2006-05-09
	B	344	80264.87	38725.10	195.23	UDP-glcnac C6 dehydratase	Lyase	2-(n-morpholino)-ethanesulfonic acid	MES	Protein	X-ray	2006-05-09
	B	344	80264.87	38725.10	743.41	UDP-glcnac C6 dehydratase	Lyase	Nadp nicotinamide-adenine-dinucleotide phosphate	NAP	Protein	X-ray	2006-05-09

**2GTS**	A	86	10626.50	10626.50	196.11	Hypothetical protein HP0062	SG, unknown function	Selenomethionine	MSE	Protein	X-ray	2006-05-30	MCSG
**2H0U**	A	217	26346.45	25890.10	456.35	NADPH-flavin oxidoreductase	Oxidoreductase	Flavin mononucleotide	FMN	Protein	X-ray	2006-06-13	MCSG
	A	217	26346.45	25890.10	196.11	NADPH-flavin oxidoreductase	Oxidoreductase	Selenomethionine	MSE	Protein	X-ray	2006-06-13	MCSG
**2H1Y**	A	321	71607.20	35803.60		Malonylprotein transacylase	Transferase			Protein	X-ray	2007-05-22	
	B	321	71607.20	35803.60		Malonylprotein transacylase	Transferase			Protein	X-ray	2007-05-22	
**2H9Z**	A	86	10205.80	10205.80		Hypothetical protein HP0495	SG, unknown function			Protein	NMR	2007-05-01	
**2HQN**	A	109	12481.40	12481.40		Putative transcriptional regulator	Signaling protein			Protein	NMR	2007-05-08	
**2HQO**	A	123	27757.80	13878.90		Putative transcriptional regulator	Signaling protein			Protein	NMR	2007-05-08	
	B	123	27757.80	13878.90		Putative transcriptional regulator	Signaling protein			Protein	NMR	2007-05-08	
**2HQR**	A	223	50946.60	25473.30		Putative transcriptional regulator	Signaling protein			Protein	NMR	2007-05-08	
	B	223	50946.60	25473.30		Putative transcriptional regulator	Signaling protein			Protein	NMR	2007-05-08	
**2I9I**	A	254	29526.70	29526.70		Hypothetical protein	SG, unknown function			Protein	X-ray	2006-09-26	NYSRCSG[e]
**2IQF**	A	505	118913.44	58794.70	59.04	Catalase	Oxidoreductase	Acetate ion	ACT	Protein	X-ray	2007-08-28	
	A	505	118913.44	58794.70	616.50	Catalase	Oxidoreductase	Protoporphyrin IX containing Fe	HEM	Protein	X-ray	2007-08-28	
	A	505	118913.44	58794.70	165.21	Catalase	Oxidoreductase	S-oxymethionine	MHO	Protein	X-ray	2007-08-28	
	A	505	118913.44	58794.70	16.00	Catalase	Oxidoreductase	Oxygen atom	O	Protein	X-ray	2007-08-28	
	B	505	118913.44	58794.70	616.50	Catalase	Oxidoreductase	Protoporphyrin IX containing Fe	HEM	Protein	X-ray	2007-08-28	
	B	505	118913.44	58794.70	165.21	Catalase	Oxidoreductase	S-oxymethionine	MHO	Protein	X-ray	2007-08-28	
	B	505	118913.44	58794.70	16.00	Catalase	Oxidoreductase	Oxygen atom	O	Protein	X-ray	2007-08-28	
**2JFX**	A	255	57359.06	28532.40	147.13	Glutamate racemase	Isomerase	D-glutamic acid	DGL	Protein	X-ray	2007-07-03	
	B	255	57359.06	28532.40	147.13	Glutamate racemase	Isomerase	D-glutamic acid	DGL	Protein	X-ray	2007-07-03	
**2JFY**	A	255	57359.06	28532.40	147.13	Glutamate racemase	Isomerase	D-glutamic acid	DGL	Protein	X-ray	2008-02-19	
	B	255	57359.06	28532.40	147.13	Glutamate racemase	Isomerase	D-glutamic acid	DGL	Protein	X-ray	2008-02-19	
**2JFZ**	A	255	58238.09	28532.40	439.52	Glutamate racemase	Isomerase	5-methyl-7-(2-methylpropyl)-2-(naphthalen-1-ylmethyl)-3-pyridin-4-yl-2h-pyrazolo[3,4-d]pyrimidine-4,6(5h,7h)-dione	003	Protein	X-ray	2007-07-03	
	A	255	58238.09	28532.40	147.13	Glutamate racemase	Isomerase	D-glutamic acid	DGL	Protein	X-ray	2007-07-03	
	B	255	58238.09	28532.40	439.52	Glutamate racemase	Isomerase	5-methyl-7-(2-methylpropyl)-2-(naphthalen-1-ylmethyl)-3-pyridin-4-yl-2h-pyrazolo[3,4-d]pyrimidine-	003	Protein	X-ray	2007-07-03	

								4,6(5h,7h)-dione					
	B	255	58238.09	28532.40	147.13	Glutamate racemase	Isomerase	D-glutamic acid	DGL	Protein	X-ray	2007-07-03	
**2JGQ**	A	233	53777.39	26612.00	94.97	Triosephosphate isomerase	Isomerase	Phosphate ion	PO4	Protein	X-ray	2008-02-26	
	A	233	53777.39	26612.00	363.45	Triosephosphate isomerase	Isomerase	1-[(3-cyclohexylpropanoyl)(2-hydroxyethyl)amino]-1-deoxy-d-allitol	QGA	Protein	X-ray	2008-02-26	
	B	233	53777.39	26612.00	94.97	Triosephosphate isomerase	Isomerase	Phosphate ion	PO4	Protein	X-ray	2008-02-26	
**2JOQ**	A	91	10673.20	10673.20		Hypothetical protein HP_0495	SG, unknown function			Protein	NMR	2007-04-03	OCSP[f]
	A	91	10673.20			Hypothetical protein HP_0495	SG, unknown function			Protein	NMR	2007-04-03	NSGC
**2K0Z**	A	110	12948.60	12948.60		Uncharacterized protein hp1203	SG, unknown function			Protein	NMR	2008-03-04	NSGC
**2K1O**	A	66	15603.66	7801.83		Putative	Gene regulation			Protein	NMR	2008-10-07	
	B	66	15603.66	7801.83		Putative	Gene regulation			Protein	NMR	2008-10-07	
**2K4J**	A	115	13303.10	13303.10		Putative TRANSCRIPTIONAL REGULATOR	Transcription			Protein	NMR	2008-12-30	
**2K6P**	A	92	10472.30	10472.30		Uncharacterized protein HP_1423	Unknown function			Protein	NMR	2009-06-16	
**2KDX**	A	119	13430.88	13365.50	65.38	Hydrogenase/ureaseprotein hypa	Metal-binding protein	Zinc ion	ZN	Protein	NMR	2009-10-20	
**2KI2**	A	90	10470.00	10470.00		Ss-dnaprotein 12RNP2	Rnaprotein/ss-dnaprotein			Protein	NMR	2009-10-20	
**2KR7**	A	151	17039.20	17039.20		FKBP-type peptidyl-prolyl cis-trans isomerase slyd	Isomerase			Protein	NMR	2010-12-15	
**2NQO**	A	376	121834.00	40506.70		Gamma-glutamyltranspeptidase	Transferase			Protein	X-ray	2006-11-21	
	B	188	121834.00	20410.30		Gamma-glutamyltranspeptidase	Transferase			Protein	X-ray	2006-11-21	
	C	376	121834.00	40506.70		Gamma-glutamyltranspeptidase	Transferase			Protein	X-ray	2006-11-21	
	D	188	121834.00	20410.30		Gamma-glutamyltranspeptidase	Transferase			Protein	X-ray	2006-11-21	
**2NZW**	A	371	129659.87	43123.90		Alpha1,3-fucosyltransferase	Transferase			Protein	X-ray	2007-01-23	
	B	371	129659.87	43123.90	96.06	Alpha1,3-fucosyltransferase	Transferase	Sulfate ion	SO4	Protein	X-ray	2007-01-23	
	C	371	129659.87	43123.90	96.06	Alpha1,3-fucosyltransferase	Transferase	Sulfate ion	SO4	Protein	X-ray	2007-01-23	
**2NZX**	A	371	130797.36	43123.90	443.20	Alpha1,3-fucosyltransferase	Transferase	Guanosine-5'-diphosphate	GDP	Protein	X-ray	2007-01-23	
	B	371	130797.36	43123.90	443.20	Alpha1,3-fucosyltransferase	Transferase	Guanosine-5'-diphosphate	GDP	Protein	X-ray	2007-01-23	
	C	371	130797.36	43123.90	443.20	Alpha1,3-fucosyltransferase	Transferase	Guanosine-5'-diphosphate	GDP	Protein	X-ray	2007-01-23	
	C	371	130797.36	43123.90	96.06	Alpha1,3-fucosyltransferase	Transferase	Sulfate ion	SO4	Protein	X-ray	2007-01-23	
**2NZY**	A	371	131385.89	43123.90	164.16	Alpha1,3-fucosyltransferase	Transferase	Alpha-l-fucose	FUC	Protein	X-ray	2007-01-23	

	A	371	131385.89	43123.90	443.20	Alpha1,3-fucosyltransferase	Transferase	Guanosine-5'-diphosphate	GDP	Protein	X-ray	2007-01-23	
	B	371	131385.89	43123.90	164.16	Alpha1,3-fucosyltransferase	Transferase	Alpha-l-fucose	FUC	Protein	X-ray	2007-01-23	
	B	371	131385.89	43123.90	443.20	Alpha1,3-fucosyltransferase	Transferase	Guanosine-5'-diphosphate	GDP	Protein	X-ray	2007-01-23	
	B	371	131385.89	43123.90	96.06	Alpha1,3-fucosyltransferase	Transferase	Sulfate ion	SO4	Protein	X-ray	2007-01-23	
	C	371	131385.89	43123.90	164.16	Alpha1,3-fucosyltransferase	Transferase	Alpha-l-fucose	FUC	Protein	X-ray	2007-01-23	
	C	371	131385.89	43123.90	443.20	Alpha1,3-fucosyltransferase	Transferase	Guanosine-5'-diphosphate	GDP	Protein	X-ray	2007-01-23	
	C	371	131385.89	43123.90	96.06	Alpha1,3-fucosyltransferase	Transferase	Sulfate ion	SO4	Protein	X-ray	2007-01-23	
**2ORM**	A	67	44336.70	7389.45		Probable tautomerase HP0924	Isomerase			Protein	X-ray	2008-02-12	
	B	67	44336.70	7389.45		Probable tautomerase HP0924	Isomerase			Protein	X-ray	2008-02-12	
	C	67	44336.70	7389.45		Probable tautomerase HP0924	Isomerase			Protein	X-ray	2008-02-12	
	D	67	44336.70	7389.45		Probable tautomerase HP0924	Isomerase			Protein	X-ray	2008-02-12	
	E	67	44336.70	7389.45		Probable tautomerase HP0924	Isomerase			Protein	X-ray	2008-02-12	
	F	67	44336.70	7389.45		Probable tautomerase HP0924	Isomerase			Protein	X-ray	2008-02-12	
**2OTR**	A	98	11502.60	11502.60		Hypothetical protein HP0892	SG, unknown function			Protein	NMR	2007-12-18	
**2OUF**	A	94	11148.60	11148.60	196.11	Hypothetical protein	SG, unknown function	Selenomethionine	MSE	Protein	X-ray	2007-02-27	MCSG
**2PD3**	A	275	123878.30	30016.60	663.43	Enoyl-[acyl-carrier-protein] reductase [NADH]	Oxidoreductase	Nicotinamide-adenine-dinucleotide	NAD	Protein	X-ray	2007-04-17	
	A	275	123878.30	30016.60	289.55	Enoyl-[acyl-carrier-protein] reductase [NADH]	Oxidoreductase	Triclosan	TCL	Protein	X-ray	2007-04-17	
	B	275	123878.30	30016.60	663.43	Enoyl-[acyl-carrier-protein] reductase [NADH]	Oxidoreductase	Nicotinamide-adenine-dinucleotide	NAD	Protein	X-ray	2007-04-17	
	B	275	123878.30	30016.60	289.55	Enoyl-[acyl-carrier-protein] reductase [NADH]	Oxidoreductase	Triclosan	TCL	Protein	X-ray	2007-04-17	
	C	275	123878.30	30016.60	663.43	Enoyl-[acyl-carrier-protein] reductase [NADH]	Oxidoreductase	Nicotinamide-adenine-dinucleotide	NAD	Protein	X-ray	2007-04-17	
	C	275	123878.30	30016.60	289.55	Enoyl-[acyl-carrier-protein] reductase [NADH]	Oxidoreductase	Triclosan	TCL	Protein	X-ray	2007-04-17	
	D	275	123878.30	30016.60	663.43	Enoyl-[acyl-carrier-protein] reductase [NADH]	Oxidoreductase	Nicotinamide-adenine-dinucleotide	NAD	Protein	X-ray	2007-04-17	
	D	275	123878.30	30016.60	289.55	Enoyl-[acyl-carrier-protein] reductase [NADH]	Oxidoreductase	Triclosan	TCL	Protein	X-ray	2007-04-17	

**2PD4**	A	275	123740.52	30016.60	255.10	Enoyl-[acyl-carrier-protein] reductase [NADH]	Oxidoreductase	Diclosan	DCN	Protein	X-ray	2007-04-17
	A	275	123740.52	30016.60	663.43	Enoyl-[acyl-carrier-protein] reductase [NADH]	Oxidoreductase	Nicotinamide-adenine-dinucleotide	NAD	Protein	X-ray	2007-04-17
	B	275	123740.52	30016.60	255.10	Enoyl-[acyl-carrier-protein] reductase [NADH]	Oxidoreductase	Diclosan	DCN	Protein	X-ray	2007-04-17
	B	275	123740.52	30016.60	663.43	Enoyl-[acyl-carrier-protein] reductase [NADH]	Oxidoreductase	Nicotinamide-adenine-dinucleotide	NAD	Protein	X-ray	2007-04-17
	C	275	123740.52	30016.60	255.10	Enoyl-[acyl-carrier-protein] reductase [NADH]	Oxidoreductase	Diclosan	DCN	Protein	X-ray	2007-04-17
	C	275	123740.52	30016.60	663.43	Enoyl-[acyl-carrier-protein] reductase [NADH]	Oxidoreductase	Nicotinamide-adenine-dinucleotide	NAD	Protein	X-ray	2007-04-17
	D	275	123740.52	30016.60	255.10	Enoyl-[acyl-carrier-protein] reductase [NADH]	Oxidoreductase	Diclosan	DCN	Protein	X-ray	2007-04-17
	D	275	123740.52	30016.60	663.43	Enoyl-[acyl-carrier-protein] reductase [NADH]	Oxidoreductase	Nicotinamide-adenine-dinucleotide	NAD	Protein	X-ray	2007-04-17
**2PLN**	A	137	15663.10	15663.10	196.11	Response regulator	Signaling protein	Selenomethionine	MSE	Protein	X-ray	2007-05-01
**2PT7**	A	330	260955.98	37633.20		Cag-alfa	Hydrolase/protein binding			Protein	X-ray	2007-11-13
	B	330	260955.98	37633.20		Cag-alfa	Hydrolase/protein binding			Protein	X-ray	2007-11-13
	C	330	260955.98	37633.20		Cag-alfa	Hydrolase/protein binding			Protein	X-ray	2007-11-13
	D	330	260955.98	37633.20		Cag-alfa	Hydrolase/protein binding			Protein	X-ray	2007-11-13
	E	330	260955.98	37633.20		Cag-alfa	Hydrolase/protein binding			Protein	X-ray	2007-11-13
	F	330	260955.98	37633.20		Cag-alfa	Hydrolase/protein binding			Protein	X-ray	2007-11-13
	G	152	260955.98	17578.40		Hypothetical protein	Hydrolase/protein binding			Protein	X-ray	2007-11-13

	H	152	260955.98	17578.40		Hypothetical protein	Hydrolase/protein binding			Protein	X-ray	2007-11-13	
**2PVP**	A	367	83921.40	41960.70		D-alanine-D-alanine ligase	Ligase			Protein	X-ray	2008-04-01	
	B	367	83921.40	41960.70		D-alanine-D-alanine ligase	Ligase			Protein	X-ray	2008-04-01	
**2PZH**	A	135	63418.00	15854.50		Hypothetical protein HP_0496	Hydrolase			Protein	X-ray	2008-04-08	
	B	135	63418.00	15854.50		Hypothetical protein HP_0496	Hydrolase			Protein	X-ray	2008-04-08	
	C	135	63418.00	15854.50		Hypothetical protein HP_0496	Hydrolase			Protein	X-ray	2008-04-08	
	D	135	63418.00	15854.50		Hypothetical protein HP_0496	Hydrolase			Protein	X-ray	2008-04-08	
**2Q0K**	A	311	70212.93	33577.50	785.56	Thioredoxin reductase	Oxidoreductase	Flavin-adenine dinucleotide	FAD	Protein	X-ray	2007-07-03	
	A	311	70212.93	33577.50	743.41	Thioredoxin reductase	Oxidoreductase	Nadp nicotinamide-adenine-dinucleotide phosphate	NAP	Protein	X-ray	2007-07-03	
	B	311	70212.93	33577.50	785.56	Thioredoxin reductase	Oxidoreductase	Flavin-adenine dinucleotide	FAD	Protein	X-ray	2007-07-03	
	B	311	70212.93	33577.50	743.41	Thioredoxin reductase	Oxidoreductase	Nadp nicotinamide-adenine-dinucleotide phosphate	NAP	Protein	X-ray	2007-07-03	
**2Q0L**	A	311	70212.93	33577.50	785.56	Thioredoxin reductase	Oxidoreductase	Flavin-adenine dinucleotide	FAD	Protein	X-ray	2007-07-03	
	A	311	70212.93	33577.50	743.41	Thioredoxin reductase	Oxidoreductase	Nadp nicotinamide-adenine-dinucleotide phosphate	NAP	Protein	X-ray	2007-07-03	
	B	311	70212.93	33577.50	785.56	Thioredoxin reductase	Oxidoreductase	Flavin-adenine dinucleotide	FAD	Protein	X-ray	2007-07-03	
	B	311	70212.93	33577.50	743.41	Thioredoxin reductase	Oxidoreductase	Nadp nicotinamide-adenine-dinucleotide phosphate	NAP	Protein	X-ray	2007-07-03	
**2QGH**	A	425	47813.44	47474.20	92.09	Diaminopimelate decarboxylase	Lyase	Glycerol	GOL	Protein	X-ray	2008-05-27	
	A	425	47813.44	47474.20	247.14	Diaminopimelate decarboxylase	Lyase	Pyridoxal-5'-phosphate	PLP	Protein	X-ray	2008-05-27	
**2QH5**	A	308	69996.80	34998.40		Mannose-6-phosphate isomerase	Isomerase			Protein	X-ray	2007-07-17	NYSRCSG
	B	308	69996.80	34998.40		Mannose-6-phosphate isomerase	Isomerase			Protein	X-ray	2007-07-17	NYSRCSG
**2QM6**	A	377	122270.46	40577.80		Gamma-glutamyltranspeptidase	Transferase			Protein	X-ray	2008-02-12	
	B	188	122270.46	20410.30		Gamma-glutamyltranspeptidase	Transferase			Protein	X-ray	2008-02-12	
	C	377	122270.46	40577.80		Gamma-glutamyltranspeptidase	Transferase			Protein	X-ray	2008-02-12	
	D	188	122270.46	20410.30		Gamma-glutamyltranspeptidase	Transferase			Protein	X-ray	2008-02-12	
**2QMC**	A	377	122801.09	40577.80		Gamma-glutamyltranspeptidase	Transferase			Protein	X-ray	2008-02-12	
	B	188	122801.09	20380.30	442.44	Gamma-glutamyltranspeptidase	Transferase	S-(p-nitrobenzyl)glutathione	GTB	Protein	X-ray	2008-02-12	

	C	377	122801.09	40577.80		Gamma-glutamyltranspeptidase	Transferase			Protein	X-ray	2008-02-12	
	D	188	122801.09	20380.30	442.44	Gamma-glutamyltranspeptidase	Transferase	S-(p-nitrobenzyl)glutathione	GTB	Protein	X-ray	2008-02-12	
**2QMO**	A	220	24888.11	24817.20	35.45	Dethiobiotin synthetase	Ligase	Chloride ion	CL	Protein	X-ray	2007-07-31	MCSG
	A	220	24888.11	24817.20	196.11	Dethiobiotin synthetase	Ligase	Selenomethionine	MSE	Protein	X-ray	2007-07-31	MCSG
**2QV3**	A	457	49391.60	49391.60		Vacuolating cytotoxin	Toxin			Protein	X-ray	2007-10-23	
**2R62**	A	268	58219.20	29109.60		Cell division protease ftsh homolog	Hydrolase			Protein	X-ray	2008-09-09	
	B	268	58219.20	29109.60		Cell division protease ftsh homolog	Hydrolase			Protein	X-ray	2008-09-09	
**2R65**	A	268	147684.02	29109.60	427.20	Cell division protease ftsh homolog	Hydrolase	Adenosine-5'-diphosphate	ADP	Protein	X-ray	2008-09-09	
	B	268	147684.02	29109.60	427.20	Cell division protease ftsh homolog	Hydrolase	Adenosine-5'-diphosphate	ADP	Protein	X-ray	2008-09-09	
	C	268	147684.02	29109.60	427.20	Cell division protease ftsh homolog	Hydrolase	Adenosine-5'-diphosphate	ADP	Protein	X-ray	2008-09-09	
	D	268	147684.02	29109.60	427.20	Cell division protease ftsh homolog	Hydrolase	Adenosine-5'-diphosphate	ADP	Protein	X-ray	2008-09-09	
	E	268	147684.02	29109.60	427.20	Cell division protease ftsh homolog	Hydrolase	Adenosine-5'-diphosphate	ADP	Protein	X-ray	2008-09-09	
**2RD3**	A	223	51330.60	25665.30		Transcriptional regulator	Hydrolase			Protein	X-ray	2008-09-30	
	D	223	51330.60	25665.30		Transcriptional regulator	Hydrolase			Protein	X-ray	2008-09-30	
**2UVP**	A	186	87079.82	21640.10	59.04	Hoba	Unknown function	Acetate ion	ACT	Protein	X-ray	2007-08-21	
	B	186	87079.82	21626.00	40.08	Hoba	Unknown function	Calcium ion	CA	Protein	X-ray	2007-08-21	
	B	186	87079.82	21626.00	92.09	Hoba	Unknown function	Glycerol	GOL	Protein	X-ray	2007-08-21	
	C	186	87079.82	21626.00	59.04	Hoba	Unknown function	Acetate ion	ACT	Protein	X-ray	2007-08-21	
	C	186	87079.82	21626.00	40.08	Hoba	Unknown function	Calcium ion	CA	Protein	X-ray	2007-08-21	
	D	186	87079.82	21626.00	92.09	Hoba	Unknown function	Glycerol	GOL	Protein	X-ray	2007-08-21	
**2VHG**	A	159	55926.58	18392.60	54.94	Transposase orfa	Dna-binding protein	Manganese (ii) ion	MN	Protein DNA	X-ray	2008-02-19	
	B	159	55926.58	18392.60		Transposase orfa	Dna-binding protein			Protein DNA	X-ray	2008-02-19	
	C	31	55926.58	9543.22		Right end 31-mer	Dna-binding protein			Protein DNA	X-ray	2008-02-19	
	D	31	55926.58	9543.22		Right end 31-mer	Dna-binding protein			Protein DNA	X-ray	2008-02-19	
**2VIC**	A	159	52901.54	18392.60	54.94	Transposase orfa	Dna-binding protein	Manganese (ii) ion	MN	Protein DNA	X-ray	2008-02-19	

	B	159	52901.54	18392.60		Transposase orfa	Dna-binding protein			Protein DNA	X-ray	2008-02-19
	C	26	52901.54	8003.23		5'-d(*ap*ap*ap*gp*cp*cp*cp*cp*tp*ap*gp*cp*tp*tp*tp*tp*ap*gp*cp*tp*ap*tp*gp*gp*gp*g)-3'	Dna-binding protein			Protein DNA	X-ray	2008-02-19
	D	26	52901.54	8003.23	54.94	5'-d(*ap*ap*ap*gp*cp*cp*cp*cp*tp*ap*gp*cp*tp*tp*tp*tp*ap*gp*cp*tp*ap*tp*gp*gp*gp*g)-3'	Dna-binding protein	Manganese (ii) ion	MN	Protein DNA	X-ray	2008-02-19
**2VIH**	A	159	52791.66	18392.60		Transposase orfa	Dna-binding protein			Protein DNA	X-ray	2008-02-19
	B	159	52791.66	18392.60		Transposase orfa	Dna-binding protein			Protein DNA	X-ray	2008-02-19
	C	26	52791.66	8003.23		5'-d(*ap*ap*ap*gp*cp*cp*cp*cp*tp*ap*gp*cp*tp*tp*tp*tp*ap*gp*cp*tp*ap*tp*gp*gp*gp*g)-3'	Dna-binding protein			Protein DNA	X-ray	2008-02-19
	D	26	52791.66	8003.23		5'-d(*ap*ap*ap*gp*cp*cp*cp*cp*tp*ap*gp*cp*tp*tp*tp*tp*ap*gp*cp*tp*ap*tp*gp*gp*gp*g)-3'	Dna-binding protein			Protein DNA	X-ray	2008-02-19
**2VJU**	A	159	58556.01	18392.60	54.94	Transposase orfa	Dna-binding protein	Manganese (ii) ion	MN	Protein DNA	X-ray	2008-02-19
	B	159	58556.01	18392.60		Transposase orfa	Dna-binding protein			Protein DNA	X-ray	2008-02-19
	C	35	58556.01	10803.00	54.94	Right end 35-mer	Dna-binding protein	Manganese (ii) ion	MN	Protein DNA	X-ray	2008-02-19
	D	35	58556.01	10803.00	54.94	Right end 35-mer	Dna-binding protein	Manganese (ii) ion	MN	Protein DNA	X-ray	2008-02-19
**2VJV**	A	159	56431.03	18392.60		Transposase orfa	Dna-binding protein			Protein	X-ray	2008-02-19

										DNA		
	B	159	56431.03	18392.60		Transposase orfa	Dna-binding protein			Protein DNA	X-ray	2008-02-19
	C	26	56431.03	8003.23	24.31	5'-d(*da*da*da*dg*dc*dc*dc*dc*dt*da*dg*dc*dtp*dt *dt*dt*da*dg*dc*dt*da*dt*dg*dg*dg*dgp)-3'	Dna-binding protein	Magnesium ion	MG	Protein DNA	X-ray	2008-02-19
	D	26	56431.03	8003.23	24.31	5'-d(*da*da*da*dg*dc*dc*dc*dc*dt*da*dg*dc*dtp*dt *dt*dt*da*dg*dc*dt*da*dt*dg*dg*dg*dgp)-3'	Dna-binding protein	Magnesium ion	MG	Protein DNA	X-ray	2008-02-19
	E	6	56431.03	1783.23	24.31	5'-d(*dt*da*dt*dt*da*dcp)-3'	Dna-binding protein	Magnesium ion	MG	Protein DNA	X-ray	2008-02-19
	F	6	56431.03	1783.23		5'-d(*dt*da*dt*dt*da*dcp)-3'	Dna-binding protein			Protein DNA	X-ray	2008-02-19
**2VW9**	A	134	40539.90	14969.00		SINGLE-strandedprotein	Dna-binding protein			Protein DNA	X-ray	2009-06-02
	B	134	40539.90	14969.00		SINGLE-strandedprotein	Dna-binding protein			Protein DNA	X-ray	2009-06-02
	C	35	40539.90	10601.90		Poly-dt	Dna-binding protein			Protein DNA	X-ray	2009-06-02
**2W4I**	A	255	115465.07	28532.40	147.13	Glutamate racemase	Isomerase	D-glutamic acid	DGL	Protein	X-ray	2008-12-09
	B	255	115465.07	28532.40	147.13	Glutamate racemase	Isomerase	D-glutamic acid	DGL	Protein	X-ray	2008-12-09
	B	255	115465.07	28532.40	373.47	Glutamate racemase	Isomerase	1-[(3s)-5-phenyl-3-thiophen-2-yl-3h-1,4-benzodiazepin-2-yl]azetidin-3-ol	VGA	Protein	X-ray	2008-12-09
	E	255	115465.07	28532.40	147.13	Glutamate racemase	Isomerase	D-glutamic acid	DGL	Protein	X-ray	2008-12-09
	E	255	115465.07	28532.40	373.47	Glutamate racemase	Isomerase	1-[(3s)-5-phenyl-3-thiophen-2-yl-3h-1,4-benzodiazepin-2-yl]azetidin-3-ol	VGA	Protein	X-ray	2008-12-09
	F	255	115465.07	28532.40	147.13	Glutamate racemase	Isomerase	D-glutamic acid	DGL	Protein	X-ray	2008-12-09

**2W5U**	A	164	36291.90	17540.50	456.35	Flavodoxin	Electron transport	Flavin mononucleotide	FMN	Protein	X-ray	2009-12-22
	A	164	36291.90	17540.50	298.20	Flavodoxin	Electron transport	[2-(5-amino-4-cyano-1h-pyrazol-1-yl)-5-(trifluoromethyl)phenyl](hydroxy)oxoammonium	IC3	Protein	X-ray	2009-12-22
	B	164	36291.90	17540.50	456.35	Flavodoxin	Electron transport	Flavin mononucleotide	FMN	Protein	X-ray	2009-12-22
**2WCQ**	A	159	36846.45	18231.10	192.12	TNF-alphaprotein	Immune system	Citric acid	CIT	Protein	X-ray	2009-05-12
	B	159	36846.45	18231.10	192.12	TNF-alphaprotein	Immune system	Citric acid	CIT	Protein	X-ray	2009-05-12
**2WCR**	A	159	36589.99	18231.10	69.09	TNF-alphaprotein	Immune system	Imidazole	IMD	Protein	X-ray	2009-05-12
	A	159	36589.99	18231.10	58.70	TNF-alphaprotein	Immune system	Nickel (ii) ion	NI	Protein	X-ray	2009-05-12
	B	159	36589.99	18231.10		TNF-alphaprotein	Immune system			Protein	X-ray	2009-05-12
**2WGL**	A	254	87363.00	29121.00	196.11	Ureaseprotein UREF	Metalprotein	Selenomethionine	MSE	Protein	X-ray	2009-05-26
	B	254	87363.00	29121.00	196.11	Ureaseprotein UREF	Metalprotein	Selenomethionine	MSE	Protein	X-ray	2009-05-26
	C	254	87363.00	29121.00	196.11	Ureaseprotein UREF	Metalprotein	Selenomethionine	MSE	Protein	X-ray	2009-05-26
**2WKS**	A	167	113098.72	18499.40	350.39	3-dehydroquinate dehydratase	Lyase	(1r,4s,5r)-1,4,5-trihydroxy-3-[(5-methyl-1-benzothiophen-2-yl)methoxy]cyclohex-2-ene-1-carboxylic acid	CB6	Protein	X-ray	2009-11-24
	B	167	113098.72	18499.40	350.39	3-dehydroquinate dehydratase	Lyase	(1r,4s,5r)-1,4,5-trihydroxy-3-[(5-methyl-1-benzothiophen-2-yl)methoxy]cyclohex-2-ene-1-carboxylic acid	CB6	Protein	X-ray	2009-11-24
	C	167	113098.72	18499.40	350.39	3-dehydroquinate dehydratase	Lyase	(1r,4s,5r)-1,4,5-trihydroxy-3-[(5-methyl-1-benzothiophen-2-yl)methoxy]cyclohex-2-ene-1-carboxylic acid	CB6	Protein	X-ray	2009-11-24
	D	167	113098.72	18499.40	350.39	3-dehydroquinate dehydratase	Lyase	(1r,4s,5r)-1,4,5-trihydroxy-3-[(5-methyl-1-benzothiophen-2-yl)methoxy]cyclohex-2-ene-1-carboxylic acid	CB6	Protein	X-ray	2009-11-24
	E	167	113098.72	18499.40	350.39	3-dehydroquinate dehydratase	Lyase	(1r,4s,5r)-1,4,5-trihydroxy-3-[(5-	CB6	Protein	X-ray	2009-11-24

								methyl-1-benzothiophen-2-yl)methoxy]cyclohex-2-ene-1-carboxylic acid				
	F	167	113098.72	18499.40	350.39	3-dehydroquinate dehydratase	Lyase	(1r,4s,5r)-1,4,5-trihydroxy-3-[(5-methyl-1-benzothiophen-2-yl)methoxy]cyclohex-2-ene-1-carboxylic acid	CB6	Protein	X-ray	2009-11-24
**2WLT**	A	332	35740.80	35607.70		L-asparaginase	Hydrolase			Protein	X-ray	2009-11-24
**2WP0**	A	180	68052.84	21039.40	35.45	Hoba	Dnaprotein	Chloride ion	CL	Protein	X-ray	2009-11-17
	A	180	68052.84	21039.40	92.09	Hoba	Dnaprotein	Glycerol	GOL	Protein	X-ray	2009-11-17
	A	180	68052.84	21039.40	22.99	Hoba	Dnaprotein	Sodium ion	NA	Protein	X-ray	2009-11-17
	B	180	68052.84	21039.40	35.45	Hoba	Dnaprotein	Chloride ion	CL	Protein	X-ray	2009-11-17
	B	180	68052.84	21039.40	92.09	Hoba	Dnaprotein	Glycerol	GOL	Protein	X-ray	2009-11-17
	B	180	68052.84	21039.40	22.99	Hoba	Dnaprotein	Sodium ion	NA	Protein	X-ray	2009-11-17
	C	112	68052.84	12632.60		Chromosomalprotein DNAA	Dnaprotein			Protein	X-ray	2009-11-17
	D	112	68052.84	12632.60		Chromosomalprotein DNAA	Dnaprotein			Protein	X-ray	2009-11-17
**2WT4**	A	332	35740.80	35607.70		L-asparaginase	Hydrolase			Protein	X-ray	2009-11-24
**2WVB**	A	148	34270.61	17020.20	46.03	Putative nickel-responsive regulator	Transcription	Formic acid	FMT	Protein	X-ray	2010-01-19
	A	148	34270.61	17020.20	92.09	Putative nickel-responsive regulator	Transcription	Glycerol	GOL	Protein	X-ray	2010-01-19
	B	148	34270.61	17020.20		Putative nickel-responsive regulator	Transcription			Protein	X-ray	2010-01-19
**2WVC**	A	148	34999.18	17008.20	92.09	Putative nickel-responsive regulator	Transcription	Glycerol	GOL	Protein	X-ray	2010-01-19
	A	148	34999.18	17008.20	96.06	Putative nickel-responsive regulator	Transcription	Sulfate ion	SO4	Protein	X-ray	2010-01-19
	B	148	34999.18	17008.20	46.03	Putative nickel-responsive regulator	Transcription	Formic acid	FMT	Protein	X-ray	2010-01-19
	B	148	34999.18	17008.20	92.09	Putative nickel-responsive regulator	Transcription	Glycerol	GOL	Protein	X-ray	2010-01-19
	B	148	34999.18	17008.20	96.06	Putative nickel-responsive regulator	Transcription	Sulfate ion	SO4	Protein	X-ray	2010-01-19
**2WVD**	A	148	69746.12	17154.30	92.09	Putative nickel-responsive regulator	Transcription	Glycerol	GOL	Protein	X-ray	2010-01-19
	A	148	69746.12	17154.30	96.06	Putative nickel-responsive regulator	Transcription	Sulfate ion	SO4	Protein	X-ray	2010-01-19
	B	148	69746.12	17154.30	92.09	Putative nickel-responsive regulator	Transcription	Glycerol	GOL	Protein	X-ray	2010-01-19
	B	148	69746.12	17154.30	96.06	Putative nickel-responsive regulator	Transcription	Sulfate ion	SO4	Protein	X-ray	2010-01-19
	C	148	69746.12	17154.30	92.09	Putative nickel-responsive regulator	Transcription	Glycerol	GOL	Protein	X-ray	2010-01-19
	C	148	69746.12	17154.30	96.06	Putative nickel-responsive regulator	Transcription	Sulfate ion	SO4	Protein	X-ray	2010-01-19

	D	148	69746.12	17154.30	92.09	Putative nickel-responsive regulator	Transcription	Glycerol	GOL	Protein	X-ray	2010-01-19
	D	148	69746.12	17154.30	96.06	Putative nickel-responsive regulator	Transcription	Sulfate ion	SO4	Protein	X-ray	2010-01-19
**2WVE**	A	148	35449.64	17189.40	192.12	Putative nickel-responsive regulator	Transcription	Citric acid	CIT	Protein	X-ray	2010-01-19
	A	148	35449.64	17189.40	92.09	Putative nickel-responsive regulator	Transcription	Glycerol	GOL	Protein	X-ray	2010-01-19
	A	148	35449.64	17189.40	96.06	Putative nickel-responsive regulator	Transcription	Sulfate ion	SO4	Protein	X-ray	2010-01-19
	A	148	35449.64	17189.40	122.14	Putative nickel-responsive regulator	Transcription	2-amino-2-hydroxymethyl-propane-1,3-diol	TRS	Protein	X-ray	2010-01-19
	B	148	35449.64	17189.40	92.09	Putative nickel-responsive regulator	Transcription	Glycerol	GOL	Protein	X-ray	2010-01-19
	B	148	35449.64	17189.40	96.06	Putative nickel-responsive regulator	Transcription	Sulfate ion	SO4	Protein	X-ray	2010-01-19
**2WVF**	A	148	35016.47	17008.20	46.03	Putative nickel-responsive regulator	Transcription	Formic acid	FMT	Protein	X-ray	2010-01-19
	A	148	35016.47	17008.20	92.09	Putative nickel-responsive regulator	Transcription	Glycerol	GOL	Protein	X-ray	2010-01-19
	A	148	35016.47	17008.20	58.70	Putative nickel-responsive regulator	Transcription	Nickel (ii) ion	NI	Protein	X-ray	2010-01-19
	A	148	35016.47	17008.20	96.06	Putative nickel-responsive regulator	Transcription	Sulfate ion	SO4	Protein	X-ray	2010-01-19
	B	148	35016.47	17008.20	46.03	Putative nickel-responsive regulator	Transcription	Formic acid	FMT	Protein	X-ray	2010-01-19
	B	148	35016.47	17008.20	92.09	Putative nickel-responsive regulator	Transcription	Glycerol	GOL	Protein	X-ray	2010-01-19
	B	148	35016.47	17008.20	58.70	Putative nickel-responsive regulator	Transcription	Nickel (ii) ion	NI	Protein	X-ray	2010-01-19
**2XB9**	A	167	56310.93	18499.40	310.30	3-dehydroquinate dehydratase	Lyase	(1r,2r,4s,5r)-1,4,5-trihydroxy-2-(4-methoxybenzyl)-3-oxocyclohexanecarboxylic acid	XNW	Protein	X-ray	2010-09-15
	B	167	56310.93	18499.40	310.30	3-dehydroquinate dehydratase	Lyase	(1r,2r,4s,5r)-1,4,5-trihydroxy-2-(4-methoxybenzyl)-3-oxocyclohexanecarboxylic acid	XNW	Protein	X-ray	2010-09-15
	C	167	56310.93	18499.40	192.12	3-dehydroquinate dehydratase	Lyase	Citric acid	CIT	Protein	X-ray	2010-09-15
**2XD9**	A	167	56309.10	18499.40	270.30	3-dehydroquinate dehydratase	Lyase	(4r,6r,7s)-4,6,7-trihydroxy-2-[(1e)-prop-1-en-1-yl]-4,5,6,7-tetrahydro-1-benzothiophene-4-carboxylic acid	XD9	Protein	X-ray	2010-11-24
	B	167	56309.10	18499.40	270.30	3-dehydroquinate dehydratase	Lyase	(4r,6r,7s)-4,6,7-trihydroxy-2-[(1e)-prop-1-en-1-yl]-4,5,6,7-tetrahydro-1-benzothiophene-4-carboxylic acid	XD9	Protein	X-ray	2010-11-24
	C	167	56309.10	18499.40	270.30	3-dehydroquinate dehydratase	Lyase	(4r,6r,7s)-4,6,7-trihydroxy-2-[(1e)-prop-1-en-1-yl]-4,5,6,7-tetrahydro-1-	XD9	Protein	X-ray	2010-11-24

								benzothiophene-4-carboxylic acid				
**2XDA**	A	167	18797.75	18499.40	298.35	3-dehydroquinate dehydratase	Lyase	(4r,6r,7s)-2-(2-cyclopropylethyl)-4,6,7-trihydroxy-4,5,6,7-tetrahydro-1-benzothiophene-4-carboxylic acid	JPS	Protein	X-ray	2010-11-24
**2XIG**	A	150	71706.68	17682.50	65.38	Ferricprotein	Transcription	Zinc ion	ZN	Protein	X-ray	2011-01-19
	B	150	71706.68	17682.50	192.12	Ferricprotein	Transcription	Citric acid	CIT	Protein	X-ray	2011-01-19
	B	150	71706.68	17682.50	65.38	Ferricprotein	Transcription	Zinc ion	ZN	Protein	X-ray	2011-01-19
	C	150	71706.68	17682.50	65.38	Ferricprotein	Transcription	Zinc ion	ZN	Protein	X-ray	2011-01-19
	D	150	71706.68	17682.50	65.38	Ferricprotein	Transcription	Zinc ion	ZN	Protein	X-ray	2011-01-19
**2XRH**	A	100	11635.31	11512.20	123.11	Protein hp0721	Unknown function	Nicotinic acid	NIO	Protein	X-ray	2011-09-28
**2Y3Y**	A	91	43569.14	10428.90	58.70	Putative nickel-responsive regulator	Transcription	Nickel (ii) ion	NI	Protein	X-ray	2011-08-17
	B	91	43569.14	10428.90	238.30	Putative nickel-responsive regulator	Transcription	4-(2-hydroxyethyl)-1-piperazine ethanesulfonic acid	EPE	Protein	X-ray	2011-08-17
	B	91	43569.14	10428.90	58.70	Putative nickel-responsive regulator	Transcription	Nickel (ii) ion	NI	Protein	X-ray	2011-08-17
	C	91	43569.14	10428.90	238.30	Putative nickel-responsive regulator	Transcription	4-(2-hydroxyethyl)-1-piperazine ethanesulfonic acid	EPE	Protein	X-ray	2011-08-17
	C	91	43569.14	10428.90	58.70	Putative nickel-responsive regulator	Transcription	Nickel (ii) ion	NI	Protein	X-ray	2011-08-17
	C	91	43569.14	10428.90	106.12	Putative nickel-responsive regulator	Transcription	Di(hydroxyethyl)ether	PEG	Protein	X-ray	2011-08-17
	D	91	43569.14	10428.90	58.70	Putative nickel-responsive regulator	Transcription	Nickel (ii) ion	NI	Protein	X-ray	2011-08-17
	Q	11	43569.14	823.77		Undecapeptide-gsssgsasgag	Transcription			Protein	X-ray	2011-08-17
**2ZL0**	A	196	301669.22	21547.80		ATP-dependent Clp protease proteolytic subunit	Hydrolase			Protein	X-ray	2008-04-22
	B	196	301669.22	21547.80		ATP-dependent Clp protease proteolytic subunit	Hydrolase			Protein	X-ray	2008-04-22
	C	196	301669.22	21547.80		ATP-dependent Clp protease proteolytic subunit	Hydrolase			Protein	X-ray	2008-04-22
	D	196	301669.22	21547.80		ATP-dependent Clp protease proteolytic subunit	Hydrolase			Protein	X-ray	2008-04-22
	E	196	301669.22	21547.80		ATP-dependent Clp protease proteolytic subunit	Hydrolase			Protein	X-ray	2008-04-22
	F	196	301669.22	21547.80		ATP-dependent Clp protease	Hydrolase			Protein	X-ray	2008-04-22

					proteolytic subunit					
	G	196	301669.22	21547.80	ATP-dependent Clp protease proteolytic subunit	Hydrolase		Protein	X-ray	2008-04-22
	H	196	301669.22	21547.80	ATP-dependent Clp protease proteolytic subunit	Hydrolase		Protein	X-ray	2008-04-22
	I	196	301669.22	21547.80	ATP-dependent Clp protease proteolytic subunit	Hydrolase		Protein	X-ray	2008-04-22
	J	196	301669.22	21547.80	ATP-dependent Clp protease proteolytic subunit	Hydrolase		Protein	X-ray	2008-04-22
	K	196	301669.22	21547.80	ATP-dependent Clp protease proteolytic subunit	Hydrolase		Protein	X-ray	2008-04-22
	L	196	301669.22	21547.80	ATP-dependent Clp protease proteolytic subunit	Hydrolase		Protein	X-ray	2008-04-22
	M	196	301669.22	21547.80	ATP-dependent Clp protease proteolytic subunit	Hydrolase		Protein	X-ray	2008-04-22
	N	196	301669.22	21547.80	ATP-dependent Clp protease proteolytic subunit	Hydrolase		Protein	X-ray	2008-04-22
**2ZL2**	A	196	309119.72	21547.80	ATP-dependent Clp protease proteolytic subunit	Hydrolase		Protein	X-ray	2008-04-22
	B	196	309119.72	21547.80	ATP-dependent Clp protease proteolytic subunit	Hydrolase		Protein	X-ray	2008-04-22
	C	196	309119.72	21547.80	ATP-dependent Clp protease proteolytic subunit	Hydrolase		Protein	X-ray	2008-04-22
	D	196	309119.72	21547.80	ATP-dependent Clp protease proteolytic subunit	Hydrolase		Protein	X-ray	2008-04-22
	E	196	309119.72	21547.80	ATP-dependent Clp protease proteolytic subunit	Hydrolase		Protein	X-ray	2008-04-22
	F	196	309119.72	21547.80	ATP-dependent Clp protease proteolytic subunit	Hydrolase		Protein	X-ray	2008-04-22
	G	196	309119.72	21547.80	ATP-dependent Clp protease proteolytic subunit	Hydrolase		Protein	X-ray	2008-04-22

	H	196	309119.72	21547.80		ATP-dependent Clp protease proteolytic subunit	Hydrolase			Protein	X-ray	2008-04-22
	I	196	309119.72	21547.80		ATP-dependent Clp protease proteolytic subunit	Hydrolase			Protein	X-ray	2008-04-22
	J	196	309119.72	21547.80		ATP-dependent Clp protease proteolytic subunit	Hydrolase			Protein	X-ray	2008-04-22
	K	196	309119.72	21547.80		ATP-dependent Clp protease proteolytic subunit	Hydrolase			Protein	X-ray	2008-04-22
	L	196	309119.72	21547.80		ATP-dependent Clp protease proteolytic subunit	Hydrolase			Protein	X-ray	2008-04-22
	M	196	309119.72	21547.80		ATP-dependent Clp protease proteolytic subunit	Hydrolase			Protein	X-ray	2008-04-22
	N	196	309119.72	21547.80		ATP-dependent Clp protease proteolytic subunit	Hydrolase			Protein	X-ray	2008-04-22
	O	7	309119.72	777.87		A peptide substrate-NVLGFTQ	Hydrolase			Protein	X-ray	2008-04-22
	P	7	309119.72	777.87		A peptide substrate-NVLGFTQ	Hydrolase			Protein	X-ray	2008-04-22
	Q	7	309119.72	777.87		A peptide substrate-NVLGFTQ	Hydrolase			Protein	X-ray	2008-04-22
	R	7	309119.72	613.75	103.12	A peptide substrate-NVLGFTQ for Chain R and S	Hydrolase	Unknown	UNK	Protein	X-ray	2008-04-22
	S	7	309119.72	613.75	103.12	A peptide substrate-NVLGFTQ for Chain R and S	Hydrolase	Unknown	UNK	Protein	X-ray	2008-04-22
	T	7	309119.72	777.87		A peptide substrate-NVLGFTQ	Hydrolase			Protein	X-ray	2008-04-22
	U	7	309119.72	777.87		A peptide substrate-NVLGFTQ	Hydrolase			Protein	X-ray	2008-04-22
	V	7	309119.72	777.87		A peptide substrate-NVLGFTQ	Hydrolase			Protein	X-ray	2008-04-22
	W	7	309119.72	777.87		A peptide substrate-NVLGFTQ	Hydrolase			Protein	X-ray	2008-04-22
	X	7	309119.72	777.87		A peptide substrate-NVLGFTQ	Hydrolase			Protein	X-ray	2008-04-22
**2ZL3**	A	196	301445.22	21531.80		ATP-dependent Clp protease proteolytic subunit	Hydrolase			Protein	X-ray	2008-04-22
	B	196	301445.22	21531.80		ATP-dependent Clp protease proteolytic subunit	Hydrolase			Protein	X-ray	2008-04-22
	C	196	301445.22	21531.80		ATP-dependent Clp protease	Hydrolase			Protein	X-ray	2008-04-22

					proteolytic subunit				
	D	196	301445.22	21531.80	ATP-dependent Clp protease proteolytic subunit	Hydrolase	Protein	X-ray	2008-04-22
	E	196	301445.22	21531.80	ATP-dependent Clp protease proteolytic subunit	Hydrolase	Protein	X-ray	2008-04-22
	F	196	301445.22	21531.80	ATP-dependent Clp protease proteolytic subunit	Hydrolase	Protein	X-ray	2008-04-22
	G	196	301445.22	21531.80	ATP-dependent Clp protease proteolytic subunit	Hydrolase	Protein	X-ray	2008-04-22
	H	196	301445.22	21531.80	ATP-dependent Clp protease proteolytic subunit	Hydrolase	Protein	X-ray	2008-04-22
	I	196	301445.22	21531.80	ATP-dependent Clp protease proteolytic subunit	Hydrolase	Protein	X-ray	2008-04-22
	J	196	301445.22	21531.80	ATP-dependent Clp protease proteolytic subunit	Hydrolase	Protein	X-ray	2008-04-22
	K	196	301445.22	21531.80	ATP-dependent Clp protease proteolytic subunit	Hydrolase	Protein	X-ray	2008-04-22
	L	196	301445.22	21531.80	ATP-dependent Clp protease proteolytic subunit	Hydrolase	Protein	X-ray	2008-04-22
	M	196	301445.22	21531.80	ATP-dependent Clp protease proteolytic subunit	Hydrolase	Protein	X-ray	2008-04-22
	N	196	301445.22	21531.80	ATP-dependent Clp protease proteolytic subunit	Hydrolase	Protein	X-ray	2008-04-22
**2ZL4**	1	4	305677.84	302.33	Peptide substrate AAAA	Hydrolase	Protein	X-ray	2008-04-22
**2ZL4**	2	4	305677.84	302.33	Peptide substrate AAAA	Hydrolase	Protein	X-ray	2008-04-22
**2ZL4**	A	196	305677.84	21531.80	ATP-dependent Clp protease proteolytic subunit	Hydrolase	Protein	X-ray	2008-04-22
	B	196	305677.84	21531.80	ATP-dependent Clp protease proteolytic subunit	Hydrolase	Protein	X-ray	2008-04-22
	C	196	305677.84	21531.80	ATP-dependent Clp protease proteolytic subunit	Hydrolase	Protein	X-ray	2008-04-22

D	196	305677.84	21531.80	ATP-dependent Clp protease proteolytic subunit	Hydrolase		Protein	X-ray	2008-04-22
E	196	305677.84	21531.80	ATP-dependent Clp protease proteolytic subunit	Hydrolase		Protein	X-ray	2008-04-22
F	196	305677.84	21531.80	ATP-dependent Clp protease proteolytic subunit	Hydrolase		Protein	X-ray	2008-04-22
G	196	305677.84	21531.80	ATP-dependent Clp protease proteolytic subunit	Hydrolase		Protein	X-ray	2008-04-22
H	196	305677.84	21531.80	ATP-dependent Clp protease proteolytic subunit	Hydrolase		Protein	X-ray	2008-04-22
I	196	305677.84	21531.80	ATP-dependent Clp protease proteolytic subunit	Hydrolase		Protein	X-ray	2008-04-22
J	196	305677.84	21531.80	ATP-dependent Clp protease proteolytic subunit	Hydrolase		Protein	X-ray	2008-04-22
K	196	305677.84	21531.80	ATP-dependent Clp protease proteolytic subunit	Hydrolase		Protein	X-ray	2008-04-22
L	196	305677.84	21531.80	ATP-dependent Clp protease proteolytic subunit	Hydrolase		Protein	X-ray	2008-04-22
M	196	305677.84	21531.80	ATP-dependent Clp protease proteolytic subunit	Hydrolase		Protein	X-ray	2008-04-22
N	196	305677.84	21531.80	ATP-dependent Clp protease proteolytic subunit	Hydrolase		Protein	X-ray	2008-04-22
O	4	305677.84	302.33	Peptide substrate AAAA	Hydrolase		Protein	X-ray	2008-04-22
P	4	305677.84	302.33	Peptide substrate AAAA	Hydrolase		Protein	X-ray	2008-04-22
Q	4	305677.84	302.33	Peptide substrate AAAA	Hydrolase		Protein	X-ray	2008-04-22
R	4	305677.84	302.33	Peptide substrate AAAA	Hydrolase		Protein	X-ray	2008-04-22
S	4	305677.84	302.33	Peptide substrate AAAA	Hydrolase		Protein	X-ray	2008-04-22
T	4	305677.84	302.33	Peptide substrate AAAA	Hydrolase		Protein	X-ray	2008-04-22
U	4	305677.84	302.33	Peptide substrate AAAA	Hydrolase		Protein	X-ray	2008-04-22
V	4	305677.84	302.33	Peptide substrate AAAA	Hydrolase		Protein	X-ray	2008-04-22
W	4	305677.84	302.33	Peptide substrate AAAA	Hydrolase		Protein	X-ray	2008-04-22

	X	4	305677.84	302.33		Peptide substrate AAAA	Hydrolase			Protein	X-ray	2008-04-22
	Y	4	305677.84	302.33		Peptide substrate AAAA	Hydrolase			Protein	X-ray	2008-04-22
	Z	4	305677.84	302.33		Peptide substrate AAAA	Hydrolase			Protein	X-ray	2008-04-22
**3AH5**	A	216	158692.48	25178.90	785.56	Thymidylate synthase thyx	Transferase	Flavin-adenine dinucleotide	FAD	Protein	X-ray	2011-04-20
	A	216	158692.48	25178.90	96.06	Thymidylate synthase thyx	Transferase	Sulfate ion	SO4	Protein	X-ray	2011-04-20
	A	216	158692.48	25178.90	308.18	Thymidylate synthase thyx	Transferase	2'-deoxyuridine 5'-monophosphate	UMP	Protein	X-ray	2011-04-20
	B	216	158692.48	25178.90	785.56	Thymidylate synthase thyx	Transferase	Flavin-adenine dinucleotide	FAD	Protein	X-ray	2011-04-20
	B	216	158692.48	25178.90	308.18	Thymidylate synthase thyx	Transferase	2'-deoxyuridine 5'-monophosphate	UMP	Protein	X-ray	2011-04-20
	C	216	158692.48	25178.90	785.56	Thymidylate synthase thyx	Transferase	Flavin-adenine dinucleotide	FAD	Protein	X-ray	2011-04-20
	C	216	158692.48	25178.90	96.06	Thymidylate synthase thyx	Transferase	Sulfate ion	SO4	Protein	X-ray	2011-04-20
	C	216	158692.48	25178.90	308.18	Thymidylate synthase thyx	Transferase	2'-deoxyuridine 5'-monophosphate	UMP	Protein	X-ray	2011-04-20
	D	216	158692.48	25178.90	785.56	Thymidylate synthase thyx	Transferase	Flavin-adenine dinucleotide	FAD	Protein	X-ray	2011-04-20
	D	216	158692.48	25178.90	96.06	Thymidylate synthase thyx	Transferase	Sulfate ion	SO4	Protein	X-ray	2011-04-20
	D	216	158692.48	25178.90	308.18	Thymidylate synthase thyx	Transferase	2'-deoxyuridine 5'-monophosphate	UMP	Protein	X-ray	2011-04-20
	E	216	158692.48	25178.90	785.56	Thymidylate synthase thyx	Transferase	Flavin-adenine dinucleotide	FAD	Protein	X-ray	2011-04-20
	E	216	158692.48	25178.90	96.06	Thymidylate synthase thyx	Transferase	Sulfate ion	SO4	Protein	X-ray	2011-04-20
	E	216	158692.48	25178.90	308.18	Thymidylate synthase thyx	Transferase	2'-deoxyuridine 5'-monophosphate	UMP	Protein	X-ray	2011-04-20
	F	216	158692.48	25178.90	785.56	Thymidylate synthase thyx	Transferase	Flavin-adenine dinucleotide	FAD	Protein	X-ray	2011-04-20
	F	216	158692.48	25178.90	96.06	Thymidylate synthase thyx	Transferase	Sulfate ion	SO4	Protein	X-ray	2011-04-20
	F	216	158692.48	25178.90	308.18	Thymidylate synthase thyx	Transferase	2'-deoxyuridine 5'-monophosphate	UMP	Protein	X-ray	2011-04-20
**3AKJ**	A	325	75308.00	37654.00		Ctka	Transferase			Protein	X-ray	2010-12-01
	B	325	75308.00	37654.00		Ctka	Transferase			Protein	X-ray	2010-12-01
**3AKK**	A	325	152422.03	37654.00	427.20	Ctka	Transferase	Adenosine-5'-diphosphate	ADP	Protein	X-ray	2010-12-01
	A	325	152422.03	37654.00	24.31	Ctka	Transferase	Magnesium ion	MG	Protein	X-ray	2010-12-01
	B	325	152422.03	37654.00	427.20	Ctka	Transferase	Adenosine-5'-diphosphate	ADP	Protein	X-ray	2010-12-01
	B	325	152422.03	37654.00	24.31	Ctka	Transferase	Magnesium ion	MG	Protein	X-ray	2010-12-01
	C	325	152422.03	37654.00	427.20	Ctka	Transferase	Adenosine-5'-diphosphate	ADP	Protein	X-ray	2010-12-01
	C	325	152422.03	37654.00	24.31	Ctka	Transferase	Magnesium ion	MG	Protein	X-ray	2010-12-01
	D	325	152422.03	37654.00	427.20	Ctka	Transferase	Adenosine-5'-diphosphate	ADP	Protein	X-ray	2010-12-01
	D	325	152422.03	37654.00	24.31	Ctka	Transferase	Magnesium ion	MG	Protein	X-ray	2010-12-01
**3AKL**	A	325	152731.94	37654.00	506.20	Ctka	Transferase	Phosphoaminophosphonic acid-	ANP	Protein	X-ray	2010-12-01

								adenylate ester				
	A	325	152731.94	37654.00	24.31	Ctka	Transferase	Magnesium ion	MG	Protein	X-ray	2010-12-01
	B	325	152731.94	37654.00	506.20	Ctka	Transferase	Phosphoaminophosphonic acid-adenylate ester	ANP	Protein	X-ray	2010-12-01
	B	325	152731.94	37654.00	24.31	Ctka	Transferase	Magnesium ion	MG	Protein	X-ray	2010-12-01
	C	325	152731.94	37654.00	427.20	Ctka	Transferase	Adenosine-5'-diphosphate	ADP	Protein	X-ray	2010-12-01
	C	325	152731.94	37654.00	24.31	Ctka	Transferase	Magnesium ion	MG	Protein	X-ray	2010-12-01
	D	325	152731.94	37654.00	506.20	Ctka	Transferase	Phosphoaminophosphonic acid-adenylate ester	ANP	Protein	X-ray	2010-12-01
	D	325	152731.94	37654.00	24.31	Ctka	Transferase	Magnesium ion	MG	Protein	X-ray	2010-12-01
**3B7J**	A	159	110639.91	18206.30	120.15	(3R)-hydroxymyristoyl-acylprotein dehydratase	Lyase	Benzamidine	BEN	Protein	X-ray	2008-09-02
	A	159	110639.91	18206.30	35.45	(3R)-hydroxymyristoyl-acylprotein dehydratase	Lyase	Chloride ion	CL	Protein	X-ray	2008-09-02
	B	159	110639.91	18206.30	120.15	(3R)-hydroxymyristoyl-acylprotein dehydratase	Lyase	Benzamidine	BEN	Protein	X-ray	2008-09-02
	B	159	110639.91	18206.30	35.45	(3R)-hydroxymyristoyl-acylprotein dehydratase	Lyase	Chloride ion	CL	Protein	X-ray	2008-09-02
	B	159	110639.91	18206.30	174.16	(3R)-hydroxymyristoyl-acylprotein dehydratase	Lyase	5-hydroxynaphthalene-1,4-dione	JUG	Protein	X-ray	2008-09-02
	C	159	110639.91	18206.30	120.15	(3R)-hydroxymyristoyl-acylprotein dehydratase	Lyase	Benzamidine	BEN	Protein	X-ray	2008-09-02
	C	159	110639.91	18206.30	35.45	(3R)-hydroxymyristoyl-acylprotein dehydratase	Lyase	Chloride ion	CL	Protein	X-ray	2008-09-02
	C	159	110639.91	18206.30	174.16	(3R)-hydroxymyristoyl-acylprotein dehydratase	Lyase	5-hydroxynaphthalene-1,4-dione	JUG	Protein	X-ray	2008-09-02
	D	159	110639.91	18206.30	35.45	(3R)-hydroxymyristoyl-acylprotein dehydratase	Lyase	Chloride ion	CL	Protein	X-ray	2008-09-02
	E	159	110639.91	18206.30	120.15	(3R)-hydroxymyristoyl-acylprotein dehydratase	Lyase	Benzamidine	BEN	Protein	X-ray	2008-09-02
	E	159	110639.91	18206.30	35.45	(3R)-hydroxymyristoyl-acylprotein	Lyase	Chloride ion	CL	Protein	X-ray	2008-09-02

						dehydratase							
	F	159	110639.91	18206.30	120.15	(3R)-hydroxymyristoyl-acylprotein dehydratase	Lyase	Benzamidine	BEN	Protein	X-ray	2008-09-02	
	F	159	110639.91	18206.30	35.45	(3R)-hydroxymyristoyl-acylprotein dehydratase	Lyase	Chloride ion	CL	Protein	X-ray	2008-09-02	
**3BGH**	A	236	55233.49	27376.60	96.06	Putative neuraminyllactose-binding hemagglutinin homolog	SG, unknown function	Sulfate ion	SO4	Protein	X-ray	2007-12-11	NYSRCSG
	B	236	55233.49	27376.60	96.06	Putative neuraminyllactose-binding hemagglutinin homolog	SG, unknown function	Sulfate ion	SO4	Protein	X-ray	2007-12-11	NYSRCSG
**3BVE**	A	181	126021.88	20957.60		Ferritin	Oxidoreductase			Protein	X-ray	2009-01-13	
	B	181	126021.88	20957.60	92.09	Ferritin	Oxidoreductase	Glycerol	GOL	Protein	X-ray	2009-01-13	
	C	181	126021.88	20957.60	92.09	Ferritin	Oxidoreductase	Glycerol	GOL	Protein	X-ray	2009-01-13	
	D	181	126021.88	20957.60		Ferritin	Oxidoreductase			Protein	X-ray	2009-01-13	
	E	181	126021.88	20957.60		Ferritin	Oxidoreductase			Protein	X-ray	2009-01-13	
	F	181	126021.88	20957.60	92.09	Ferritin	Oxidoreductase	Glycerol	GOL	Protein	X-ray	2009-01-13	
**3BVF**	A	181	127580.73	20957.60	55.85	Ferritin	Oxidoreductase	Fe (iii) ion	FE	Protein	X-ray	2009-01-13	
	A	181	127580.73	20957.60	60.10	Ferritin	Oxidoreductase	Isopropyl alcohol	IPA	Protein	X-ray	2009-01-13	
	B	181	127580.73	20957.60	55.85	Ferritin	Oxidoreductase	Fe (iii) ion	FE	Protein	X-ray	2009-01-13	
	B	181	127580.73	20957.60	60.10	Ferritin	Oxidoreductase	Isopropyl alcohol	IPA	Protein	X-ray	2009-01-13	
	C	181	127580.73	20957.60	55.85	Ferritin	Oxidoreductase	Fe (iii) ion	FE	Protein	X-ray	2009-01-13	
	C	181	127580.73	20957.60	60.10	Ferritin	Oxidoreductase	Isopropyl alcohol	IPA	Protein	X-ray	2009-01-13	
	D	181	127580.73	20957.60	55.85	Ferritin	Oxidoreductase	Fe (iii) ion	FE	Protein	X-ray	2009-01-13	
	D	181	127580.73	20957.60	92.09	Ferritin	Oxidoreductase	Glycerol	GOL	Protein	X-ray	2009-01-13	
	D	181	127580.73	20957.60	60.10	Ferritin	Oxidoreductase	Isopropyl alcohol	IPA	Protein	X-ray	2009-01-13	
	E	181	127580.73	20957.60	55.85	Ferritin	Oxidoreductase	Fe (iii) ion	FE	Protein	X-ray	2009-01-13	
	E	181	127580.73	20957.60	92.09	Ferritin	Oxidoreductase	Glycerol	GOL	Protein	X-ray	2009-01-13	
	E	181	127580.73	20957.60	60.10	Ferritin	Oxidoreductase	Isopropyl alcohol	IPA	Protein	X-ray	2009-01-13	
	F	181	127580.73	20957.60	55.85	Ferritin	Oxidoreductase	Fe (iii) ion	FE	Protein	X-ray	2009-01-13	
	F	181	127580.73	20957.60	92.09	Ferritin	Oxidoreductase	Glycerol	GOL	Protein	X-ray	2009-01-13	
	F	181	127580.73	20957.60	60.10	Ferritin	Oxidoreductase	Isopropyl alcohol	IPA	Protein	X-ray	2009-01-13	
**3BVI**	A	181	126859.58	20957.60	55.85	Ferritin	Oxidoreductase	Fe (iii) ion	FE	Protein	X-ray	2009-01-13	

	B	181	126859.58	20957.60	55.85	Ferritin	Oxidoreductase	Fe (iii) ion	FE	Protein	X-ray	2009-01-13
	C	181	126859.58	20957.60	55.85	Ferritin	Oxidoreductase	Fe (iii) ion	FE	Protein	X-ray	2009-01-13
	D	181	126859.58	20957.60	55.85	Ferritin	Oxidoreductase	Fe (iii) ion	FE	Protein	X-ray	2009-01-13
	D	181	126859.58	20957.60	92.09	Ferritin	Oxidoreductase	Glycerol	GOL	Protein	X-ray	2009-01-13
	E	181	126859.58	20957.60	55.85	Ferritin	Oxidoreductase	Fe (iii) ion	FE	Protein	X-ray	2009-01-13
	E	181	126859.58	20957.60	92.09	Ferritin	Oxidoreductase	Glycerol	GOL	Protein	X-ray	2009-01-13
	F	181	126859.58	20957.60	55.85	Ferritin	Oxidoreductase	Fe (iii) ion	FE	Protein	X-ray	2009-01-13
**3BVK**	A	181	126803.73	20957.60	55.85	Ferritin	Oxidoreductase	Fe (iii) ion	FE	Protein	X-ray	2009-01-13
	B	181	126803.73	20957.60	55.85	Ferritin	Oxidoreductase	Fe (iii) ion	FE	Protein	X-ray	2009-01-13
	B	181	126803.73	20957.60	92.09	Ferritin	Oxidoreductase	Glycerol	GOL	Protein	X-ray	2009-01-13
	C	181	126803.73	20957.60	55.85	Ferritin	Oxidoreductase	Fe (iii) ion	FE	Protein	X-ray	2009-01-13
	C	181	126803.73	20957.60	92.09	Ferritin	Oxidoreductase	Glycerol	GOL	Protein	X-ray	2009-01-13
	D	181	126803.73	20957.60	55.85	Ferritin	Oxidoreductase	Fe (iii) ion	FE	Protein	X-ray	2009-01-13
	E	181	126803.73	20957.60	55.85	Ferritin	Oxidoreductase	Fe (iii) ion	FE	Protein	X-ray	2009-01-13
	F	181	126803.73	20957.60	55.85	Ferritin	Oxidoreductase	Fe (iii) ion	FE	Protein	X-ray	2009-01-13
	F	181	126803.73	20957.60	92.09	Ferritin	Oxidoreductase	Glycerol	GOL	Protein	X-ray	2009-01-13
**3BVL**	A	181	126915.43	20957.60	55.85	Ferritin	Oxidoreductase	Fe (iii) ion	FE	Protein	X-ray	2009-01-13
	B	181	126915.43	20957.60	55.85	Ferritin	Oxidoreductase	Fe (iii) ion	FE	Protein	X-ray	2009-01-13
	B	181	126915.43	20957.60	92.09	Ferritin	Oxidoreductase	Glycerol	GOL	Protein	X-ray	2009-01-13
	C	181	126915.43	20957.60	55.85	Ferritin	Oxidoreductase	Fe (iii) ion	FE	Protein	X-ray	2009-01-13
	D	181	126915.43	20957.60	55.85	Ferritin	Oxidoreductase	Fe (iii) ion	FE	Protein	X-ray	2009-01-13
	D	181	126915.43	20957.60	92.09	Ferritin	Oxidoreductase	Glycerol	GOL	Protein	X-ray	2009-01-13
	E	181	126915.43	20957.60	55.85	Ferritin	Oxidoreductase	Fe (iii) ion	FE	Protein	X-ray	2009-01-13
	F	181	126915.43	20957.60	55.85	Ferritin	Oxidoreductase	Fe (iii) ion	FE	Protein	X-ray	2009-01-13
	F	181	126915.43	20957.60	92.09	Ferritin	Oxidoreductase	Glycerol	GOL	Protein	X-ray	2009-01-13
**3C4U**	A	307	67778.94	33801.10	22.99	Fructose-bisphosphate aldolase	Lyase	Sodium ion	NA	Protein	X-ray	2008-08-26
	A	307	67778.94	33801.10	65.38	Fructose-bisphosphate aldolase	Lyase	Zinc ion	ZN	Protein	X-ray	2008-08-26
	B	307	67778.94	33801.10	22.99	Fructose-bisphosphate aldolase	Lyase	Sodium ion	NA	Protein	X-ray	2008-08-26
	B	307	67778.94	33801.10	65.38	Fructose-bisphosphate aldolase	Lyase	Zinc ion	ZN	Protein	X-ray	2008-08-26
**3C52**	A	307	68201.20	33801.10	40.08	Fructose-bisphosphate aldolase	Lyase	Calcium ion	CA	Protein	X-ray	2008-08-26
	A	307	68201.20	33801.10	22.99	Fructose-bisphosphate aldolase	Lyase	Sodium ion	NA	Protein	X-ray	2008-08-26

	A	307	68201.20	33801.10	171.05	Fructose-bisphosphate aldolase	Lyase	Phosphoglycolohydroxamic acid	PGH	Protein	X-ray	2008-08-26
	A	307	68201.20	33801.10	65.38	Fructose-bisphosphate aldolase	Lyase	Zinc ion	ZN	Protein	X-ray	2008-08-26
	B	307	68201.20	33801.10	40.08	Fructose-bisphosphate aldolase	Lyase	Calcium ion	CA	Protein	X-ray	2008-08-26
	B	307	68201.20	33801.10	22.99	Fructose-bisphosphate aldolase	Lyase	Sodium ion	NA	Protein	X-ray	2008-08-26
	B	307	68201.20	33801.10	171.05	Fructose-bisphosphate aldolase	Lyase	Phosphoglycolohydroxamic acid	PGH	Protein	X-ray	2008-08-26
	B	307	68201.20	33801.10	65.38	Fructose-bisphosphate aldolase	Lyase	Zinc ion	ZN	Protein	X-ray	2008-08-26
**3C56**	A	307	68351.18	33801.10	309.11	Fructose-bisphosphate aldolase	Lyase	3-(hydroxy[(phosphonooxy)acetyl]amino}propyl dihydrogen phosphate	PH4	Protein	X-ray	2008-08-26
	A	307	68351.18	33801.10	65.38	Fructose-bisphosphate aldolase	Lyase	Zinc ion	ZN	Protein	X-ray	2008-08-26
	B	307	68351.18	33801.10	309.11	Fructose-bisphosphate aldolase	Lyase	3-(hydroxy[(phosphonooxy)acetyl]amino}propyl dihydrogen phosphate	PH4	Protein	X-ray	2008-08-26
	B	307	68351.18	33801.10	65.38	Fructose-bisphosphate aldolase	Lyase	Zinc ion	ZN	Protein	X-ray	2008-08-26
**3C5Q**	A	425	47960.63	47474.20	92.09	Diaminopimelate decarboxylase	Lyase	Glycerol	GOL	Protein	X-ray	2008-05-27
	A	425	47960.63	47474.20	247.14	Diaminopimelate decarboxylase	Lyase	Pyridoxal-5'-phosphate	PLP	Protein	X-ray	2008-05-27
**3CEI**	A	213	49303.55	24547.90	55.85	Superoxide dismutase	Oxidoreductase	Fe (iii) ion	FE	Protein	X-ray	2008-06-10
	A	213	49303.55	24547.90	96.06	Superoxide dismutase	Oxidoreductase	Sulfate ion	SO4	Protein	X-ray	2008-06-10
	B	213	49303.55	24547.90	55.85	Superoxide dismutase	Oxidoreductase	Fe (iii) ion	FE	Protein	X-ray	2008-06-10
**3CF8**	A	159	110775.93	18206.30	120.15	(3R)-hydroxymyristoyl-acylprotein dehydratase	Lyase	Benzamidine	BEN	Protein	X-ray	2008-12-09
	A	159	110775.93	18206.30	35.45	(3R)-hydroxymyristoyl-acylprotein dehydratase	Lyase	Chloride ion	CL	Protein	X-ray	2008-12-09
	A	159	110775.93	18206.30	302.24	(3R)-hydroxymyristoyl-acylprotein dehydratase	Lyase	3,5,7,3',4'-pentahydroxyflavone	QUE	Protein	X-ray	2008-12-09
	B	159	110775.93	18206.30	120.15	(3R)-hydroxymyristoyl-acylprotein dehydratase	Lyase	Benzamidine	BEN	Protein	X-ray	2008-12-09
	B	159	110775.93	18206.30	35.45	(3R)-hydroxymyristoyl-acylprotein dehydratase	Lyase	Chloride ion	CL	Protein	X-ray	2008-12-09
	C	159	110775.93	18206.30	120.15	(3R)-hydroxymyristoyl-acylprotein dehydratase	Lyase	Benzamidine	BEN	Protein	X-ray	2008-12-09

	C	159	110775.93	18206.30	35.45	(3R)-hydroxymyristoyl-acylprotein dehydratase	Lyase	Chloride ion	CL	Protein	X-ray	2008-12-09
	C	159	110775.93	18206.30	302.24	(3R)-hydroxymyristoyl-acylprotein dehydratase	Lyase	3,5,7,3',4'-pentahydroxyflavone	QUE	Protein	X-ray	2008-12-09
	D	159	110775.93	18206.30	35.45	(3R)-hydroxymyristoyl-acylprotein dehydratase	Lyase	Chloride ion	CL	Protein	X-ray	2008-12-09
	E	159	110775.93	18206.30	35.45	(3R)-hydroxymyristoyl-acylprotein dehydratase	Lyase	Chloride ion	CL	Protein	X-ray	2008-12-09
	F	159	110775.93	18206.30	35.45	(3R)-hydroxymyristoyl-acylprotein dehydratase	Lyase	Chloride ion	CL	Protein	X-ray	2008-12-09
**3CF9**	A	159	110471.62	18206.30	270.24	(3R)-hydroxymyristoyl-acylprotein dehydratase	Lyase	5,7-dihydroxy-2-(4-hydroxyphenyl)-4h-chromen-4-one	AGI	Protein	X-ray	2008-12-09
	A	159	110471.62	18206.30	35.45	(3R)-hydroxymyristoyl-acylprotein dehydratase	Lyase	Chloride ion	CL	Protein	X-ray	2008-12-09
	B	159	110471.62	18206.30	120.15	(3R)-hydroxymyristoyl-acylprotein dehydratase	Lyase	Benzamidine	BEN	Protein	X-ray	2008-12-09
	B	159	110471.62	18206.30	35.45	(3R)-hydroxymyristoyl-acylprotein dehydratase	Lyase	Chloride ion	CL	Protein	X-ray	2008-12-09
	C	159	110471.62	18206.30	270.24	(3R)-hydroxymyristoyl-acylprotein dehydratase	Lyase	5,7-dihydroxy-2-(4-hydroxyphenyl)-4h-chromen-4-one	AGI	Protein	X-ray	2008-12-09
	C	159	110471.62	18206.30	120.15	(3R)-hydroxymyristoyl-acylprotein dehydratase	Lyase	Benzamidine	BEN	Protein	X-ray	2008-12-09
	C	159	110471.62	18206.30	35.45	(3R)-hydroxymyristoyl-acylprotein dehydratase	Lyase	Chloride ion	CL	Protein	X-ray	2008-12-09
	D	159	110471.62	18206.30	35.45	(3R)-hydroxymyristoyl-acylprotein dehydratase	Lyase	Chloride ion	CL	Protein	X-ray	2008-12-09
	E	159	110471.62	18206.30	120.15	(3R)-hydroxymyristoyl-acylprotein dehydratase	Lyase	Benzamidine	BEN	Protein	X-ray	2008-12-09
	E	159	110471.62	18206.30	35.45	(3R)-hydroxymyristoyl-acylprotein dehydratase	Lyase	Chloride ion	CL	Protein	X-ray	2008-12-09
	F	159	110471.62	18206.30	35.45	(3R)-hydroxymyristoyl-acylprotein	Lyase	Chloride ion	CL	Protein	X-ray	2008-12-09

						dehydratase						
**3CLH**	A	343	79810.02	39176.20	663.43	3-dehydroquinate synthase	Lyase	Nicotinamide-adenine-dinucleotide	NAD	Protein	X-ray	2009-03-24
	A	343	79810.02	39176.20	65.38	3-dehydroquinate synthase	Lyase	Zinc ion	ZN	Protein	X-ray	2009-03-24
	B	343	79810.02	39176.20	663.43	3-dehydroquinate synthase	Lyase	Nicotinamide-adenine-dinucleotide	NAD	Protein	X-ray	2009-03-24
	B	343	79810.02	39176.20	65.38	3-dehydroquinate synthase	Lyase	Zinc ion	ZN	Protein	X-ray	2009-03-24
**3CWX**	A	176	62332.80	20777.60	196.11	Protein cagd	Unknown function	Selenomethionine	MSE	Protein	X-ray	2008-12-30
	B	176	62332.80	20777.60	196.11	Protein cagd	Unknown function	Selenomethionine	MSE	Protein	X-ray	2008-12-30
	C	176	62332.80	20777.60	196.11	Protein cagd	Unknown function	Selenomethionine	MSE	Protein	X-ray	2008-12-30
**3CWY**	A	176	20841.15	20777.60	63.55	Protein cagd	Unknown function	Copper (ii) ion	CU	Protein	X-ray	2008-12-30
	A	176	20841.15	20777.60	196.11	Protein cagd	Unknown function	Selenomethionine	MSE	Protein	X-ray	2008-12-30
**3CXN**	A	274	94110.00	31339.30	196.11	Ureaseprotein uref	Chaperone	Selenomethionine	MSE	Protein	X-ray	2009-05-12
	B	274	94110.00	31339.30	92.09	Ureaseprotein uref	Chaperone	Glycerol	GOL	Protein	X-ray	2009-05-12
	B	274	94110.00	31339.30	196.11	Ureaseprotein uref	Chaperone	Selenomethionine	MSE	Protein	X-ray	2009-05-12
	C	274	94110.00	31339.30	196.11	Ureaseprotein uref	Chaperone	Selenomethionine	MSE	Protein	X-ray	2009-05-12
	B	138	63760.40	15940.10		Chemotaxis protein motb	Membrane protein			Protein	X-ray	2008-07-08
	C	138	63760.40	15940.10		Chemotaxis protein motb	Membrane protein			Protein	X-ray	2008-07-08
	D	138	63760.40	15940.10		Chemotaxis protein motb	Membrane protein			Protein	X-ray	2008-07-08
	E	138	63760.40	15940.10		Chemotaxis protein motb	Membrane protein			Protein	X-ray	2008-07-08
**3CYQ**	A	138	255628.14	15940.10		Chemotaxis protein motb	Membrane protein			Protein	X-ray	2008-07-08
	B	138	255628.14	15940.10		Chemotaxis protein motb	Membrane protein			Protein	X-ray	2008-07-08
	C	138	255628.14	15940.10		Chemotaxis protein motb	Membrane protein			Protein	X-ray	2008-07-08
	D	138	255628.14	15940.10	293.27	Chemotaxis protein motb	Membrane protein	Beta-n-acetylmuramic acid	AMU	Protein	X-ray	2008-07-08
	E	138	255628.14	15940.10		Chemotaxis protein motb	Membrane protein			Protein	X-ray	2008-07-08
	F	138	255628.14	15940.10		Chemotaxis protein motb	Membrane protein			Protein	X-ray	2008-07-08
	G	138	255628.14	15940.10		Chemotaxis protein motb	Membrane protein			Protein	X-ray	2008-07-08
	H	138	255628.14	15940.10		Chemotaxis protein motb	Membrane protein			Protein	X-ray	2008-07-08
	I	138	255628.14	15940.10		Chemotaxis protein motb	Membrane protein			Protein	X-ray	2008-07-08
	J	138	255628.14	15940.10		Chemotaxis protein motb	Membrane protein			Protein	X-ray	2008-07-08
	K	138	255628.14	15940.10	293.27	Chemotaxis protein motb	Membrane protein	Beta-n-acetylmuramic acid	AMU	Protein	X-ray	2008-07-08
	L	138	255628.14	15940.10		Chemotaxis protein motb	Membrane protein			Protein	X-ray	2008-07-08
	M	138	255628.14	15940.10		Chemotaxis protein motb	Membrane protein			Protein	X-ray	2008-07-08

	N	138	255628.14	15940.10		Chemotaxis protein motb	Membrane protein			Protein	X-ray	2008-07-08
	O	138	255628.14	15940.10		Chemotaxis protein motb	Membrane protein			Protein	X-ray	2008-07-08
	P	138	255628.14	15940.10		Chemotaxis protein motb	Membrane protein			Protein	X-ray	2008-07-08
**3D04**	A	159	110744.01	18206.30	120.15	(3R)-hydroxymyristoyl-acylprotein dehydratase	Lyase	Benzamidine	BEN	Protein	X-ray	2008-12-09
	A	159	110744.01	18206.30	35.45	(3R)-hydroxymyristoyl-acylprotein dehydratase	Lyase	Chloride ion	CL	Protein	X-ray	2008-12-09
	A	159	110744.01	18206.30	286.28	(3R)-hydroxymyristoyl-acylprotein dehydratase	Lyase	(2s)-5-hydroxy-2-(4-hydroxyphenyl)-7-methoxy-2,3-dihydro-4h-chromen-4-one	SAK	Protein	X-ray	2008-12-09
	B	159	110744.01	18206.30	35.45	(3R)-hydroxymyristoyl-acylprotein dehydratase	Lyase	Chloride ion	CL	Protein	X-ray	2008-12-09
	C	159	110744.01	18206.30	120.15	(3R)-hydroxymyristoyl-acylprotein dehydratase	Lyase	Benzamidine	BEN	Protein	X-ray	2008-12-09
	C	159	110744.01	18206.30	35.45	(3R)-hydroxymyristoyl-acylprotein dehydratase	Lyase	Chloride ion	CL	Protein	X-ray	2008-12-09
	C	159	110744.01	18206.30	286.28	(3R)-hydroxymyristoyl-acylprotein dehydratase	Lyase	(2s)-5-hydroxy-2-(4-hydroxyphenyl)-7-methoxy-2,3-dihydro-4h-chromen-4-one	SAK	Protein	X-ray	2008-12-09
	D	159	110744.01	18206.30	35.45	(3R)-hydroxymyristoyl-acylprotein dehydratase	Lyase	Chloride ion	CL	Protein	X-ray	2008-12-09
	E	159	110744.01	18206.30	120.15	(3R)-hydroxymyristoyl-acylprotein dehydratase	Lyase	Benzamidine	BEN	Protein	X-ray	2008-12-09
	E	159	110744.01	18206.30	35.45	(3R)-hydroxymyristoyl-acylprotein dehydratase	Lyase	Chloride ion	CL	Protein	X-ray	2008-12-09
	F	159	110744.01	18206.30	120.15	(3R)-hydroxymyristoyl-acylprotein dehydratase	Lyase	Benzamidine	BEN	Protein	X-ray	2008-12-09
	F	159	110744.01	18206.30	35.45	(3R)-hydroxymyristoyl-acylprotein dehydratase	Lyase	Chloride ion	CL	Protein	X-ray	2008-12-09
**3DOY**	A	159	111068.44	18206.30	448.50	(3R)-hydroxymyristoyl-acylprotein dehydratase	Lyase	4-chloro-n'-[(1e)-(3,5-dibromo-2,4-dihydroxyphenyl)methylidene]benzohy	2BE	Protein	X-ray	2009-05-05

								drazide				
	A	159	111068.44	18206.30	120.15	(3R)-hydroxymyristoyl-acylprotein dehydratase	Lyase	Benzamidine	BEN	Protein	X-ray	2009-05-05
	A	159	111068.44	18206.30	35.45	(3R)-hydroxymyristoyl-acylprotein dehydratase	Lyase	Chloride ion	CL	Protein	X-ray	2009-05-05
	B	159	111068.44	18206.30	120.15	(3R)-hydroxymyristoyl-acylprotein dehydratase	Lyase	Benzamidine	BEN	Protein	X-ray	2009-05-05
	B	159	111068.44	18206.30	35.45	(3R)-hydroxymyristoyl-acylprotein dehydratase	Lyase	Chloride ion	CL	Protein	X-ray	2009-05-05
	C	159	111068.44	18206.30	448.50	(3R)-hydroxymyristoyl-acylprotein dehydratase	Lyase	4-chloro-n'-[(1e)-(3,5-dibromo-2,4-dihydroxyphenyl)methylidene]benzohydrazide	2BE	Protein	X-ray	2009-05-05
	C	159	111068.44	18206.30	120.15	(3R)-hydroxymyristoyl-acylprotein dehydratase	Lyase	Benzamidine	BEN	Protein	X-ray	2009-05-05
	C	159	111068.44	18206.30	35.45	(3R)-hydroxymyristoyl-acylprotein dehydratase	Lyase	Chloride ion	CL	Protein	X-ray	2009-05-05
	D	159	111068.44	18206.30	35.45	(3R)-hydroxymyristoyl-acylprotein dehydratase	Lyase	Chloride ion	CL	Protein	X-ray	2009-05-05
	E	159	111068.44	18206.30	120.15	(3R)-hydroxymyristoyl-acylprotein dehydratase	Lyase	Benzamidine	BEN	Protein	X-ray	2009-05-05
	E	159	111068.44	18206.30	35.45	(3R)-hydroxymyristoyl-acylprotein dehydratase	Lyase	Chloride ion	CL	Protein	X-ray	2009-05-05
	F	159	111068.44	18206.30	35.45	(3R)-hydroxymyristoyl-acylprotein dehydratase	Lyase	Chloride ion	CL	Protein	X-ray	2009-05-05
**3DOZ**	A	159	111157.34	18206.30	120.15	(3R)-hydroxymyristoyl-acylprotein dehydratase	Lyase	Benzamidine	BEN	Protein	X-ray	2009-05-05
	A	159	111157.34	18206.30	35.45	(3R)-hydroxymyristoyl-acylprotein dehydratase	Lyase	Chloride ion	CL	Protein	X-ray	2009-05-05
	B	159	111157.34	18206.30	492.95	(3R)-hydroxymyristoyl-acylprotein dehydratase	Lyase	3-bromo-n'-[(1e)-(3,5-dibromo-2,4-dihydroxyphenyl)methylidene]benzohydrazide	3BE	Protein	X-ray	2009-05-05

	B	159	111157.34	18206.30	120.15	(3R)-hydroxymyristoyl-acylprotein dehydratase	Lyase	Benzamidine	BEN	Protein	X-ray	2009-05-05
	B	159	111157.34	18206.30	35.45	(3R)-hydroxymyristoyl-acylprotein dehydratase	Lyase	Chloride ion	CL	Protein	X-ray	2009-05-05
	C	159	111157.34	18206.30	492.95	(3R)-hydroxymyristoyl-acylprotein dehydratase	Lyase	3-bromo-n'-[(1e)-(3,5-dibromo-2,4-dihydroxyphenyl)methylidene]benzohydrazide	3BE	Protein	X-ray	2009-05-05
	C	159	111157.34	18206.30	120.15	(3R)-hydroxymyristoyl-acylprotein dehydratase	Lyase	Benzamidine	BEN	Protein	X-ray	2009-05-05
	C	159	111157.34	18206.30	35.45	(3R)-hydroxymyristoyl-acylprotein dehydratase	Lyase	Chloride ion	CL	Protein	X-ray	2009-05-05
	D	159	111157.34	18206.30	35.45	(3R)-hydroxymyristoyl-acylprotein dehydratase	Lyase	Chloride ion	CL	Protein	X-ray	2009-05-05
	E	159	111157.34	18206.30	35.45	(3R)-hydroxymyristoyl-acylprotein dehydratase	Lyase	Chloride ion	CL	Protein	X-ray	2009-05-05
	F	159	111157.34	18206.30	35.45	(3R)-hydroxymyristoyl-acylprotein dehydratase	Lyase	Chloride ion	CL	Protein	X-ray	2009-05-05
**3DP0**	A	159	110755.71	18206.30	120.15	(3R)-hydroxymyristoyl-acylprotein dehydratase	Lyase	Benzamidine	BEN	Protein	X-ray	2009-05-05
	A	159	110755.71	18206.30	35.45	(3R)-hydroxymyristoyl-acylprotein dehydratase	Lyase	Chloride ion	CL	Protein	X-ray	2009-05-05
	B	159	110755.71	18206.30	464.11	(3R)-hydroxymyristoyl-acylprotein dehydratase	Lyase	N'-[(1e)-(3,5-dibromo-2,4-dihydroxyphenyl)methylidene]naphthalene-2-carbohydrazide	2BC	Protein	X-ray	2009-05-05
	B	159	110755.71	18206.30	120.15	(3R)-hydroxymyristoyl-acylprotein dehydratase	Lyase	Benzamidine	BEN	Protein	X-ray	2009-05-05
	B	159	110755.71	18206.30	35.45	(3R)-hydroxymyristoyl-acylprotein dehydratase	Lyase	Chloride ion	CL	Protein	X-ray	2009-05-05
	C	159	110755.71	18206.30	120.15	(3R)-hydroxymyristoyl-acylprotein dehydratase	Lyase	Benzamidine	BEN	Protein	X-ray	2009-05-05
	C	159	110755.71	18206.30	35.45	(3R)-hydroxymyristoyl-acylprotein	Lyase	Chloride ion	CL	Protein	X-ray	2009-05-05

						dehydratase						
	D	159	110755.71	18206.30	35.45	(3R)-hydroxymyristoyl-acylprotein dehydratase	Lyase	Chloride ion	CL	Protein	X-ray	2009-05-05
	E	159	110755.71	18206.30	120.15	(3R)-hydroxymyristoyl-acylprotein dehydratase	Lyase	Benzamidine	BEN	Protein	X-ray	2009-05-05
	E	159	110755.71	18206.30	35.45	(3R)-hydroxymyristoyl-acylprotein dehydratase	Lyase	Chloride ion	CL	Protein	X-ray	2009-05-05
	F	159	110755.71	18206.30	35.45	(3R)-hydroxymyristoyl-acylprotein dehydratase	Lyase	Chloride ion	CL	Protein	X-ray	2009-05-05
**3DP1**	A	159	110939.45	18206.30	35.45	(3R)-hydroxymyristoyl-acylprotein dehydratase	Lyase	Chloride ion	CL	Protein	X-ray	2009-05-05
	B	159	110939.45	18206.30	444.08	(3R)-hydroxymyristoyl-acylprotein dehydratase	Lyase	N'-[(1e)-(3,5-dibromo-2,4-dihydroxyphenyl)methylidene]-4-methoxybenzohydrazide	2RB	Protein	X-ray	2009-05-05
	B	159	110939.45	18206.30	120.15	(3R)-hydroxymyristoyl-acylprotein dehydratase	Lyase	Benzamidine	BEN	Protein	X-ray	2009-05-05
	B	159	110939.45	18206.30	35.45	(3R)-hydroxymyristoyl-acylprotein dehydratase	Lyase	Chloride ion	CL	Protein	X-ray	2009-05-05
	C	159	110939.45	18206.30	444.08	(3R)-hydroxymyristoyl-acylprotein dehydratase	Lyase	N'-[(1e)-(3,5-dibromo-2,4-dihydroxyphenyl)methylidene]-4-methoxybenzohydrazide	2RB	Protein	X-ray	2009-05-05
	C	159	110939.45	18206.30	35.45	(3R)-hydroxymyristoyl-acylprotein dehydratase	Lyase	Chloride ion	CL	Protein	X-ray	2009-05-05
	D	159	110939.45	18206.30	120.15	(3R)-hydroxymyristoyl-acylprotein dehydratase	Lyase	Benzamidine	BEN	Protein	X-ray	2009-05-05
	D	159	110939.45	18206.30	35.45	(3R)-hydroxymyristoyl-acylprotein dehydratase	Lyase	Chloride ion	CL	Protein	X-ray	2009-05-05
	E	159	110939.45	18206.30	120.15	(3R)-hydroxymyristoyl-acylprotein dehydratase	Lyase	Benzamidine	BEN	Protein	X-ray	2009-05-05
	E	159	110939.45	18206.30	35.45	(3R)-hydroxymyristoyl-acylprotein dehydratase	Lyase	Chloride ion	CL	Protein	X-ray	2009-05-05

	F	159	110939.45	18206.30	120.15	(3R)-hydroxymyristoyl-acylprotein dehydratase	Lyase	Benzamidine	BEN	Protein	X-ray	2009-05-05
	F	159	110939.45	18206.30	35.45	(3R)-hydroxymyristoyl-acylprotein dehydratase	Lyase	Chloride ion	CL	Protein	X-ray	2009-05-05
**3DP2**	A	159	111157.34	18206.30	120.15	(3R)-hydroxymyristoyl-acylprotein dehydratase	Lyase	Benzamidine	BEN	Protein	X-ray	2009-05-05
	A	159	111157.34	18206.30	35.45	(3R)-hydroxymyristoyl-acylprotein dehydratase	Lyase	Chloride ion	CL	Protein	X-ray	2009-05-05
	B	159	111157.34	18206.30	492.95	(3R)-hydroxymyristoyl-acylprotein dehydratase	Lyase	4-bromo-n'-[(1e)-(3,5-dibromo-2,4-dihydroxyphenyl)methylidene]benzohydrazide	4BE	Protein	X-ray	2009-05-05
	B	159	111157.34	18206.30	120.15	(3R)-hydroxymyristoyl-acylprotein dehydratase	Lyase	Benzamidine	BEN	Protein	X-ray	2009-05-05
	B	159	111157.34	18206.30	35.45	(3R)-hydroxymyristoyl-acylprotein dehydratase	Lyase	Chloride ion	CL	Protein	X-ray	2009-05-05
	C	159	111157.34	18206.30	492.95	(3R)-hydroxymyristoyl-acylprotein dehydratase	Lyase	4-bromo-n'-[(1e)-(3,5-dibromo-2,4-dihydroxyphenyl)methylidene]benzohydrazide	4BE	Protein	X-ray	2009-05-05
	C	159	111157.34	18206.30	35.45	(3R)-hydroxymyristoyl-acylprotein dehydratase	Lyase	Chloride ion	CL	Protein	X-ray	2009-05-05
	D	159	111157.34	18206.30	120.15	(3R)-hydroxymyristoyl-acylprotein dehydratase	Lyase	Benzamidine	BEN	Protein	X-ray	2009-05-05
	D	159	111157.34	18206.30	35.45	(3R)-hydroxymyristoyl-acylprotein dehydratase	Lyase	Chloride ion	CL	Protein	X-ray	2009-05-05
	E	159	111157.34	18206.30	120.15	(3R)-hydroxymyristoyl-acylprotein dehydratase	Lyase	Benzamidine	BEN	Protein	X-ray	2009-05-05
	E	159	111157.34	18206.30	35.45	(3R)-hydroxymyristoyl-acylprotein dehydratase	Lyase	Chloride ion	CL	Protein	X-ray	2009-05-05
	F	159	111157.34	18206.30	35.45	(3R)-hydroxymyristoyl-acylprotein dehydratase	Lyase	Chloride ion	CL	Protein	X-ray	2009-05-05
**3DP3**	A	159	111111.77	18206.30	470.16	(3R)-hydroxymyristoyl-acylprotein	Lyase	4-tert-butyl-n'-[(1e)-(3,5-dibromo-2,4-	4BB	Protein	X-ray	2009-05-05

						dehydratase		dihydroxyphenyl)methylidene]benzohydrazide				
	A	159	111111.77	18206.30	120.15	(3R)-hydroxymyristoyl-acylprotein dehydratase	Lyase	Benzamidine	BEN	Protein	X-ray	2009-05-05
	A	159	111111.77	18206.30	35.45	(3R)-hydroxymyristoyl-acylprotein dehydratase	Lyase	Chloride ion	CL	Protein	X-ray	2009-05-05
	B	159	111111.77	18206.30	120.15	(3R)-hydroxymyristoyl-acylprotein dehydratase	Lyase	Benzamidine	BEN	Protein	X-ray	2009-05-05
	B	159	111111.77	18206.30	35.45	(3R)-hydroxymyristoyl-acylprotein dehydratase	Lyase	Chloride ion	CL	Protein	X-ray	2009-05-05
	C	159	111111.77	18206.30	470.16	(3R)-hydroxymyristoyl-acylprotein dehydratase	Lyase	4-tert-butyl-n'-[(1e)-(3,5-dibromo-2,4-dihydroxyphenyl)methylidene]benzohydrazide	4BB	Protein	X-ray	2009-05-05
	C	159	111111.77	18206.30	120.15	(3R)-hydroxymyristoyl-acylprotein dehydratase	Lyase	Benzamidine	BEN	Protein	X-ray	2009-05-05
	C	159	111111.77	18206.30	35.45	(3R)-hydroxymyristoyl-acylprotein dehydratase	Lyase	Chloride ion	CL	Protein	X-ray	2009-05-05
	D	159	111111.77	18206.30	35.45	(3R)-hydroxymyristoyl-acylprotein dehydratase	Lyase	Chloride ion	CL	Protein	X-ray	2009-05-05
	E	159	111111.77	18206.30	120.15	(3R)-hydroxymyristoyl-acylprotein dehydratase	Lyase	Benzamidine	BEN	Protein	X-ray	2009-05-05
	E	159	111111.77	18206.30	35.45	(3R)-hydroxymyristoyl-acylprotein dehydratase	Lyase	Chloride ion	CL	Protein	X-ray	2009-05-05
	F	159	111111.77	18206.30	35.45	(3R)-hydroxymyristoyl-acylprotein dehydratase	Lyase	Chloride ion	CL	Protein	X-ray	2009-05-05
**3ED0**	A	159	110832.08	18206.30	120.15	(3R)-hydroxymyristoyl-acylprotein dehydratase	Lyase	Benzamidine	BEN	Protein	X-ray	2009-07-21
	A	159	110832.08	18206.30	35.45	(3R)-hydroxymyristoyl-acylprotein dehydratase	Lyase	Chloride ion	CL	Protein	X-ray	2009-07-21
	A	159	110832.08	18206.30	270.24	(3R)-hydroxymyristoyl-acylprotein dehydratase	Lyase	3-methyl-1,6,8-trihydroxyanthraquinone	EMO	Protein	X-ray	2009-07-21

	B	159	110832.08	18206.30	120.15	(3R)-hydroxymyristoyl-acylprotein dehydratase	Lyase	Benzamidine	BEN	Protein	X-ray	2009-07-21	
	B	159	110832.08	18206.30	35.45	(3R)-hydroxymyristoyl-acylprotein dehydratase	Lyase	Chloride ion	CL	Protein	X-ray	2009-07-21	
	C	159	110832.08	18206.30	35.45	(3R)-hydroxymyristoyl-acylprotein dehydratase	Lyase	Chloride ion	CL	Protein	X-ray	2009-07-21	
	C	159	110832.08	18206.30	270.24	(3R)-hydroxymyristoyl-acylprotein dehydratase	Lyase	3-methyl-1,6,8-trihydroxyanthraquinone	EMO	Protein	X-ray	2009-07-21	
	D	159	110832.08	18206.30	120.15	(3R)-hydroxymyristoyl-acylprotein dehydratase	Lyase	Benzamidine	BEN	Protein	X-ray	2009-07-21	
	D	159	110832.08	18206.30	35.45	(3R)-hydroxymyristoyl-acylprotein dehydratase	Lyase	Chloride ion	CL	Protein	X-ray	2009-07-21	
	E	159	110832.08	18206.30	120.15	(3R)-hydroxymyristoyl-acylprotein dehydratase	Lyase	Benzamidine	BEN	Protein	X-ray	2009-07-21	
	E	159	110832.08	18206.30	35.45	(3R)-hydroxymyristoyl-acylprotein dehydratase	Lyase	Chloride ion	CL	Protein	X-ray	2009-07-21	
	F	159	110832.08	18206.30	35.45	(3R)-hydroxymyristoyl-acylprotein dehydratase	Lyase	Chloride ion	CL	Protein	X-ray	2009-07-21	
**3EGM**	A	181	126613.58	20916.60	55.85	Ferritin	Oxidoreductase	Fe (iii) ion	FE	Protein	X-ray	2009-07-28	
	A	181	126613.58	20916.60	92.09	Ferritin	Oxidoreductase	Glycerol	GOL	Protein	X-ray	2009-07-28	
	B	181	126613.58	20916.60	55.85	Ferritin	Oxidoreductase	Fe (iii) ion	FE	Protein	X-ray	2009-07-28	
	B	181	126613.58	20916.60	92.09	Ferritin	Oxidoreductase	Glycerol	GOL	Protein	X-ray	2009-07-28	
	C	181	126613.58	20916.60	55.85	Ferritin	Oxidoreductase	Fe (iii) ion	FE	Protein	X-ray	2009-07-28	
	D	181	126613.58	20916.60	55.85	Ferritin	Oxidoreductase	Fe (iii) ion	FE	Protein	X-ray	2009-07-28	
	D	181	126613.58	20916.60	92.09	Ferritin	Oxidoreductase	Glycerol	GOL	Protein	X-ray	2009-07-28	
	E	181	126613.58	20916.60	55.85	Ferritin	Oxidoreductase	Fe (iii) ion	FE	Protein	X-ray	2009-07-28	
	F	181	126613.58	20916.60	55.85	Ferritin	Oxidoreductase	Fe (iii) ion	FE	Protein	X-ray	2009-07-28	
**3EZS**	A	376	87435.69	43374.60	62.07	Aminotransferase aspb	Transferase	1,2-ethanediol	EDO	Protein	X-ray	2008-11-18	JCSG[g]
	A	376	87435.69	43374.60	196.11	Aminotransferase aspb	Transferase	Selenomethionine	MSE	Protein	X-ray	2008-11-18	JCSG
	A	376	87435.69	43374.60	94.97	Aminotransferase aspb	Transferase	Phosphate ion	PO4	Protein	X-ray	2008-11-18	JCSG
	B	376	87435.69	43374.60	62.07	Aminotransferase aspb	Transferase	1,2-ethanediol	EDO	Protein	X-ray	2008-11-18	JCSG

	B	376	87435.69	43374.60	196.11	Aminotransferase aspb	Transferase	Selenomethionine	MSE	Protein	X-ray	2008-11-18	JCSG
	B	376	87435.69	43374.60	94.97	Aminotransferase aspb	Transferase	Phosphate ion	PO4	Protein	X-ray	2008-11-18	JCSG
**3F42**	A	99	22671.87	11211.80	62.07	Protein HP0035	SG, unknown function	1,2-ethanediol	EDO	Protein	X-ray	2008-11-18	MCSG
	A	99	22671.87	11211.80	196.11	Protein HP0035	SG, unknown function	Selenomethionine	MSE	Protein	X-ray	2008-11-18	MCSG
	B	99	22671.87	11211.80	62.07	Protein HP0035	SG, unknown function	1,2-ethanediol	EDO	Protein	X-ray	2008-11-18	MCSG
	B	99	22671.87	11211.80	196.11	Protein HP0035	SG, unknown function	Selenomethionine	MSE	Protein	X-ray	2008-11-18	MCSG
**3FC3**	A	200	52768.99	22768.40	22.99	Restriction endonuclease Hpy99I	Hydrolase/dna	Sodium ion	NA	Protein DNA	X-ray	2009-03-31	
	A	200	52768.99	22768.40	194.23	Restriction endonuclease Hpy99I	Hydrolase/dna	Tetraethylene glycol	PG4	Protein DNA	X-ray	2009-03-31	
	A	200	52768.99	22768.40	65.38	Restriction endonuclease Hpy99I	Hydrolase/dna	Zinc ion	ZN	Protein DNA	X-ray	2009-03-31	
	B	200	52768.99	22768.40	22.99	Restriction endonuclease Hpy99I	Hydrolase/dna	Sodium ion	NA	Protein DNA	X-ray	2009-03-31	
	B	200	52768.99	22768.40	65.38	Restriction endonuclease Hpy99I	Hydrolase/dna	Zinc ion	ZN	Protein DNA	X-ray	2009-03-31	
	C	11	52768.99	3358.24		5'-(*dcp*dtp*dcp*dgp*dap*dcp*dgp*dtp*dap*dgp*da)-3'	Hydrolase/dna			Protein DNA	X-ray	2009-03-31	
	D	11	52768.99	3349.23		5'-(*dtp*dap*dcp*dgp*dtp*dcp*dgp*dap*dgp*dtp*dc)-3'	Hydrolase/dna			Protein DNA	X-ray	2009-03-31	
**3FNM**	A	377	122333.35	40577.80		Gamma-glutamyltranspeptidase (Ggt) Large subunit	Transferase			Protein	X-ray	2009-05-19	
	B	188	122333.35	20410.30	178.57	Gamma-glutamyltranspeptidase (Ggt) Small subunit	Transferase	(2s)-amino[(5s)-3-chloro-4,5-dihydroisoxazol-5-yl]acetic acid	AVN	Protein	X-ray	2009-05-19	
	C	377	122333.35	40577.80		Gamma-glutamyltranspeptidase (Ggt) Large subunit	Transferase			Protein	X-ray	2009-05-19	
	D	188	122333.35	20410.30	178.57	Gamma-glutamyltranspeptidase (Ggt) Small subunit	Transferase	(2s)-amino[(5s)-3-chloro-4,5-dihydroisoxazol-5-yl]acetic acid	AVN	Protein	X-ray	2009-05-19	
**3FX7**	A	94	23207.80	11603.90		Putativeprotein	Unknown function			Protein	X-ray	2009-09-01	

	B	94	23207.80	11603.90		Putativeprotein	Unknown function			Protein	X-ray	2009-09-01
**3GAS**	A	259	184501.80	29864.20	42.02	Heme oxygenase	Oxidoreductase	Azide ion	AZI	Protein	X-ray	2010-02-23
	A	259	184501.80	29864.20	62.07	Heme oxygenase	Oxidoreductase	1,2-ethanediol	EDO	Protein	X-ray	2010-02-23
	A	259	184501.80	29864.20	616.50	Heme oxygenase	Oxidoreductase	Protoporphyrin IX containing Fe	HEM	Protein	X-ray	2010-02-23
	A	259	184501.80	29864.20	196.11	Heme oxygenase	Oxidoreductase	Selenomethionine	MSE	Protein	X-ray	2010-02-23
	B	259	184501.80	29864.20	42.02	Heme oxygenase	Oxidoreductase	Azide ion	AZI	Protein	X-ray	2010-02-23
	B	259	184501.80	29864.20	62.07	Heme oxygenase	Oxidoreductase	1,2-ethanediol	EDO	Protein	X-ray	2010-02-23
	B	259	184501.80	29864.20	616.50	Heme oxygenase	Oxidoreductase	Protoporphyrin IX containing Fe	HEM	Protein	X-ray	2010-02-23
	B	259	184501.80	29864.20	196.11	Heme oxygenase	Oxidoreductase	Selenomethionine	MSE	Protein	X-ray	2010-02-23
	C	259	184501.80	29864.20	42.02	Heme oxygenase	Oxidoreductase	Azide ion	AZI	Protein	X-ray	2010-02-23
	C	259	184501.80	29864.20	62.07	Heme oxygenase	Oxidoreductase	1,2-ethanediol	EDO	Protein	X-ray	2010-02-23
	C	259	184501.80	29864.20	616.50	Heme oxygenase	Oxidoreductase	Protoporphyrin IX containing Fe	HEM	Protein	X-ray	2010-02-23
	C	259	184501.80	29864.20	196.11	Heme oxygenase	Oxidoreductase	Selenomethionine	MSE	Protein	X-ray	2010-02-23
	D	259	184501.80	29864.20	42.02	Heme oxygenase	Oxidoreductase	Azide ion	AZI	Protein	X-ray	2010-02-23
	D	259	184501.80	29864.20	62.07	Heme oxygenase	Oxidoreductase	1,2-ethanediol	EDO	Protein	X-ray	2010-02-23
	D	259	184501.80	29864.20	616.50	Heme oxygenase	Oxidoreductase	Protoporphyrin IX containing Fe	HEM	Protein	X-ray	2010-02-23
	D	259	184501.80	29864.20	196.11	Heme oxygenase	Oxidoreductase	Selenomethionine	MSE	Protein	X-ray	2010-02-23
	E	259	184501.80	29864.20	42.02	Heme oxygenase	Oxidoreductase	Azide ion	AZI	Protein	X-ray	2010-02-23
	E	259	184501.80	29864.20	62.07	Heme oxygenase	Oxidoreductase	1,2-ethanediol	EDO	Protein	X-ray	2010-02-23
	E	259	184501.80	29864.20	616.50	Heme oxygenase	Oxidoreductase	Protoporphyrin IX containing Fe	HEM	Protein	X-ray	2010-02-23
	E	259	184501.80	29864.20	196.11	Heme oxygenase	Oxidoreductase	Selenomethionine	MSE	Protein	X-ray	2010-02-23
	F	259	184501.80	29864.20	42.02	Heme oxygenase	Oxidoreductase	Azide ion	AZI	Protein	X-ray	2010-02-23
	F	259	184501.80	29864.20	62.07	Heme oxygenase	Oxidoreductase	1,2-ethanediol	EDO	Protein	X-ray	2010-02-23
	F	259	184501.80	29864.20	616.50	Heme oxygenase	Oxidoreductase	Protoporphyrin IX containing Fe	HEM	Protein	X-ray	2010-02-23
	F	259	184501.80	29864.20	196.11	Heme oxygenase	Oxidoreductase	Selenomethionine	MSE	Protein	X-ray	2010-02-23
**3GIO**	A	186	42905.80	21452.90	196.11	Putativeprotein	Dnaprotein	Selenomethionine	MSE	Protein	X-ray	2009-08-04
	B	186	42905.80	21452.90	196.11	Putativeprotein	Dnaprotein	Selenomethionine	MSE	Protein	X-ray	2009-08-04
**3GOX**	A	200	52813.04	22768.40	238.28	Restriction endonuclease Hpy99I	Hydrolase/dna	Pentaethylene glycol	1PE	Protein DNA	X-ray	2009-04-28
	A	200	52813.04	22768.40	22.99	Restriction endonuclease Hpy99I	Hydrolase/dna	Sodium ion	NA	Protein DNA	X-ray	2009-04-28

	A	200	52813.04	22768.40	65.38	Restriction endonuclease Hpy99I	Hydrolase/dna	Zinc ion	ZN	Protein DNA	X-ray	2009-04-28
	B	200	52813.04	22768.40	22.99	Restriction endonuclease Hpy99I	Hydrolase/dna	Sodium ion	NA	Protein DNA	X-ray	2009-04-28
	B	200	52813.04	22768.40	65.38	Restriction endonuclease Hpy99I	Hydrolase/dna	Zinc ion	ZN	Protein DNA	X-ray	2009-04-28
	C	11	52813.04	3358.24		5'-(*dcp*dtp*dcp*dgp*dap*dcp*dgp*dtp*dap*dgp*da)-3'	Hydrolase/dna			Protein DNA	X-ray	2009-04-28
	D	11	52813.04	3349.23		5'-(*dtp*dap*dcp*dgp*dtp*dcp*dgp*dap*dgp*dtp*dc)-3'	Hydrolase/dna			Protein DNA	X-ray	2009-04-28
**3GUQ**	A	166	19047.00	19047.00		Putativeprotein	Toxin			Protein	X-ray	2009-09-22
**3GWG**	A	129	14585.43	14344.70	24.31	Chemotaxis protein chey homolog	Signaling protein	Magnesium ion	MG	Protein	X-ray	2010-03-09
	A	129	14585.43	14344.70	96.06	Chemotaxis protein chey homolog	Signaling protein	Sulfate ion	SO4	Protein	X-ray	2010-03-09
**3GXV**	A	123	34049.48	14291.50		Replicative DNA helicase	Hydrolase/replication			Protein	X-ray	2010-01-26
	B	123	34049.48	14291.50		Replicative DNA helicase	Hydrolase/replication			Protein	X-ray	2010-01-26
	C	22	34049.48	2490.00		Replicative DNA helicase	Hydrolase/replication			Protein	X-ray	2010-01-26
	D	26	34049.48	2976.48		Replicative DNA helicase	Hydrolase/replication			Protein	X-ray	2010-01-26
**3H1E**	A	129	14555.38	14344.70	66.01	Chemotaxis protein chey homolog	Signaling protein	Beryllium trifluoride ion	BEF	Protein	X-ray	2010-03-09
	A	129	14555.38	14344.70	24.31	Chemotaxis protein chey homolog	Signaling protein	Magnesium ion	MG	Protein	X-ray	2010-03-09
	A	129	14555.38	14344.70	96.06	Chemotaxis protein chey homolog	Signaling protein	Sulfate ion	SO4	Protein	X-ray	2010-03-09
**3H1F**	A	129	14517.12	14300.70	24.31	Chemotaxis protein chey homolog	Signaling protein	Magnesium ion	MG	Protein	X-ray	2010-03-09
	A	129	14517.12	14300.70	96.06	Chemotaxis protein chey homolog	Signaling protein	Sulfate ion	SO4	Protein	X-ray	2010-03-09
**3H1G**	A	129	14555.43	14314.70	24.31	Chemotaxis protein chey homolog	Signaling protein	Magnesium ion	MG	Protein	X-ray	2010-03-09
	A	129	14555.43	14314.70	96.06	Chemotaxis protein chey homolog	Signaling protein	Sulfate ion	SO4	Protein	X-ray	2010-03-09
**3HPE**	A	164	38000.38	18662.60	337.59	Conservedprotein	Transport protein	(13z)-docos-13-enamide	ERU	Protein	X-ray	2010-04-14
	B	164	38000.38	18662.60	337.59	Conservedprotein	Transport protein	(13z)-docos-13-enamide	ERU	Protein	X-ray	2010-04-14
**3HR7**	A	168	38723.12	19265.50	96.06	Shikimate kinase	Transferase	Sulfate ion	SO4	Protein	X-ray	2010-06-16
	B	168	38723.12	19265.50	96.06	Shikimate kinase	Transferase	Sulfate ion	SO4	Protein	X-ray	2010-06-16
**3HVM**	A	330	37614.50	37614.50		Agmatine deiminase	Hydrolase			Protein	X-ray	2010-01-19

**3IBX**	A	221	51022.20	25511.10		Putative thiaminase II	Hydrolase			Protein	X-ray	2009-11-17
	D	221	51022.20	25511.10		Putative thiaminase II	Hydrolase			Protein	X-ray	2009-11-17
**3IEC**	A	319	201373.20	36920.10		Serine/threonine-protein kinase MARK2	Signaling protein/toxin			Protein	X-ray	2009-12-08
	B	319	201373.20	36920.10		Serine/threonine-protein kinase MARK2	Signaling protein/toxin			Protein	X-ray	2009-12-08
	C	319	201373.20	36920.10		Serine/threonine-protein kinase MARK2	Signaling protein/toxin			Protein	X-ray	2009-12-08
	D	319	201373.20	36920.10		Serine/threonine-protein kinase MARK2	Signaling protein/toxin			Protein	X-ray	2009-12-08
	E	125	201373.20	13423.20		Cytotoxicity-associated immunodominant antigen	Signaling protein/toxin			Protein	X-ray	2009-12-08
	F	125	201373.20	13423.20		Cytotoxicity-associated immunodominant antigen	Signaling protein/toxin			Protein	X-ray	2009-12-08
	G	125	201373.20	13423.20		Cytotoxicity-associated immunodominant antigen	Signaling protein/toxin			Protein	X-ray	2009-12-08
	H	125	201373.20	13423.20		Cytotoxicity-associated immunodominant antigen	Signaling protein/toxin			Protein	X-ray	2009-12-08
**3IMP**	A	138	191540.41	15940.10		Chemotaxis protein motb	Membrane protein			Protein	X-ray	2010-08-04
	B	138	191540.41	15940.10		Chemotaxis protein motb	Membrane protein			Protein	X-ray	2010-08-04
	C	138	191540.41	15940.10		Chemotaxis protein motb	Membrane protein			Protein	X-ray	2010-08-04
	D	138	191540.41	15940.10		Chemotaxis protein motb	Membrane protein			Protein	X-ray	2010-08-04
	E	138	191540.41	15940.10		Chemotaxis protein motb	Membrane protein			Protein	X-ray	2010-08-04
	F	138	191540.41	15940.10		Chemotaxis protein motb	Membrane protein			Protein	X-ray	2010-08-04
	G	138	191540.41	15940.10	35.45	Chemotaxis protein motb	Membrane protein	Chloride ion	CL	Protein	X-ray	2010-08-04
	G	138	191540.41	15940.10	58.70	Chemotaxis protein motb	Membrane protein	Nickel (ii) ion	NI	Protein	X-ray	2010-08-04
	H	138	191540.41	15940.10		Chemotaxis protein motb	Membrane protein			Protein	X-ray	2010-08-04
	I	138	191540.41	15940.10		Chemotaxis protein motb	Membrane protein			Protein	X-ray	2010-08-04
	J	138	191540.41	15940.10		Chemotaxis protein motb	Membrane protein			Protein	X-ray	2010-08-04
	K	138	191540.41	15940.10	35.45	Chemotaxis protein motb	Membrane protein	Chloride ion	CL	Protein	X-ray	2010-08-04
	K	138	191540.41	15940.10	58.70	Chemotaxis protein motb	Membrane protein	Nickel (ii) ion	NI	Protein	X-ray	2010-08-04

	L	138	191540.41	15940.10		Chemotaxis protein motb	Membrane protein			Protein	X-ray	2010-08-04	
**3IQC**	A	131	29940.20	14970.10		Flagellar protein	Chaperone			Protein	X-ray	2010-06-30	
	B	131	29940.20	14970.10		Flagellar protein	Chaperone			Protein	X-ray	2010-06-30	
**3ISH**	A	311	103089.17	33577.50	785.56	Thioredoxin reductase	Oxidoreductase	Flavin-adenine dinucleotide	FAD	Protein	X-ray	2009-10-06	
	B	311	103089.17	33577.50	785.56	Thioredoxin reductase	Oxidoreductase	Flavin-adenine dinucleotide	FAD	Protein	X-ray	2009-10-06	
	C	311	103089.17	33577.50	785.56	Thioredoxin reductase	Oxidoreductase	Flavin-adenine dinucleotide	FAD	Protein	X-ray	2009-10-06	
**3JUJ**	A	281	128348.80	32087.20		UDP-glucose pyrophosphorylase (galu)	Transferase			Protein	X-ray	2010-03-31	
	B	281	128348.80	32087.20		UDP-glucose pyrophosphorylase (galu)	Transferase			Protein	X-ray	2010-03-31	
	C	281	128348.80	32087.20		UDP-glucose pyrophosphorylase (galu)	Transferase			Protein	X-ray	2010-03-31	
	D	281	128348.80	32087.20		UDP-glucose pyrophosphorylase (galu)	Transferase			Protein	X-ray	2010-03-31	
**3JUK**	A	281	130905.68	32087.20	24.31	UDP-glucose pyrophosphorylase (galu)	Transferase	Magnesium ion	MG	Protein	X-ray	2010-03-31	
	A	281	130905.68	32087.20	566.30	UDP-glucose pyrophosphorylase (galu)	Transferase	Uridine-5'-diphosphate-glucose	UPG	Protein	X-ray	2010-03-31	
	B	281	130905.68	32087.20	24.31	UDP-glucose pyrophosphorylase (galu)	Transferase	Magnesium ion	MG	Protein	X-ray	2010-03-31	
	B	281	130905.68	32087.20	566.30	UDP-glucose pyrophosphorylase (galu)	Transferase	Uridine-5'-diphosphate-glucose	UPG	Protein	X-ray	2010-03-31	
	C	281	130905.68	32087.20	24.31	UDP-glucose pyrophosphorylase (galu)	Transferase	Magnesium ion	MG	Protein	X-ray	2010-03-31	
	C	281	130905.68	32087.20	566.30	UDP-glucose pyrophosphorylase (galu)	Transferase	Uridine-5'-diphosphate-glucose	UPG	Protein	X-ray	2010-03-31	
	D	281	130905.68	32087.20	24.31	UDP-glucose pyrophosphorylase (galu)	Transferase	Magnesium ion	MG	Protein	X-ray	2010-03-31	
	D	281	130905.68	32087.20	566.30	UDP-glucose pyrophosphorylase (galu)	Transferase	Uridine-5'-diphosphate-glucose	UPG	Protein	X-ray	2010-03-31	
**3K1H**	A	158	17989.70	17989.70		Putativeprotein	Chaperone			Protein	X-ray	2010-06-30	
**3K1I**	A	131	70472.80	14970.10		Flagellar protein	Chaperone			Protein	X-ray	2010-06-30	
	B	131	70472.80	14970.10		Flagellar protein	Chaperone			Protein	X-ray	2010-06-30	
	C	178	70472.80	20266.30		Putativeprotein	Chaperone			Protein	X-ray	2010-06-30	
	D	178	70472.80	20266.30		Putativeprotein	Chaperone			Protein	X-ray	2010-06-30	
**3K5W**	A	475	52510.07	52415.10	196.11	Carbohydrate kinase	Transferase	Selenomethionine	MSE	Protein	X-ray	2009-12-08	NYSRCSG
	A	475	52510.07	52415.10	94.97	Carbohydrate kinase	Transferase	Phosphate ion	PO4	Protein	X-ray	2009-12-08	NYSRCSG
**3KU7**	A	80	18431.50	9215.75		Cell division topological specificity factor	Cell cycle			Protein	X-ray	2010-05-05	
	B	80	18431.50	9215.75		Cell division topological specificity factor	Cell cycle			Protein	X-ray	2010-05-05	
**3KWL**	A	514	60116.00	60116.00	196.11	Uncharacterized protein	Unknown function	Selenomethionine	MSE	Protein	X-ray	2010-12-01	
**3L9Z**	A	170	19412.70	19412.70		Ureaseprotein uree	Metalprotein			Protein	X-ray	2010-08-25	MKBSGI

**3LA0**	A	170	77650.80	19412.70		Ureaseprotein uree	Metalprotein	Unknown atom or ion	UNX	Protein	X-ray	2010-08-25	MKBSGI
	B	170	77650.80	19412.70		Ureaseprotein uree	Metalprotein			Protein	X-ray	2010-08-25	MKBSGI
	C	170	77650.80	19412.70		Ureaseprotein uree	Metalprotein			Protein	X-ray	2010-08-25	MKBSGI
	D	170	77650.80	19412.70		Ureaseprotein uree	Metalprotein			Protein	X-ray	2010-08-25	MKBSGI
**3LGH**	A	148	68940.71	17170.40	24.31	Nickel-responsive regulator	Metalprotein	Magnesium ion	MG	Protein	X-ray	2010-09-15	
	A	148	68940.71	17170.40	58.70	Nickel-responsive regulator	Metalprotein	Nickel (ii) ion	NI	Protein	X-ray	2010-09-15	
	B	148	68940.71	17170.40	58.70	Nickel-responsive regulator	Metalprotein	Nickel (ii) ion	NI	Protein	X-ray	2010-09-15	
	C	148	68940.71	17170.40	58.70	Nickel-responsive regulator	Metalprotein	Nickel (ii) ion	NI	Protein	X-ray	2010-09-15	
	D	148	68940.71	17170.40	58.70	Nickel-responsive regulator	Metalprotein	Nickel (ii) ion	NI	Protein	X-ray	2010-09-15	
**3LLW**	A	311	142026.94	35290.60	96.06	Geranyltranstransferase (ispa)	Transferase	Sulfate ion	SO4	Protein	X-ray	2010-03-31	NYSRCSG
	B	311	142026.94	35290.60	96.06	Geranyltranstransferase (ispa)	Transferase	Sulfate ion	SO4	Protein	X-ray	2010-03-31	NYSRCSG
	C	311	142026.94	35290.60	96.06	Geranyltranstransferase (ispa)	Transferase	Sulfate ion	SO4	Protein	X-ray	2010-03-31	NYSRCSG
	D	311	142026.94	35290.60	96.06	Geranyltranstransferase (ispa)	Transferase	Sulfate ion	SO4	Protein	X-ray	2010-03-31	NYSRCSG
**3M1Y**	A	217	98561.19	24565.60	35.45	Phosphoserine phosphatase (serb)	Hydrolase	Chloride ion	CL	Protein	X-ray	2010-03-23	NYSRCSG
	A	217	98561.19	24565.60	24.31	Phosphoserine phosphatase (serb)	Hydrolase	Magnesium ion	MG	Protein	X-ray	2010-03-23	NYSRCSG
	A	217	98561.19	24565.60	196.11	Phosphoserine phosphatase (serb)	Hydrolase	Selenomethionine	MSE	Protein	X-ray	2010-03-23	NYSRCSG
	B	217	98561.19	24565.60	35.45	Phosphoserine phosphatase (serb)	Hydrolase	Chloride ion	CL	Protein	X-ray	2010-03-23	NYSRCSG
	B	217	98561.19	24565.60	24.31	Phosphoserine phosphatase (serb)	Hydrolase	Magnesium ion	MG	Protein	X-ray	2010-03-23	NYSRCSG
	B	217	98561.19	24565.60	196.11	Phosphoserine phosphatase (serb)	Hydrolase	Selenomethionine	MSE	Protein	X-ray	2010-03-23	NYSRCSG
	C	217	98561.19	24565.60	35.45	Phosphoserine phosphatase (serb)	Hydrolase	Chloride ion	CL	Protein	X-ray	2010-03-23	NYSRCSG
	C	217	98561.19	24565.60	24.31	Phosphoserine phosphatase (serb)	Hydrolase	Magnesium ion	MG	Protein	X-ray	2010-03-23	NYSRCSG
	C	217	98561.19	24565.60	196.11	Phosphoserine phosphatase (serb)	Hydrolase	Selenomethionine	MSE	Protein	X-ray	2010-03-23	NYSRCSG
	D	217	98561.19	24565.60	35.45	Phosphoserine phosphatase (serb)	Hydrolase	Chloride ion	CL	Protein	X-ray	2010-03-23	NYSRCSG
	D	217	98561.19	24565.60	24.31	Phosphoserine phosphatase (serb)	Hydrolase	Magnesium ion	MG	Protein	X-ray	2010-03-23	NYSRCSG
	D	217	98561.19	24565.60	196.11	Phosphoserine phosphatase (serb)	Hydrolase	Selenomethionine	MSE	Protein	X-ray	2010-03-23	NYSRCSG
**3M21**	A	67	44336.70	7389.45		Probable tautomerase HP_0924	Isomerase			Protein	X-ray	2010-09-01	
	B	67	44336.70	7389.45		Probable tautomerase HP_0924	Isomerase			Protein	X-ray	2010-09-01	
	C	67	44336.70	7389.45		Probable tautomerase HP_0924	Isomerase			Protein	X-ray	2010-09-01	
	D	67	44336.70	7389.45		Probable tautomerase HP_0924	Isomerase			Protein	X-ray	2010-09-01	
	E	67	44336.70	7389.45		Probable tautomerase HP_0924	Isomerase			Protein	X-ray	2010-09-01	
	F	67	44336.70	7389.45		Probable tautomerase HP_0924	Isomerase			Protein	X-ray	2010-09-01	

**3MCD**	A	80	18431.50	9215.75		Cell division topological specificity factor	Cell cycle			Protein	X-ray	2010-05-05	
	B	80	18431.50	9215.75		Cell division topological specificity factor	Cell cycle			Protein	X-ray	2010-05-05	
**3MLE**	A	242	167606.38	27148.30	159.23	Dethiobiotin synthetase	Ligase	8-aminooctanoic acid	8AC	Protein	X-ray	2010-05-19	MCSG
	A	242	167606.38	27148.30	427.20	Dethiobiotin synthetase	Ligase	Adenosine-5'-diphosphate	ADP	Protein	X-ray	2010-05-19	MCSG
	A	242	167606.38	27148.30	35.45	Dethiobiotin synthetase	Ligase	Chloride ion	CL	Protein	X-ray	2010-05-19	MCSG
	A	242	167606.38	27148.30	24.31	Dethiobiotin synthetase	Ligase	Magnesium ion	MG	Protein	X-ray	2010-05-19	MCSG
	A	242	167606.38	27148.30	62.01	Dethiobiotin synthetase	Ligase	Nitrate ion	NO3	Protein	X-ray	2010-05-19	MCSG
	A	242	167606.38	27148.30	94.97	Dethiobiotin synthetase	Ligase	Phosphate ion	PO4	Protein	X-ray	2010-05-19	MCSG
	B	242	167606.38	27148.30	159.23	Dethiobiotin synthetase	Ligase	8-aminooctanoic acid	8AC	Protein	X-ray	2010-05-19	MCSG
	B	242	167606.38	27148.30	427.20	Dethiobiotin synthetase	Ligase	Adenosine-5'-diphosphate	ADP	Protein	X-ray	2010-05-19	MCSG
	B	242	167606.38	27148.30	24.31	Dethiobiotin synthetase	Ligase	Magnesium ion	MG	Protein	X-ray	2010-05-19	MCSG
	B	242	167606.38	27148.30	62.01	Dethiobiotin synthetase	Ligase	Nitrate ion	NO3	Protein	X-ray	2010-05-19	MCSG
	B	242	167606.38	27148.30	94.97	Dethiobiotin synthetase	Ligase	Phosphate ion	PO4	Protein	X-ray	2010-05-19	MCSG
	C	242	167606.38	27148.30	159.23	Dethiobiotin synthetase	Ligase	8-aminooctanoic acid	8AC	Protein	X-ray	2010-05-19	MCSG
	C	242	167606.38	27148.30	427.20	Dethiobiotin synthetase	Ligase	Adenosine-5'-diphosphate	ADP	Protein	X-ray	2010-05-19	MCSG
	C	242	167606.38	27148.30	35.45	Dethiobiotin synthetase	Ligase	Chloride ion	CL	Protein	X-ray	2010-05-19	MCSG
	C	242	167606.38	27148.30	24.31	Dethiobiotin synthetase	Ligase	Magnesium ion	MG	Protein	X-ray	2010-05-19	MCSG
	C	242	167606.38	27148.30	94.97	Dethiobiotin synthetase	Ligase	Phosphate ion	PO4	Protein	X-ray	2010-05-19	MCSG
	D	242	167606.38	27148.30	159.23	Dethiobiotin synthetase	Ligase	8-aminooctanoic acid	8AC	Protein	X-ray	2010-05-19	MCSG
	D	242	167606.38	27148.30	427.20	Dethiobiotin synthetase	Ligase	Adenosine-5'-diphosphate	ADP	Protein	X-ray	2010-05-19	MCSG
	D	242	167606.38	27148.30	24.31	Dethiobiotin synthetase	Ligase	Magnesium ion	MG	Protein	X-ray	2010-05-19	MCSG
	D	242	167606.38	27148.30	94.97	Dethiobiotin synthetase	Ligase	Phosphate ion	PO4	Protein	X-ray	2010-05-19	MCSG
	E	242	167606.38	27148.30	159.23	Dethiobiotin synthetase	Ligase	8-aminooctanoic acid	8AC	Protein	X-ray	2010-05-19	MCSG
	E	242	167606.38	27148.30	427.20	Dethiobiotin synthetase	Ligase	Adenosine-5'-diphosphate	ADP	Protein	X-ray	2010-05-19	MCSG
	E	242	167606.38	27148.30	24.31	Dethiobiotin synthetase	Ligase	Magnesium ion	MG	Protein	X-ray	2010-05-19	MCSG
	E	242	167606.38	27148.30	94.97	Dethiobiotin synthetase	Ligase	Phosphate ion	PO4	Protein	X-ray	2010-05-19	MCSG
	F	242	167606.38	27148.30	159.23	Dethiobiotin synthetase	Ligase	8-aminooctanoic acid	8AC	Protein	X-ray	2010-05-19	MCSG
	F	242	167606.38	27148.30	427.20	Dethiobiotin synthetase	Ligase	Adenosine-5'-diphosphate	ADP	Protein	X-ray	2010-05-19	MCSG
	F	242	167606.38	27148.30	35.45	Dethiobiotin synthetase	Ligase	Chloride ion	CL	Protein	X-ray	2010-05-19	MCSG

	F	242	167606.38	27148.30	24.31	Dethiobiotin synthetase	Ligase	Magnesium ion	MG	Protein	X-ray	2010-05-19	MCSG
	F	242	167606.38	27148.30	62.01	Dethiobiotin synthetase	Ligase	Nitrate ion	NO3	Protein	X-ray	2010-05-19	MCSG
	F	242	167606.38	27148.30	94.97	Dethiobiotin synthetase	Ligase	Phosphate ion	PO4	Protein	X-ray	2010-05-19	MCSG
**3MLG**	A	189	43924.40	21962.20		Putativeprotein,Protein	Unknown function			Protein	X-ray	2010-05-12	
	B	189	43924.40	21962.20		Putativeprotein,Protein	Unknown function			Protein	X-ray	2010-05-12	
**3MLI**	A	100	47758.96	11919.70		Putativeprotein	Unknown function			Protein	X-ray	2010-05-12	
	B	100	47758.96	11919.70		Putativeprotein	Unknown function			Protein	X-ray	2010-05-12	
	C	100	47758.96	11919.70		Putativeprotein	Unknown function			Protein	X-ray	2010-05-12	
	D	100	47758.96	11919.70	40.08	Putativeprotein	Unknown function	Calcium ion	CA	Protein	X-ray	2010-05-12	
**3MRS**	A	168	19179.40	19179.40		Shikimate kinase	Transferase			Protein	X-ray	2011-05-04	
**3MUF**	A	168	19946.84	19265.50	427.20	Shikimate kinase	Transferase	Adenosine-5'-diphosphate	ADP	Protein	X-ray	2011-05-04	
	A	168	19946.84	19265.50	254.13	Shikimate kinase	Transferase	Shikimate-3-phosphate	S3P	Protein	X-ray	2011-05-04	
**3MYD**	A	365	40972.80	40972.80		Flagellarprotein flha	Protein transport			Protein	X-ray	2010-05-26	
**3N2E**	A	168	58742.29	19004.30	489.47	Shikimate kinase	Transferase	7-amino-4-hydroxy-3-[(e)-(5-hydroxy-7-sulfonaphthalen-2-yl)diazenyl]naphthalene-2-sulfonic acid	OSA	Protein	X-ray	2011-05-18	
**3N2E**	A	168	58742.29	19004.30	150.09	Shikimate kinase	Transferase	L(+)-tartaric acid	TLA	Protein	X-ray	2011-05-18	
	B	168	58742.29	19004.30	489.47	Shikimate kinase	Transferase	7-amino-4-hydroxy-3-[(e)-(5-hydroxy-7-sulfonaphthalen-2-yl)diazenyl]naphthalene-2-sulfonic acid	OSA	Protein	X-ray	2011-05-18	
	B	168	58742.29	19004.30	150.09	Shikimate kinase	Transferase	L(+)-tartaric acid	TLA	Protein	X-ray	2011-05-18	
	C	168	58742.29	19004.30		Shikimate kinase	Transferase			Protein	X-ray	2011-05-18	
**3N3Y**	A	216	104173.55	24872.60	785.56	Thymidylate synthase thyx	Transferase	Flavin-adenine dinucleotide	FAD	Protein	X-ray	2011-05-25	
	A	216	104173.55	24872.60	308.18	Thymidylate synthase thyx	Transferase	2'-deoxyuridine 5'-monophosphate	UMP	Protein	X-ray	2011-05-25	
	B	216	104173.55	24872.60	785.56	Thymidylate synthase thyx	Transferase	Flavin-adenine dinucleotide	FAD	Protein	X-ray	2011-05-25	
	B	216	104173.55	24872.60	308.18	Thymidylate synthase thyx	Transferase	2'-deoxyuridine 5'-monophosphate	UMP	Protein	X-ray	2011-05-25	
	C	216	104173.55	24872.60	785.56	Thymidylate synthase thyx	Transferase	Flavin-adenine dinucleotide	FAD	Protein	X-ray	2011-05-25	
	C	216	104173.55	24872.60	308.18	Thymidylate synthase thyx	Transferase	2'-deoxyuridine 5'-monophosphate	UMP	Protein	X-ray	2011-05-25	
	D	216	104173.55	24872.60	785.56	Thymidylate synthase thyx	Transferase	Flavin-adenine dinucleotide	FAD	Protein	X-ray	2011-05-25	

	D	216	104173.55	24872.60	308.18	Thymidylate synthase thyx	Transferase	2'-deoxyuridine 5'-monophosphate	UMP	Protein	X-ray	2011-05-25
**3N9R**	A	307	273344.62	33801.10	35.45	Fructose-bisphosphate aldolase	Lyase	Chloride ion	CL	Protein	X-ray	2010-11-17
	A	307	273344.62	33801.10	22.99	Fructose-bisphosphate aldolase	Lyase	Sodium ion	NA	Protein	X-ray	2010-11-17
	A	307	273344.62	33801.10	243.15	Fructose-bisphosphate aldolase	Lyase	2-[hydroxy(4-hydroxybutyl)amino]-2-oxoethyl dihydrogen phosphate	TD3	Protein	X-ray	2010-11-17
	A	307	273344.62	33801.10	65.38	Fructose-bisphosphate aldolase	Lyase	Zinc ion	ZN	Protein	X-ray	2010-11-17
	B	307	273344.62	33801.10	35.45	Fructose-bisphosphate aldolase	Lyase	Chloride ion	CL	Protein	X-ray	2010-11-17
	B	307	273344.62	33801.10	22.99	Fructose-bisphosphate aldolase	Lyase	Sodium ion	NA	Protein	X-ray	2010-11-17
	B	307	273344.62	33801.10	243.15	Fructose-bisphosphate aldolase	Lyase	2-[hydroxy(4-hydroxybutyl)amino]-2-oxoethyl dihydrogen phosphate	TD3	Protein	X-ray	2010-11-17
	B	307	273344.62	33801.10	65.38	Fructose-bisphosphate aldolase	Lyase	Zinc ion	ZN	Protein	X-ray	2010-11-17
	e	307	273344.62	33801.10	35.45	Fructose-bisphosphate aldolase	Lyase	Chloride ion	CL	Protein	X-ray	2010-11-17
	e	307	273344.62	33801.10	22.99	Fructose-bisphosphate aldolase	Lyase	Sodium ion	NA	Protein	X-ray	2010-11-17
	e	307	273344.62	33801.10	243.15	Fructose-bisphosphate aldolase	Lyase	2-[hydroxy(4-hydroxybutyl)amino]-2-oxoethyl dihydrogen phosphate	TD3	Protein	X-ray	2010-11-17
	e	307	273344.62	33801.10	65.38	Fructose-bisphosphate aldolase	Lyase	Zinc ion	ZN	Protein	X-ray	2010-11-17
	j	307	273344.62	33801.10	35.45	Fructose-bisphosphate aldolase	Lyase	Chloride ion	CL	Protein	X-ray	2010-11-17
	j	307	273344.62	33801.10	22.99	Fructose-bisphosphate aldolase	Lyase	Sodium ion	NA	Protein	X-ray	2010-11-17
	j	307	273344.62	33801.10	243.15	Fructose-bisphosphate aldolase	Lyase	2-[hydroxy(4-hydroxybutyl)amino]-2-oxoethyl dihydrogen phosphate	TD3	Protein	X-ray	2010-11-17
	j	307	273344.62	33801.10	65.38	Fructose-bisphosphate aldolase	Lyase	Zinc ion	ZN	Protein	X-ray	2010-11-17
	K	307	273344.62	33801.10	35.45	Fructose-bisphosphate aldolase	Lyase	Chloride ion	CL	Protein	X-ray	2010-11-17
	K	307	273344.62	33801.10	22.99	Fructose-bisphosphate aldolase	Lyase	Sodium ion	NA	Protein	X-ray	2010-11-17
	K	307	273344.62	33801.10	243.15	Fructose-bisphosphate aldolase	Lyase	2-[hydroxy(4-hydroxybutyl)amino]-2-oxoethyl dihydrogen phosphate	TD3	Protein	X-ray	2010-11-17
	K	307	273344.62	33801.10	65.38	Fructose-bisphosphate aldolase	Lyase	Zinc ion	ZN	Protein	X-ray	2010-11-17
	P	307	273344.62	33801.10	35.45	Fructose-bisphosphate aldolase	Lyase	Chloride ion	CL	Protein	X-ray	2010-11-17
	P	307	273344.62	33801.10	22.99	Fructose-bisphosphate aldolase	Lyase	Sodium ion	NA	Protein	X-ray	2010-11-17
	P	307	273344.62	33801.10	243.15	Fructose-bisphosphate aldolase	Lyase	2-[hydroxy(4-hydroxybutyl)amino]-2-oxoethyl dihydrogen phosphate	TD3	Protein	X-ray	2010-11-17
	P	307	273344.62	33801.10	65.38	Fructose-bisphosphate aldolase	Lyase	Zinc ion	ZN	Protein	X-ray	2010-11-17

	U	307	273344.62	33801.10	35.45	Fructose-bisphosphate aldolase	Lyase	Chloride ion	CL	Protein	X-ray	2010-11-17
	U	307	273344.62	33801.10	22.99	Fructose-bisphosphate aldolase	Lyase	Sodium ion	NA	Protein	X-ray	2010-11-17
	U	307	273344.62	33801.10	243.15	Fructose-bisphosphate aldolase	Lyase	2-[hydroxy(4-hydroxybutyl)amino]-2-oxoethyl dihydrogen phosphate	TD3	Protein	X-ray	2010-11-17
	U	307	273344.62	33801.10	65.38	Fructose-bisphosphate aldolase	Lyase	Zinc ion	ZN	Protein	X-ray	2010-11-17
	Z	307	273344.62	33801.10	35.45	Fructose-bisphosphate aldolase	Lyase	Chloride ion	CL	Protein	X-ray	2010-11-17
	Z	307	273344.62	33801.10	22.99	Fructose-bisphosphate aldolase	Lyase	Sodium ion	NA	Protein	X-ray	2010-11-17
	Z	307	273344.62	33801.10	243.15	Fructose-bisphosphate aldolase	Lyase	2-[hydroxy(4-hydroxybutyl)amino]-2-oxoethyl dihydrogen phosphate	TD3	Protein	X-ray	2010-11-17
	Z	307	273344.62	33801.10	65.38	Fructose-bisphosphate aldolase	Lyase	Zinc ion	ZN	Protein	X-ray	2010-11-17
**3N9S**	A	307	68505.37	33801.10	40.08	Fructose-bisphosphate aldolase	Lyase/lyase inhibitor	Calcium ion	CA	Protein	X-ray	2010-11-17
	A	307	68505.37	33801.10	22.99	Fructose-bisphosphate aldolase	Lyase/lyase inhibitor	Sodium ion	NA	Protein	X-ray	2010-11-17
	A	307	68505.37	33801.10	323.13	Fructose-bisphosphate aldolase	Lyase/lyase inhibitor	4-(hydroxy[(phosphonooxy)acetyl]amino)butyl dihydrogen phosphate	TD4	Protein	X-ray	2010-11-17
	A	307	68505.37	33801.10	65.38	Fructose-bisphosphate aldolase	Lyase/lyase inhibitor	Zinc ion	ZN	Protein	X-ray	2010-11-17
	B	307	68505.37	33801.10	40.08	Fructose-bisphosphate aldolase	Lyase/lyase inhibitor	Calcium ion	CA	Protein	X-ray	2010-11-17
	B	307	68505.37	33801.10	22.99	Fructose-bisphosphate aldolase	Lyase/lyase inhibitor	Sodium ion	NA	Protein	X-ray	2010-11-17
	B	307	68505.37	33801.10	323.13	Fructose-bisphosphate aldolase	Lyase/lyase inhibitor	4-(hydroxy[(phosphonooxy)acetyl]amino)butyl dihydrogen phosphate	TD4	Protein	X-ray	2010-11-17
	B	307	68505.37	33801.10	65.38	Fructose-bisphosphate aldolase	Lyase/lyase inhibitor	Zinc ion	ZN	Protein	X-ray	2010-11-17
**3NA7**	A	256	30127.29	29799.30	238.30	HP0958	Gene regulation, chaperone	4-(2-hydroxyethyl)-1-piperazine ethanesulfonic acid	EPE	Protein	X-ray	2010-09-22
	A	256	30127.29	29799.30	24.31	HP0958	Gene regulation, chaperone	Magnesium ion	MG	Protein	X-ray	2010-09-22
	A	256	30127.29	29799.30	65.38	HP0958	Gene regulation, chaperone	Zinc ion	ZN	Protein	X-ray	2010-09-22
**3NM4**	A	230	50594.75	25050.10	62.07	MTA/SAH nucleosidase	Hydrolase	1,2-ethanediol	EDO	Protein	X-ray	2010-11-24
	A	230	50594.75	25050.10	122.14	MTA/SAH nucleosidase	Hydrolase	2-amino-2-hydroxymethyl-propane-1,3-diol	TRS	Protein	X-ray	2010-11-24

	B	230	50594.75	25050.10	62.07	MTA/SAH nucleosidase	Hydrolase	1,2-ethanediol	EDO	Protein	X-ray	2010-11-24	
**3NM5**	A	230	50634.69	25050.10	267.24	MTA/SAH nucleosidase	Hydrolase	(1s)-1-(7-amino-1h-pyrazolo[4,3-d]pyrimidin-3-yl)-1,4-anhydro-d-ribitol	FMC	Protein	X-ray	2010-11-24	
	B	230	50634.69	25050.10	267.24	MTA/SAH nucleosidase	Hydrolase	(1s)-1-(7-amino-1h-pyrazolo[4,3-d]pyrimidin-3-yl)-1,4-anhydro-d-ribitol	FMC	Protein	X-ray	2010-11-24	
	B	230	25555.64	25050.10	135.13	MTA/SAH nucleosidase	Hydrolase	Adenine	ADE	Protein	X-ray	2010-11-24	
	B	230	25555.64	25050.10	62.07	MTA/SAH nucleosidase	Hydrolase	1,2-ethanediol	EDO	Protein	X-ray	2010-11-24	
	B	230	25555.64	25050.10	122.14	MTA/SAH nucleosidase	Hydrolase	2-amino-2-hydroxymethyl-propane-1,3-diol	TRS	Protein	X-ray	2010-11-24	
**3NV7**	A	157	18171.08	17726.80	60.05	Phosphopantetheine adenylyltransferase	Transferase	Acetic acid	ACY	Protein	X-ray	2011-07-13	
	A	157	18171.08	17726.80	96.06	Phosphopantetheine adenylyltransferase	Transferase	Sulfate ion	SO4	Protein	X-ray	2011-07-13	
**3NXZ**	A	170	77714.34	19412.70	63.55	UreaseProtein ureE	Metalprotein	Copper (ii) ion	CU	Protein	X-ray	2010-08-25	MKBSGI
	B	170	77714.34	19412.70		UreaseProtein ureE	Metalprotein			Protein	X-ray	2010-08-25	MKBSGI
	C	170	77714.34	19412.70		UreaseProtein ureE	Metalprotein			Protein	X-ray	2010-08-25	MKBSGI
	D	170	77714.34	19412.70		UreaseProtein ureE	Metalprotein			Protein	X-ray	2010-08-25	MKBSGI
**3NY0**	A	170	77709.50	19412.70		UreaseProtein ureE	Metalprotein			Protein	X-ray	2010-08-25	MKBSGI
	B	170	77709.50	19412.70	58.70	UreaseProtein ureE	Metalprotein	Nickel (ii) ion	NI	Protein	X-ray	2010-08-25	MKBSGI
	C	170	77709.50	19412.70		UreaseProtein ureE	Metalprotein			Protein	X-ray	2010-08-25	MKBSGI
	D	170	77709.50	19412.70		UreaseProtein ureE	Metalprotein			Protein	X-ray	2010-08-25	MKBSGI
**3O1Q**	A	254	85956.00	28652.00		UreaseProtein ureF	Metalprotein			Protein	X-ray	2010-08-18	
	B	254	85956.00	28652.00		UreaseProtein ureF	Metalprotein			Protein	X-ray	2010-08-18	
	C	254	85956.00	28652.00		UreaseProtein ureF	Metalprotein			Protein	X-ray	2010-08-18	
**3OQG**	A	180	47812.51	21130.20	35.45	Hpy188I	Hydrolase/dna	Chloride ion	CL	Protein DNA	X-ray	2010-10-20	
	A	180	47812.51	21130.20	196.11	Hpy188I	Hydrolase/dna	Selenomethionine	MSE	Protein DNA	X-ray	2010-10-20	
	A	180	47812.51	21130.20	22.99	Hpy188I	Hydrolase/dna	Sodium ion	NA	Protein DNA	X-ray	2010-10-20	
	B	180	47812.51	21130.20	196.11	Hpy188I	Hydrolase/dna	Selenomethionine	MSE	Protein	X-ray	2010-10-20	

										DNA		
	B	180	47812.51	21130.20	22.99	Hpy188I	Hydrolase/dna	Sodium ion	NA	Protein DNA	X-ray	2010-10-20
	C	9	47812.51	2739.85		DNA 5'-D(*GP*AP*TP*CP*TP*GP*AP*AP*C)-3'	Hydrolase/dna			Protein DNA	X-ray	2010-10-20
	D	9	47812.51	2730.83		DNA 5'-D(*GP*TP*TP*CP*AP*GP*AP*TP*C)-3'	Hydrolase/dna			Protein DNA	X-ray	2010-10-20
**3OR3**	A	180	47903.22	21130.20	40.08	Restriction endonuclease hpy188i	Hydrolase/dna	Calcium ion	CA	Protein DNA	X-ray	2010-10-20
	A	180	47903.22	21130.20	35.45	Restriction endonuclease hpy188i	Hydrolase/dna	Chloride ion	CL	Protein DNA	X-ray	2010-10-20
	A	180	47903.22	21130.20	196.11	Restriction endonuclease hpy188i	Hydrolase/dna	Selenomethionine	MSE	Protein DNA	X-ray	2010-10-20
	B	180	47903.22	21130.20	35.45	Restriction endonuclease hpy188i	Hydrolase/dna	Chloride ion	CL	Protein DNA	X-ray	2010-10-20
	B	180	47903.22	21130.20	196.11	Restriction endonuclease hpy188i	Hydrolase/dna	Selenomethionine	MSE	Protein DNA	X-ray	2010-10-20
	C	5	47903.22	1495.04		5'-D(*GP*AP*TP*CP*T)-3'	Hydrolase/dna			Protein DNA	X-ray	2010-10-20
	D	5	47903.22	1495.04		5'-D(*GP*TP*TP*CP*A)-3'	Hydrolase/dna			Protein DNA	X-ray	2010-10-20
	E	4	47903.22	1199.85		5'-D(P*GP*AP*AP*C)-3'	Hydrolase/dna			Protein DNA	X-ray	2010-10-20
	F	4	47903.22	1190.84		5'-D(P*GP*AP*TP*C)-3'	Hydrolase/dna			Protein DNA	X-ray	2010-10-20
**3OTW**	A	163	116401.20	18520.60	767.53	Phosphopantetheine adenylyltransferase	Transferase	Coenzyme a	COA	Protein	X-ray	2011-09-14
	A	163	116401.20	18520.60	96.06	Phosphopantetheine adenylyltransferase	Transferase	Sulfate ion	SO4	Protein	X-ray	2011-09-14

	B	163	116401.20	18520.60	767.53	Phosphopantetheine adenylyltransferase	Transferase	Coenzyme a	COA	Protein	X-ray	2011-09-14	
	B	163	116401.20	18520.60	96.06	Phosphopantetheine adenylyltransferase	Transferase	Sulfate ion	SO4	Protein	X-ray	2011-09-14	
	C	163	116401.20	18520.60	767.53	Phosphopantetheine adenylyltransferase	Transferase	Coenzyme a	COA	Protein	X-ray	2011-09-14	
	C	163	116401.20	18520.60	96.06	Phosphopantetheine adenylyltransferase	Transferase	Sulfate ion	SO4	Protein	X-ray	2011-09-14	
	D	163	116401.20	18520.60	767.53	Phosphopantetheine adenylyltransferase	Transferase	Coenzyme a	COA	Protein	X-ray	2011-09-14	
	D	163	116401.20	18520.60	96.06	Phosphopantetheine adenylyltransferase	Transferase	Sulfate ion	SO4	Protein	X-ray	2011-09-14	
	E	163	116401.20	18520.60	767.53	Phosphopantetheine adenylyltransferase	Transferase	Coenzyme a	COA	Protein	X-ray	2011-09-14	
	E	163	116401.20	18520.60	96.06	Phosphopantetheine adenylyltransferase	Transferase	Sulfate ion	SO4	Protein	X-ray	2011-09-14	
	F	163	116401.20	18520.60	767.53	Phosphopantetheine adenylyltransferase	Transferase	Coenzyme a	COA	Protein	X-ray	2011-09-14	
**3P9Z**	A	229	26411.45	26309.40	102.05	Uroporphyrinogen III cosynthase (HemD)	Ligase	Malonate ion	MLI	Protein	X-ray	2010-11-03	MCSG
	A	229	26411.45	26309.40	196.11	Uroporphyrinogen III cosynthase (HemD)	Ligase	Selenomethionine	MSE	Protein	X-ray	2010-11-03	MCSG
**3PHG**	A	269	60278.40	30139.20		Shikimate dehydrogenase	Oxidoreductase			Protein	X-ray	2011-11-09	
	B	269	60278.40	30139.20		Shikimate dehydrogenase	Oxidoreductase			Protein	X-ray	2011-11-09	
**3PHH**	A	269	30313.35	30139.20	174.15	Shikimate dehydrogenase	Oxidoreductase	(3r,4s,5r)-3,4,5-trihydroxycyclohex-1-ene-1-carboxylic acid	SKM	Protein	X-ray	2011-11-09	
**3PHI**	A	269	62117.56	30139.20	745.43	Shikimate dehydrogenase	Oxidoreductase	Nadph dihydro-nicotinamide-adenine-dinucleotide phosphate	NDP	Protein	X-ray	2011-11-09	
	A	269	62117.56	30139.20	174.15	Shikimate dehydrogenase	Oxidoreductase	(3r,4s,5r)-3,4,5-trihydroxycyclohex-1-ene-1-carboxylic acid	SKM	Protein	X-ray	2011-11-09	
	B	269	62117.56	30139.20	745.43	Shikimate dehydrogenase	Oxidoreductase	Nadph dihydro-nicotinamide-adenine-	NDP	Protein	X-ray	2011-11-09	

								dinucleotide phosphate						
	B	269	62117.56	30139.20	174.15	Shikimate dehydrogenase	Oxidoreductase	(3r,4s,5r)-3,4,5-trihydroxycyclohex-1-ene-1-carboxylic acid	SKM	Protein	X-ray	2011-11-09	
**3PHJ**	A	269	60626.70	30139.20	174.15	Shikimate dehydrogenase	Oxidoreductase	3-dehydroshikimate	DHK	Protein	X-ray	2011-11-09	
	B	269	60626.70	30139.20	174.15	Shikimate dehydrogenase	Oxidoreductase	3-dehydroshikimate	DHK	Protein	X-ray	2011-11-09	
**3PHT**	A	148	34324.00	17103.30	58.70	Putative nickel-responsive regulator	Transcription	Nickel (ii) ion	NI	Protein	X-ray	2011-11-23	
	B	148	34324.00	17103.30	58.70	Putative nickel-responsive regulator	Transcription	Nickel (ii) ion	NI	Protein	X-ray	2011-11-23	
**3Q1O**	A	310	143464.69	35175.50	246.09	Geranyltranstransferase (IspA)	Transferase	3-methylbut-3-enyl trihydrogen diphosphate	IPE	Protein	X-ray	2011-01-12	NYSRCSG
	A	310	143464.69	35175.50	24.31	Geranyltranstransferase (IspA)	Transferase	Magnesium ion	MG	Protein	X-ray	2011-01-12	NYSRCSG
	A	310	143464.69	35175.50	96.06	Geranyltranstransferase (IspA)	Transferase	Sulfate ion	SO4	Protein	X-ray	2011-01-12	NYSRCSG
	B	310	143464.69	35175.50	246.09	Geranyltranstransferase (IspA)	Transferase	3-methylbut-3-enyl trihydrogen diphosphate	IPE	Protein	X-ray	2011-01-12	NYSRCSG
	B	310	143464.69	35175.50	24.31	Geranyltranstransferase (IspA)	Transferase	Magnesium ion	MG	Protein	X-ray	2011-01-12	NYSRCSG
	B	310	143464.69	35175.50	96.06	Geranyltranstransferase (IspA)	Transferase	Sulfate ion	SO4	Protein	X-ray	2011-01-12	NYSRCSG
	C	310	143464.69	35175.50	246.09	Geranyltranstransferase (IspA)	Transferase	3-methylbut-3-enyl trihydrogen diphosphate	IPE	Protein	X-ray	2011-01-12	NYSRCSG
	C	310	143464.69	35175.50	24.31	Geranyltranstransferase (IspA)	Transferase	Magnesium ion	MG	Protein	X-ray	2011-01-12	NYSRCSG
	C	310	143464.69	35175.50	96.06	Geranyltranstransferase (IspA)	Transferase	Sulfate ion	SO4	Protein	X-ray	2011-01-12	NYSRCSG
	D	310	143464.69	35175.50	246.09	Geranyltranstransferase (IspA)	Transferase	3-methylbut-3-enyl trihydrogen diphosphate	IPE	Protein	X-ray	2011-01-12	NYSRCSG
	D	310	143464.69	35175.50	24.31	Geranyltranstransferase (IspA)	Transferase	Magnesium ion	MG	Protein	X-ray	2011-01-12	NYSRCSG
**3QBU**	A	326	150530.31	37567.20	65.38	PutativeProtein	Hydrolase	Zinc ion	ZN	Protein	X-ray	2011-05-25	
	B	326	150530.31	37567.20	65.38	PutativeProtein	Hydrolase	Zinc ion	ZN	Protein	X-ray	2011-05-25	
	C	326	150530.31	37567.20	65.38	PutativeProtein	Hydrolase	Zinc ion	ZN	Protein	X-ray	2011-05-25	
	D	326	150530.31	37567.20	65.38	PutativeProtein	Hydrolase	Zinc ion	ZN	Protein	X-ray	2011-05-25	
**3QDL**	A	210	98414.38	24101.20	456.35	Oxygen-insensitive NADPH nitroreductase	Oxidoreductase	Flavin mononucleotide	FMN	Protein	X-ray	2012-01-18	
**3QDL**	A	210	98414.38	24101.20	92.09	Oxygen-insensitive NADPH nitroreductase	Oxidoreductase	Glycerol	GOL	Protein	X-ray	2012-01-18	
	B	210	98414.38	24101.20	456.35	Oxygen-insensitive NADPH	Oxidoreductase	Flavin mononucleotide	FMN	Protein	X-ray	2012-01-18	

						nitroreductase							
	C	210	98414.38	24101.20	456.35	Oxygen-insensitive NADPH nitroreductase	Oxidoreductase	Flavin mononucleotide	FMN	Protein	X-ray	2012-01-18	
	D	210	98414.38	24101.20	456.35	Oxygen-insensitive NADPH nitroreductase	Oxidoreductase	Flavin mononucleotide	FMN	Protein	X-ray	2012-01-18	
	D	210	98414.38	24101.20	92.09	Oxygen-insensitive NADPH nitroreductase	Oxidoreductase	Glycerol	GOL	Protein	X-ray	2012-01-18	
**3QHP**	A	166	37157.20	18578.60		TypeProtein J (CapJ)	Transferase			Protein	X-ray	2011-06-29	
	B	166	37157.20	18578.60		TypeProtein J (CapJ)	Transferase			Protein	X-ray	2011-06-29	
**3QXC**	A	242	28684.82	27148.30	507.18	Dethiobiotin synthetase	Ligase	Adenosine-5'-triphosphate	ATP	Protein	X-ray	2011-03-30	MCSG
	A	242	28684.82	27148.30	62.07	Dethiobiotin synthetase	Ligase	1,2-ethanediol	EDO	Protein	X-ray	2011-03-30	MCSG
	A	242	28684.82	27148.30	92.09	Dethiobiotin synthetase	Ligase	Glycerol	GOL	Protein	X-ray	2011-03-30	MCSG
	A	242	28684.82	27148.30	24.31	Dethiobiotin synthetase	Ligase	Magnesium ion	MG	Protein	X-ray	2011-03-30	MCSG
	A	242	28684.82	27148.30	62.01	Dethiobiotin synthetase	Ligase	Nitrate ion	NO3	Protein	X-ray	2011-03-30	MCSG
	A	242	28684.82	27148.30	106.12	Dethiobiotin synthetase	Ligase	Di(hydroxyethyl)ether	PEG	Protein	X-ray	2011-03-30	MCSG
**3QXH**	A	242	28188.65	27148.30	159.23	Dethiobiotin synthetase	Ligase	8-aminooctanoic acid	8AC	Protein	X-ray	2011-03-30	MCSG
	A	242	28188.65	27148.30	427.20	Dethiobiotin synthetase	Ligase	Adenosine-5'-diphosphate	ADP	Protein	X-ray	2011-03-30	MCSG
	A	242	28188.65	27148.30	62.07	Dethiobiotin synthetase	Ligase	1,2-ethanediol	EDO	Protein	X-ray	2011-03-30	MCSG
	A	242	28188.65	27148.30	24.31	Dethiobiotin synthetase	Ligase	Magnesium ion	MG	Protein	X-ray	2011-03-30	MCSG
	A	242	28188.65	27148.30	94.97	Dethiobiotin synthetase	Ligase	Phosphate ion	PO4	Protein	X-ray	2011-03-30	MCSG
**3QXJ**	A	242	28316.41	27148.30	62.07	Dethiobiotin synthetase	Ligase	1,2-ethanediol	EDO	Protein	X-ray	2011-03-30	MCSG
	A	242	28316.41	27148.30	523.18	Dethiobiotin synthetase	Ligase	Guanosine-5'-triphosphate	GTP	Protein	X-ray	2011-03-30	MCSG
	A	242	28316.41	27148.30	24.31	Dethiobiotin synthetase	Ligase	Magnesium ion	MG	Protein	X-ray	2011-03-30	MCSG
	A	242	28316.41	27148.30	62.01	Dethiobiotin synthetase	Ligase	Nitrate ion	NO3	Protein	X-ray	2011-03-30	MCSG
**3QXS**	A	242	28423.56	27148.30	506.20	Dethiobiotin synthetase	Ligase	Phosphoaminophosphonic acid-adenylate ester	ANP	Protein	X-ray	2011-03-30	MCSG
	A	242	28423.56	27148.30	62.07	Dethiobiotin synthetase	Ligase	1,2-ethanediol	EDO	Protein	X-ray	2011-03-30	MCSG
	A	242	28423.56	27148.30	24.31	Dethiobiotin synthetase	Ligase	Magnesium ion	MG	Protein	X-ray	2011-03-30	MCSG
	A	242	28423.56	27148.30	62.01	Dethiobiotin synthetase	Ligase	Nitrate ion	NO3	Protein	X-ray	2011-03-30	MCSG
**3QXX**	A	242	28358.81	27148.30	159.23	Dethiobiotin synthetase	Ligase	8-aminooctanoic acid	8AC	Protein	X-ray	2011-03-30	MCSG
	A	242	28358.81	27148.30	62.07	Dethiobiotin synthetase	Ligase	1,2-ethanediol	EDO	Protein	X-ray	2011-03-30	MCSG

	A	242	28358.81	27148.30	443.20	Dethiobiotin synthetase	Ligase	Guanosine-5'-diphosphate	GDP	Protein	X-ray	2011-03-30	MCSG
	A	242	28358.81	27148.30	92.09	Dethiobiotin synthetase	Ligase	Glycerol	GOL	Protein	X-ray	2011-03-30	MCSG
	A	242	28358.81	27148.30	24.31	Dethiobiotin synthetase	Ligase	Magnesium ion	MG	Protein	X-ray	2011-03-30	MCSG
	A	242	28358.81	27148.30	94.97	Dethiobiotin synthetase	Ligase	Phosphate ion	PO4	Protein	X-ray	2011-03-30	MCSG
**3QY0**	A	242	28728.17	27148.30	62.07	Dethiobiotin synthetase	Ligase	1,2-ethanediol	EDO	Protein	X-ray	2011-03-30	MCSG
	A	242	28728.17	27148.30	443.20	Dethiobiotin synthetase	Ligase	Guanosine-5'-diphosphate	GDP	Protein	X-ray	2011-03-30	MCSG
	A	242	28728.17	27148.30	24.31	Dethiobiotin synthetase	Ligase	Magnesium ion	MG	Protein	X-ray	2011-03-30	MCSG
	A	242	28728.17	27148.30	94.97	Dethiobiotin synthetase	Ligase	Phosphate ion	PO4	Protein	X-ray	2011-03-30	MCSG
**3RPF**	A	148	51184.51	16988.50	62.07	Molybdopterin synthase catalytic subunit	Transferase	1,2-ethanediol	EDO	Protein	X-ray	2011-06-29	MCSG
	A	148	51184.51	16988.50	196.11	Molybdopterin synthase catalytic subunit	Transferase	Selenomethionine	MSE	Protein	X-ray	2011-06-29	MCSG
	A	148	51184.51	16988.50	96.06	Molybdopterin synthase catalytic subunit	Transferase	Sulfate ion	SO4	Protein	X-ray	2011-06-29	MCSG
	B	148	51184.51	16988.50	62.07	Molybdopterin synthase catalytic subunit	Transferase	1,2-ethanediol	EDO	Protein	X-ray	2011-06-29	MCSG
	B	148	51184.51	16988.50	196.11	Molybdopterin synthase catalytic subunit	Transferase	Selenomethionine	MSE	Protein	X-ray	2011-06-29	MCSG
	B	148	51184.51	16988.50	96.06	Molybdopterin synthase catalytic subunit	Transferase	Sulfate ion	SO4	Protein	X-ray	2011-06-29	MCSG
	C	74	51184.51	8383.56	196.11	Molybdopterin converting factor, subunit 1 (MoaD)	Transferase	Selenomethionine	MSE	Protein	X-ray	2011-06-29	MCSG
	D	74	51184.51	8383.56	196.11	Molybdopterin converting factor, subunit 1 (MoaD)	Transferase	Selenomethionine	MSE	Protein	X-ray	2011-06-29	MCSG
**3RRL**	A	235	97219.09	25806.50	196.11	Succinyl-CoA:3-ketoacid-coenzyme A transferase subunit A	Transferase	Selenomethionine	MSE	Protein	X-ray	2011-06-29	MCSG
	B	207	97219.09	22757.00	196.11	Succinyl-CoA:3-ketoacid-coenzyme A transferase subunit B	Transferase	Selenomethionine	MSE	Protein	X-ray	2011-06-29	MCSG
	C	235	97219.09	25806.50	92.09	Succinyl-CoA:3-ketoacid-coenzyme A transferase subunit A	Transferase	Glycerol	GOL	Protein	X-ray	2011-06-29	MCSG
	C	235	97219.09	25806.50	196.11	Succinyl-CoA:3-ketoacid-coenzyme A	Transferase	Selenomethionine	MSE	Protein	X-ray	2011-06-29	MCSG

						transferase subunit A							
	D	207	97219.09	22757.00	196.11	Succinyl-CoA:3-ketoacid-coenzyme A transferase subunit B	Transferase	Selenomethionine	MSE	Protein	X-ray	2011-06-29	MCSG
**3SF5**	A	254	118438.17	28652.00	92.09	UreaseProtein ureF	Chaperone	Glycerol	GOL	Protein	X-ray	2011-11-02	
	A	254	118438.17	28652.00	106.12	UreaseProtein ureF	Chaperone	Di(hydroxyethyl)ether	PEG	Protein	X-ray	2011-11-02	
	B	265	118438.17	29757.30	106.12	UreaseProtein ureH	Chaperone	Di(hydroxyethyl)ether	PEG	Protein	X-ray	2011-11-02	
	B	265	118438.17	29757.30	96.06	UreaseProtein ureH	Chaperone	Sulfate ion	SO4	Protein	X-ray	2011-11-02	
	C	254	118438.17	28652.00	92.09	UreaseProtein ureF	Chaperone	Glycerol	GOL	Protein	X-ray	2011-11-02	
	C	254	118438.17	28652.00	106.12	UreaseProtein ureF	Chaperone	Di(hydroxyethyl)ether	PEG	Protein	X-ray	2011-11-02	
	C	254	118438.17	28652.00	96.06	UreaseProtein ureF	Chaperone	Sulfate ion	SO4	Protein	X-ray	2011-11-02	
	D	265	118438.17	29757.30	106.12	UreaseProtein ureH	Chaperone	Di(hydroxyethyl)ether	PEG	Protein	X-ray	2011-11-02	
	D	265	118438.17	29757.30	96.06	UreaseProtein ureH	Chaperone	Sulfate ion	SO4	Protein	X-ray	2011-11-02	
**3SXP**	A	362	209058.16	41148.20	663.43	ADP-L-glycero-D-mannoheptose-6-epimerase	Isomerase	Nicotinamide-adenine-dinucleotide	NAD	Protein	X-ray	2012-02-08	
	B	362	209058.16	41148.20	663.43	ADP-L-glycero-D-mannoheptose-6-epimerase	Isomerase	Nicotinamide-adenine-dinucleotide	NAD	Protein	X-ray	2012-02-08	
	C	362	209058.16	41148.20	663.43	ADP-L-glycero-D-mannoheptose-6-epimerase	Isomerase	Nicotinamide-adenine-dinucleotide	NAD	Protein	X-ray	2012-02-08	
	D	362	209058.16	41148.20	663.43	ADP-L-glycero-D-mannoheptose-6-epimerase	Isomerase	Nicotinamide-adenine-dinucleotide	NAD	Protein	X-ray	2012-02-08	
	E	362	209058.16	41148.20	663.43	ADP-L-glycero-D-mannoheptose-6-epimerase	Isomerase	Nicotinamide-adenine-dinucleotide	NAD	Protein	X-ray	2012-02-08	
**3T9J**	A	164	19068.87	19006.80	62.07	Neutrophil-activating protein	Metal transport	1,2-ethanediol	EDO	Protein	X-ray	2012-02-08	
**3TA8**	A	164	19366.77	19006.80	62.07	Neutrophil-activating protein	Metal transport	1,2-ethanediol	EDO	Protein	X-ray	2012-02-08	
	A	164	19366.77	19006.80	55.85	Neutrophil-activating protein	Metal transport	Fe (III) ion	FE	Protein	X-ray	2012-02-08	
**3TC1**	A	315	72308.81	36130.10	24.31	Octaprenyl Pyrophosphate Synthase	Transferase	Magnesium ion	MG	Protein	X-ray	2011-08-31	
	B	315	72308.81	36130.10	24.31	Octaprenyl Pyrophosphate Synthase	Transferase	Magnesium ion	MG	Protein	X-ray	2011-08-31	
**3TDG**	A	273	30991.95	30571.50	46.03	PutativeProtein	Oxidoreductase	Formic acid	FMT	Protein	X-ray	2011-11-09	
	A	273	30991.95	30571.50	92.09	PutativeProtein	Oxidoreductase	Glycerol	GOL	Protein	X-ray	2011-11-09	
	A	273	30991.95	30571.50	282.33	PutativeProtein	Oxidoreductase	Hexaethylene glycol	P6G	Protein	X-ray	2011-11-09	
**3TJ8**	A	170	39394.36	19437.70	46.03	UreaseProtein ureE	metalProtein	Formic acid	FMT	Protein	X-ray	2011-11-02	

	B	170	39394.36	19437.70	46.03	UreaseProtein ureE	metalProtein	Formic acid	FMT	Protein	X-ray	2011-11-02
	B	170	39394.36	19437.70	58.70	UreaseProtein ureE	metalProtein	Nickel (ii) ion	NI	Protein	X-ray	2011-11-02
**3TJ9**	A	170	77927.58	19437.70	46.03	UreaseProtein ureE	metalProtein	Formic acid	FMT	Protein	X-ray	2011-11-02
	A	170	77927.58	19437.70	65.38	UreaseProtein ureE	metalProtein	Zinc ion	ZN	Protein	X-ray	2011-11-02
	B	170	77927.58	19437.70		UreaseProtein ureE	metalProtein			Protein	X-ray	2011-11-02
	C	170	77927.58	19437.70	65.38	UreaseProtein ureE	metalProtein	Zinc ion	ZN	Protein	X-ray	2011-11-02
	D	170	77927.58	19437.70		UreaseProtein ureE	metalProtein			Protein	X-ray	2011-11-02
**3TJA**	A	170	78170.48	19437.70	35.45	UreaseProtein ureE	metalProtein	Chloride ion	CL	Protein	X-ray	2011-11-02
	A	170	78170.48	19437.70	96.06	UreaseProtein ureE	metalProtein	Sulfate ion	SO4	Protein	X-ray	2011-11-02
	B	170	78170.48	19437.70	96.06	UreaseProtein ureE	metalProtein	Sulfate ion	SO4	Protein	X-ray	2011-11-02
	C	170	78170.48	19437.70	96.06	UreaseProtein ureE	metalProtein	Sulfate ion	SO4	Protein	X-ray	2011-11-02
	D	170	78170.48	19437.70	96.06	UreaseProtein ureE	Metalprotein	Sulfate ion	SO4	Protein	X-ray	2011-11-02
**3TWO**	A	348	79226.37	38737.00	745.43	Mannitol dehydrogenase	Oxidoreductase	Nadph dihydro-nicotinamide-adenine-dinucleotide phosphate	NDP	Protein	X-ray	2011-10-12
	A	348	79226.37	38737.00	65.38	Mannitol dehydrogenase	Oxidoreductase	Zinc ion	ZN	Protein	X-ray	2011-10-12
	B	348	79226.37	38737.00	745.43	Mannitol dehydrogenase	Oxidoreductase	Nadph dihydro-nicotinamide-adenine-dinucleotide phosphate	NDP	Protein	X-ray	2011-10-12
	B	348	79226.37	38737.00	65.38	Mannitol dehydrogenase	Oxidoreductase	Zinc ion	ZN	Protein	X-ray	2011-10-12
**3UI3**	A	160	37445.60	18722.80	196.11	ImmunoglobulinProteinProtein D	Rnaprotein	Selenomethionine	MSE	Protein	X-ray	2012-02-08
	B	160	37445.60	18722.80	196.11	ImmunoglobulinProteinProtein D	Rnaprotein	Selenomethionine	MSE	Protein	X-ray	2012-02-08
**3USW**	A	258	29018.70	29018.70		FlagellarProtein	Motor protein			Protein	X-ray	2011-12-14
**3USY**	A	234	52240.80	26120.40		FlagellarProtein	Motor protein			Protein	X-ray	2011-12-14
	B	234	52240.80	26120.40		FlagellarProtein	Motor protein			Protein	X-ray	2011-12-14

[a] Midwest Center for SG; [b] Structural Genomics; [c] Northeast SG Consortium; [d] Montreal-Kingston Bacterial Structural Genomics Initiative; [e] New York SGX Research Center for SG; [f] Ontario Centre for Structural Proteomics; [g] Joint Center for SG.
